# National, sub-national, and risk-attributed burden of thyroid cancer in Iran from 1990 to 2019

**DOI:** 10.1038/s41598-022-17115-0

**Published:** 2022-08-02

**Authors:** Mohammadreza Azangou-Khyavy, Sahar Saeedi Moghaddam, Negar Rezaei, Zahra Esfahani, Nazila Rezaei, Sina Azadnajafabad, Mohammad-Mahdi Rashidi, Esmaeil Mohammadi, GBD 2019 Iran Thyroid Cancer Collaborators, Mohammadreza Azangou-Khyavy, Sahar Saeedi Moghaddam, Negar Rezaei, Zahra Esfahani, Nazila Rezaei, Sina Azadnajafabad, Mohammad-Mahdi Rashidi, Esmaeil Mohammadi, Mohsen Abbasi-Kangevari, Zeinab Abbasi-Kangevari, Hassan Abolhassani, Sepideh Ahmadi, Ali Ahmadi, Saeed Amini, Fazel Isapanah Amlashi, Ali Arash Anoushirvani, Jalal Arabloo, Seyyed Shamsadin Athari, Amirhossein Azari Jafari, Sima Besharat, Ali Bijani, Ahmad Daryani, Mostafa Dianatinasab, Mojtaba Didehdar, Ali Fatehizadeh, Seyyed-Hadi Ghamari, Ahmad Ghashghaee, Pouya Goleij, Mohamad Golitaleb, Nima Hafezi-Nejad, Arvin Haj-Mirzaian, Soheil Hassanipour, Ali Kabir, Maryam Keramati, Rovshan Khalilov, Maryam Khayamzadeh, Ali-Asghar Kolahi, Farzad Kompani, Hamid Reza Koohestani, Somayeh Livani, Soleiman Mahjoub, Mohammad-Reza Malekpour, Narges Malih, Borhan Mansouri, Entezar Mehrabi Nasab, Seyyedmohammadsadeq Mirmoeeni, Abdollah Mohammadian-Hafshejani, Reza Mohammadpourhodki, Sara Momtazmanesh, Mohammadreza Naghipour, Houshang Najafi, Javad Nazari, Seyed Aria Nejadghaderi, Maryam Noori, Ali Nowroozi, Fatemeh Pashazadeh Kan, Raffaele Pezzani, Sima Rafiei, Samira Raoofi, Mohammad Sadegh Razeghinia, Maryam Rezaei, Saeid Rezaei, Nima Rezaei, Sahba Rezazadeh-Khadem, Farhad Saeedi, Maryam Sahebazzamani, Amirhossein Sahebkar, Saeed Shahabi, Javad Sharifi-Rad, Sara Sheikhbahaei, Reza Shirkoohi, Parnian Shobeiri, Rohollah Valizadeh, Iman Zare, Seyed Mohammad Tavangar, Hamidreza Jamshidi, Ali H. Mokdad, Mohsen Naghavi, Farshad Farzadfar, Bagher Larijani, Seyed Mohammad Tavangar, Hamidreza Jamshidi, Ali H. Mokdad, Mohsen Naghavi, Farshad Farzadfar, Bagher Larijani

**Affiliations:** 1 Non-Communicable Diseases Research Center, Endocrinology and Metabolism Population Sciences Institute, Tehran University of Medical Sciences, Tehran, Iran; 2 Endocrinology and Metabolism Research Center, Endocrinology and Metabolism Clinical Sciences Institute, Tehran University of Medical Sciences, Tehran, Iran; 3 Department of Pathology, Shariati Hospital, Tehran University of Medical Sciences, Tehran, Iran; 4 Department of Pharmacology, School of Medicine, Shahid Beheshti University of Medical Sciences, Tehran, Iran; 5 Department of Health Metrics Sciences, School of Medicine, University of Washington, Seattle, WA USA; 6 Institute for Health Metrics and Evaluation, University of Washington, Seattle, WA USA; 7 Research Center for Immunodeficiencies, Tehran University of Medical Sciences, Tehran, Iran; 8 Department of Biosciences and Nutrition, Karolinska University Hospital, Huddinge, Sweden; 9 School of Advanced Technologies in Medicine, Shahid Beheshti University of Medical Sciences, Tehran, Iran; 10 Department of Epidemiology and Biostatistics, Shahrekord University of Medical Sciences, Shahrekord, Iran; 11 Department of Epidemiology, Shahid Beheshti University of Medical Sciences, Tehran, Iran; 12 Department of Health Services Management, Khomein University of Medical Sciences, Khomein, Iran; 13 Neuroscience Research Center, Golestan University of Medical Sciences, Gorgan, Iran; 14 Department of Internal Medicine, Iran University of Medical Sciences, Tehran, Iran; 15 Health Management and Economics Research Center, Iran University of Medical Sciences, Tehran, Iran; 16 Department of Immunology, Zanjan University of Medical Sciences, Zanjan, Iran; 17 School of Medicine, Shahroud University of Medical Sciences, Shahroud, Iran; 18 Golestan Research Center of Gastroentrology and Hepatology, Golestan University of Medical Sciences, Gorgan, Iran; 19 Social Determinants of Health Research Center, Babol University of Medical Sciences, Babol, Iran; 20 Toxoplasmosis Research Center, Mazandaran University of Medical Sciences, Sari, Iran; 21 Department of Epidemiology, Maastricht University, Maastricht, The Netherlands; 22 Department of Epidemiology, Shiraz University of Medical Sciences, Shiraz, Iran; 23 Department of Parasitology and Mycology, Arak University of Medical Sciences, Arak, Iran; 24 Department of Environmental Health Engineering, Isfahan University of Medical Sciences, Isfahan, Iran; 25 School of Public Health, Qazvin University of Medical Sciences, Qazvin, Iran; 26 Department of Genetics, Sana Institute of Higher Education, Sari, Iran; 27 Department of Nursing, Arak University of Medical Sciences, Arak, Iran; 28 Department of Radiology and Radiological Science, Johns Hopkins University, Baltimore, MD USA; 29 School of Medicine, Tehran University of Medical Sciences, Tehran, Iran; 30 Department of Pharmacology, Shahid Beheshti University of Medical Sciences, Tehran, Iran; 31 Obesity Research Center, Shahid Beheshti University of Medical Sciences, Tehran, Iran; 32 Gastrointestinal and Liver Diseases Research Center, Guilan University of Medical Sciences, Rasht, Iran; 33 Caspian Digestive Disease Research Center, Guilan University of Medical Sciences, Rasht, Iran; 34 Minimally Invasive Surgery Research Center, Iran University of Medical Sciences, Tehran, Iran; 35 Mashhad University of Medical Sciences, Mashhad, Iran; 36 Department of Biophysics and Biochemistry, Baku State University, Baku, Azerbaijan; 37 Russian Institute for Advanced Study, Moscow State Pedagogical University, Moscow, Russia; 38 Shahid Beheshti University of Medical Sciences, Tehran, Iran; 39 The Iranian Academy of Medical Sciences, Tehran, Iran; 40 Department of Health & Community Medicine, Shahid Beheshti University of Medical Sciences, Tehran, Iran; 41 Social Determinants of Health Research Center, Shahid Beheshti University of Medical Sciences, Tehran, Iran; 42 Children’s Medical Center, Tehran University of Medical Sciences, Tetran, Iran; 43 Social Determinants of Health Research Center, Saveh University of Medical Sciences, Saveh, Iran; 44 Radiology Department, Golestan University of Medical Sciences, Gorgan, Iran; 45 Cellular and Molecular Biology Research Center, Health Research Institute, Babol University of Medical Sciences, Babol, Iran; 46 Department of Clinical Biochemistry, Babol University of Medical Sciences, Babol, Iran; 47 Department of Community Medicine, Shahid Beheshti University of Medical Sciences, Tehran, Iran; 48 Substance Abuse Prevention Research Center, Kermanshah University of Medical Sciences, Kermanshah, Iran; 49 Tehran Heart Center, Tehran University of Medical Sciences, Tehran, Iran; 50 Department of Nursing, Mashhad University of Medical Sciences, Mashhad, Iran; 51 Department of Physiology, Kermanshah University of Medical Sciences, Kermanshah, Iran; 52 Department of Pediatrics, Arak University of Medical Sciences, Arak, Iran; 53 School of Medicine, Shahid Beheshti University of Medical Sciences, Tehran, Iran; 54 Student Research Committee, Iran University of Medical Sciences, Tehran, Iran; 55 Iran University of Medical Sciences, Tehran, Iran; 56 Department of Medicine, Endocrinology Unit, University of Padova, Padova, Italy; 57 AIROB (Associazione Italiana Ricerca Oncologica di Base), Padova, Italy; 58 Social Determinants of Health Research Center, Qazvin University of Medical Sciences, Qazvin, Iran; 59 Independent Consultant, Tehran, Iran; 60 Department of Immunology and Laboratory Sciences, Sirjan School of Medical Sciences, Sirjan, Iran; 61 Department of Immunology, Kerman University of Medical Sciences, Kerman, Iran; 62 Medical Toxicology & Drug Abuse Research Center, Birjand University of Medical Sciences, Birjand, Iran; 63 The Five Senses Health Institute, Iran University of Medical Sciences, Tehran, Iran; 64 Eye and Skull Base Research Centers, Rassoul Akram Hospital, Tehran, Iran; 65 Network of Immunity in Infection, Malignancy and Autoimmunity (NIIMA), Universal Scientific Education and Research Network (USERN), Tehran, Iran; 66 Cardiovascular Diseases Research Center, Birjand University of Medical Sciences, Birjand, Iran; 67 Department of Medical Biochemistry, Rafsanjan University of Medical Sciences, Rafsanjan, Iran; 68 Medical Laboratory Sciences, Sirjan School of Medical Sciences, Sirjan, Iran; 69 Applied Biomedical Research Center, Mashhad University of Medical Sciences, Mashhad, Iran; 70 Biotechnology Research Center, Mashhad University of Medical Sciences, Mashhad, Iran; 71 Health Policy Research Center, Shiraz University of Medical Sciences, Shiraz, Iran; 72 Phytochemistry Research Center, Shahid Beheshti University of Medical Sciences, Tehran, Iran; 73 Cancer Research Center, Tehran University of Medical Sciences, Tehran, Iran; 74 Cancer Biology Research Center, Tehran University of Medical Sciences, Tehran, Iran; 75 Department of Epidemiology, School of Public Health, Iran University of Medical Sciences, Tehran, Iran; 76 Research and Development Department, Sina Medical Biochemistry Technologies, Shiraz, Iran

**Keywords:** Cancer epidemiology, Health policy

## Abstract

An updated exploration of the burden of thyroid cancer across a country is always required for making correct decisions. The objective of this study is to present the thyroid cancer burden and attributed burden to the high Body Mass Index (BMI) in Iran at national and sub-national levels from 1990 to 2019. The data was obtained from the GBD 2019 study estimates. To explain the pattern of changes in incidence from 1990 to 2019, decomposition analysis was conducted. Besides, the attribution of high BMI in the thyroid cancer DALYs and deaths were obtained. The age-standardized incidence rate of thyroid cancer was 1.57 (95% UI: 1.33–1.86) in 1990 and increased 131% (53–191) until 2019. The age-standardized prevalence rate of thyroid cancer was 30.19 (18.75–34.55) in 2019 which increased 164% (77–246) from 11.44 (9.38–13.85) in 1990. In 2019, the death rate, and Disability-adjusted life years of thyroid cancer was 0.49 (0.36–0.53), and 13.16 (8.93–14.62), respectively. These numbers also increased since 1990. The DALYs and deaths attributable to high BMI was 1.91 (0.95–3.11) and 0.07 (0.04–0.11), respectively. The thyroid cancer burden and high BMI attributed burden has increased from 1990 to 2019 in Iran. This study and similar studies’ results can be used for accurate resource allocation for efficient management and all potential risks’ modification for thyroid cancer with a cost-conscious view.

## Introduction

Thyroid cancer is the most prevalent endocrine cancer world-wide^1^. Globally, there were approximately 233,846 (95% Uncertainty Interval [UI]: 211,636–252,806) incident cases of thyroid cancer and 45,575 (41,289–48,775) deaths among all-ages in 2019. Thyroid cancer was accounted for approximately 1,231,841 (1,113,585–1,327,064) Disability-Adjusted Life Years (DALYs) in the same year^2^. Since 1990, thyroid cancer incidence is the most rapidly rising incidence among all cancers. The overall change in incidence of this cancer due to the age structure, population growth, and the incidence rate is 99% from 2005 to 2015^3^. Moreover, thyroid cancer deaths and DALYs were also increased by 63.75% and 88.79%, respectively, among all-ages and both sexes^2^. Therefore, assessing the burden of thyroid cancer at national and subnational levels as well as global level could assist in appropriate policy makings and identifying new priorities in resource allocation.

Plus, there are controversies in diagnosis and treatment of thyroid cancer in the world^4^ and These controversies might lead to over-managing^5,6^. Plus, thyroid cancer treatments include invasive approaches that are not devoid of side effects^6^. Hence, assessing the burden of thyroid cancer is not only a matter of resource allocation but also it might help in reducing side effects of over-managements by providing the big picture to the clinicians.

According to the previous studies, Iran follows a similar trends in thyroid cancer incidence, deaths, and DALYs^7,8^. However, the increase in the incidence could be a result of population growth and aging as well as increased age-specific incidence rate^9^. Hence, there is always a requirement for an updated, precise, and detailed data at national and sub-national levels to explore the pattern of thyroid cancer burden across a country for making timely and correct decisions.

It is also essential to identify associated risk factors to the thyroid cancer and their relative importance in the disease burden. Quantifying and tracking the prevalence of a risk factor could help in the risk modification in various locations. This objective could be accomplished by measuring the risk attributable DALYs^10^. The suggested risk factors by the literature are iodine intake, nitrate contamination, radiation exposure, vitamin D deficiency, and high BMI^11–17^. However, according to the Global Burden of Disease (GBD) study, high Body Mass Index (BMI) is the only established risk factor for thyroid cancer^18,19^. It has also been established that this risk factor has an increasing pattern in Iran^20^.

To the best of our knowledge, this is the first report of descriptive epidemiology of thyroid cancer in Iran at national and subnational levels. This study presents the thyroid cancer incidence, prevalence, deaths, DALYs, Years of Life Lost (YLLs), Years Lived with Disability (YLDs), and deaths, DALYs, YLLs, and YLDs attributed to the high Body Mass Index (BMI) in Iran and among its provinces from 1990 to 2019. The aforementioned indices are presented for both sexes, different age groups, and Socio-demographic Index (SDI) levels across the country.

## Materials and methods

### Overview

In this paper, thyroid cancer incidence, prevalence, deaths, DALYs, YLLs, and YLDs are reported using the Global Burden of Disease (GBD) 2019 study estimates in Iran at national and sub-national levels in 31 provinces from 1990 to 2019. These indices are also calculated in age-standardized rates except in decomposition analysis (that are crude rates) and reported as rates per 100,000 in the population. Moreover, the risks attributable DALYs, Deaths, YLLs, and YLDs to high BMI are presented across the country. Mortality to Incidence Ratio (MIR) was also calculated for each province among males, females and both sexes through dividing Age-Standardized Death Rate (ASDR) by Age-Standardized Incidence Rate (ASIR). Data analysis, tables, and demonstrations were conducted using R statistical packages v3.4.3 (http://www.r-project.org, RRID: SCR_001905) and presented with 95% Uncertainty Intervals (UI).

### Data source

GBD 2019 study^2^ estimated incidence, prevalence, mortality, DALYs, YLLs, and YLDs of 396 disease and injuries for 23 age groups; males, females, and both sexes combined; and 204 countries and territories that were grouped into 21 regions and seven super-regions, and sub-national data of some countries including Iran. GBD 2019 study is in agreement with the Guidelines for Accurate and Transparent Health Estimates Reporting (GATHER) statement and relies on various data sources for each disease. After that, data is processed and revised to correct biases and modeled to generate the estimates^2^. More data regarding to the results of this study is available from the GBD Results Tool^21^.

### Decomposition analysis

Three factors contribute to the change in thyroid cancer incidence from 1990 to 2019. The change results from population growth, population aging, and age-specific increase in incident cases. However, these factors contribute to the change of incidence to different extents. To study the extent of contribution of each factor following steps were taken. First, the age structure of the population and age-specific incidence of thyroid cancer rates in 1990 were applied to the population size of 2019. Second, the age-specific incidence rates of thyroid cancer in 1990 were applied to the age structure and population size in 2019. The difference between the numbers of incident cases in these two steps is attributable to the changes in age structure from 1990 to 2019. On the other hand, the difference between the first step and the actual incident cases in 1990 is attributable to the population growth. Finally, the difference between incident cases of the second step and the actual incidence in 2019 is attributable to the changes in age-specific incidence rates^9^.

### Attributable risk estimation

Since risk modification is a strategy toward preventing a disease, it is crucial yet challenging to track the burden attributable to the pertinent risk factors. Accordingly, the high BMI is the only risk factor reported for thyroid cancer by the GBD study after the causal evidence met the World Cancer Research Fund International (WCRF) grades of convincing evidence^18,19^. Hence, DALYs, deaths, YLLs, and YLDs attributable to the high BMI in Iran are reported in this study. The details of the calculation of the risk attribution are discussed elsewhere^10^. In brief, after determining the risk-outcome pair, the distribution of exposure for the risk by age, sex, location, and year was estimated. Then, the Population Attributable Fraction (PAF) and attributable-burden was estimated. For example, the risk attributable DALYs were estimated by multiplying DALYs by the population attributable fraction of the risk-outcome pair for a specific age, sex, location, and year.

$$Risk\;Attributable_{DALYs,\;deaths,\;YLLs,\;or\;YLDs} \, = \,PAF_{age,\;sex,\;location,\;and\;year} \, \times \,DALYs,\;Deaths,\;YLLs,\;or\;YLDs$$

### SDI levels

The SDI reflects socio-demographic development in a location. Three indices contribute to generating this index: average income per capita, average years of schooling among 15 years and older individuals, and the total fertility rate under the age of 25^22^. The first two are positively and the last one is negatively associated with SDI in a location. In this study, the SDI is categorized into five quintiles among Iran’s provinces: high SDI, high-middle SDI, middle SDI, low-middle SDI, and low SDI.

### Ethics

This study was approved by the institutional review board of Endocrinology and Metabolism Research Institute at Tehran University of Medical Sciences (IR.TUMS.EMRI.REC.1400.030).

This study’s results are based on estimates from the GBD 2019 study and in accordance with relevant guidelines and regulations. The authors also confirm that data used in the present study is publicly available.

## Results

### Thyroid cancer in Iran at national level

**Figure 1:**
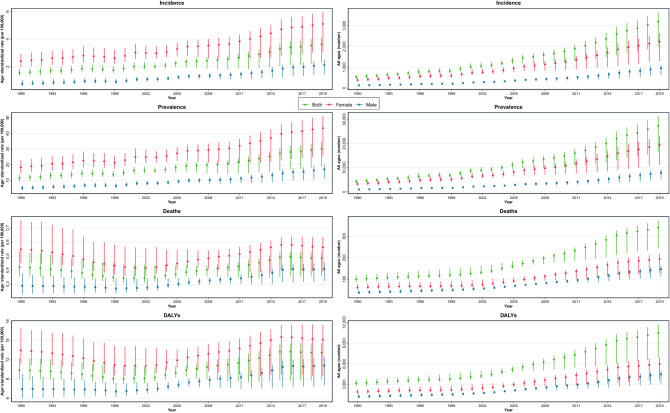
Time trend of the age-standardized and all-ages incidence, prevalence, deaths, and DALYs of thyroid cancer in Iran from 1990 to 2019 in by sex.

**Figure 2:**
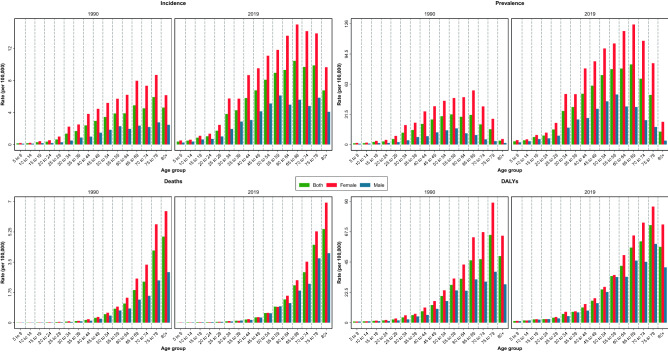
Thyroid cancer incidence, prevalence, deaths, and DALYs by age groups in 1990 and 2019 by sex.

The burden of thyroid cancer in Iran increased from 1990 to 2019 (Table 1 and Supplementary Table 1). The ASIR of thyroid cancer among both sexes was 1.57 (95% UI: 1.33–1.86) in 1990, 1.87 (1.64–2.17) in 2000, 2.54 (1.81–2.80) in 2010, and 3.63 (2.31–4.12) in 2019 per 100,000. This number increased 131% (53–191) from 1990 to 2019 and considering 10-year intervals, the highest change in ASIR of thyroid cancer was between 2010 and 2019 that was 43% (24–59) (Supplementary Table 2, Fig. 1). Disaggregated by sex, the age-standardized incidence rate in 2019 was 5.11 (3.08–5.96) and 2.15 (1.46–2.52) for females and males, respectively (Fig. 1). Among age groups for adults, the highest incident rate per 100,000 was in the 65 to 69 age group, whereas the lowest incident rate was in the 20 to 24 age group (i.e., 10.47 versus 1.02) (Fig. 2). The decomposition analysis of thyroid cancer in the country revealed that 342% of the total 493% increase in the all age’s incident cases between 1990 and 2019 was attributed to the increase in the age-specific incidence rate (Supplementary Table 3), whereas 107% and 44% of the total increase rate were attributable to the population aging and population growth, respectively.

**Table 1:** Burden measures by all ages number and age-standardized rate (per 100,000) by sex in 1990 and 2019 with percent change (%) between 1990 and 2019, Iran.

Measure	Metric	Year						% Change (1990 to 2019)		
		1990			2019					
		Both	Female	Male	Both	Female	Male	Both	Female	Male
Incidence	All ages number	539 (450 to 639)	400 (318 to 496)	139 (110 to 183)	3198 (1999 to 3649)	2242 (1310 to 2643)	956 (634 to 1129)	493.1 (279.1 to 667.7)	460.1 (237.1 to 681.2)	588.5 (340.8 to 829.0)
	Age-standardized rate (per 100,000)	1.57 (1.33 to 1.86)	2.41 (1.96 to 2.93)	0.78 (0.62 to 1.03)	3.63 (2.31 to 4.12)	5.11 (3.08 to 5.96)	2.15 (1.46 to 2.52)	130.7 (53.1 to 191.5)	112.1 (35 to 186.8)	176 (77.3 to 273)
Prevalence	All ages number	4317 (3532 to 5222)	3303 (2560 to 4172)	1015 (788 to 1344)	27,337 (16,709 to 31,333)	19,462 (11,236 to 23,044)	7875 (5156 to 9410)	533.2 (302.5 to 737.3)	489.3 (249.1 to 741.5)	676.1 (399.4 to 974.6)
	Age-standardized rate (per 100,000)	11.44 (9.38 to 13.85)	18.36 (14.38 to 23.03)	5.04 (3.87 to 6.74)	30.19 (18.75 to 34.55)	43.43 (25.73 to 51.01)	17.03 (11.25 to 20.26)	164.0 (76.6 to 245.9)	136.6 (49.2 to 228.9)	238.0 (118.5 to 371.7)
Deaths	All ages number	99 (86 to 118)	62 (51 to 79)	38 (30 to 50)	341 (245 to 375)	195 (131 to 220)	147 (112 to 163)	243.5 (138.5 to 327.8)	216.6 (104.3 to 305.5)	287.3 (155.0 to 405.5)
	Age-standardized rate (per 100,000)	0.42 (0.36 to 0.53)	0.55 (0.45 to 0.76)	0.29 (0.23 to 0.38)	0.49 (0.36 to 0.53)	0.56 (0.39 to 0.64)	0.41 (0.32 to 0.45)	15.1 (− 19.6 to 44.8)	2.4 (− 34.2 to 32.9)	41.8 (− 6.0 to 86.1)
DALYs	All ages number	3172 (2747 to 3697)	1934 (1630 to 2311)	1239 (1013 to 1598)	10,469 (6928 to 11,742)	5995 (3638 to 6924)	4474 (3132 to 5029)	230 (109.7 to 311.3)	210 (86.2 to 300.9)	261.2 (133.9 to 367.7)
	Age-standardized rate (per 100,000)	10.41 (8.96 to 12.29)	13.48 (11.31 to 17.06)	7.43 (6.00 to 9.77)	13.16 (8.93 to 14.62)	15.19 (9.52 to 17.42)	11.12 (7.97 to 12.44)	26.4 (− 16.4 to 57.7)	12.7 (− 30.3 to 43.4)	49.6 (− 2.9 to 95.6)
YLLs	All ages number	2909 (2532 to 3371)	1738 (1461 to 2103)	1171 (958 to 1521)	8870 (5960 to 9790)	4881 (3036 to 5533)	3989 (2831 to 4441)	204.9 (95.3 to 281.7)	180.8 (71.7 to 263.5)	240.7 (119.7 to 342.7)
	Age-standardized rate (per 100,000)	9.67 (8.33 to 11.48)	12.34 (10.34 to 15.61)	7.07 (5.69 to 9.27)	11.36 (7.8 to 12.45)	12.66 (8.15 to 14.31)	10.04 (7.33 to 11.17)	17.4 (− 22.5 to 46.4)	2.6 (− 36.6 to 31.7)	42.0 (− 8.1 to 85.4)
YLDs	All ages number	263 (172 to 377)	196 (125 to 286)	68 (43 to 100)	1600 (884 to 2322)	1115 (579 to 1632)	485 (288 to 707)	507.3 (284.0 to 693.3)	469.1 (238.5 to 707.8)	618.0 (360.0 to 900.5)
	Age-standardized rate (per 100,000)	0.74 (0.49 to 1.05)	1.14 (0.74 to 1.68)	0.36 (0.23 to 0.52)	1.80 (1.00 to 2.60)	2.53 (1.34 to 3.67)	1.08 (0.65 to 1.55)	144.8 (61.9 to 217.1)	122.0 (39.2 to 206.0)	200.6 (91.5 to 316.6)

Data in parentheses are 95% Uncertainty Intervals (95% UIs).

*DALYs* disability-adjusted life years, *YLLs* years of life lost, *YLDs* years lived with disability.

The ASPR of thyroid cancer per 100,000 for both sexes was 30.19 (18.75–34.55) in 2019 which increased 164% (77–246) from 11.44 (9.38–13.85) in 1990. Females had a higher number of prevalent cases than males and the highest ASPR was in 65 to 69 age group in both sexes (Fig. 2). The ASDR of thyroid cancer was 0.49 (0.36–0.53) per 100,000 in 2019 and 0.42 (0.36–0.53) in 1990 (Table 1, Fig. 1). Females ASDR in 2019 was 0.56 (0.39–0.64) and males ASDR was 0.41 (0.32–0.45). The highest death rate was observed in the 80+ age group in both sexes (Fig. 2). In addition, thyroid cancer accounted for 13.16 (8.93–14.62) age-standardized DALYs rate per 100,000 in 2019 (Table 1, Fig. 1), of which 1.92 (0.96–3.11) is attributable to the high BMI. Furthermore, 0.07 (0.04–0.11) of the death rates were attributed to high BMI in this year. The detailed data on the burden of thyroid cancer and “its attributable risk factor” are presented in Tables 1 and 2, respectively.

**Table 2:** Attributed burden measures to high BMI by all ages number and age-standardized rate (per 100,000) by sex in 1990 and 2019 with percent change (%) between 1990 and 2019, Iran.

Measure	Metric	Year						% Change (1990 to 2019)		
		1990			2019					
		Both	Female	Male	Both	Female	Male	Both	Female	Male
Deaths	All ages number	9 (5 to 16)	6 (3 to 10)	4 (1 to 8)	50 (25 to 81)	26 (14 to 41)	24 (7 to 47)	434.6 (257.7 to 631.4)	342.4 (184.5 to 509.4)	584.2 (326.4 to 931.7)
	Age-standardized rate (per 100,000)	0.04 (0.02 to 0.07)	0.05 (0.02 to 0.09)	0.03 (0.01 to 0.06)	0.07 (0.04 to 0.11)	0.07 (0.04 to 0.12)	0.07 (0.02 to 0.13)	83.9 (23.2 to 158.9)	47.7 (− 4.9 to 107.6)	150.8 (55.4 to 282.6)
DALYs	All ages number	289 (142 to 499)	178 (92 to 303)	111 (31 to 247)	1545 (756 to 2523)	789 (409 to 1288)	756 (218 to 1431)	435.1 (234.9 to 638.6)	344.1 (171.6 to 513.9)	580.7 (314.8 to 934.9)
	Age-standardized rate (per 100,000)	0.98 (0.49 to 1.68)	1.27 (0.65 to 2.17)	0.70 (0.20 to 1.56)	1.92 (0.96 to 3.11)	1.98 (1.05 to 3.23)	1.85 (0.54 to 3.52)	96.3 (25.5 to 171.1)	55.8 (− 2.9 to 113.9)	164.5 (61.1 to 302.5)
YLLs	All ages number	265 (131 to 459)	160 (82 to 271)	105 (29 to 236)	1316 (641 to 2151)	642 (342 to 1041)	674 (195 to 1277)	396.4 (211 to 586.3)	301.5 (148.3 to 456.7)	540.9 (290 to 878)
	Age-standardized rate (per 100,000)	0.91 (0.45 to 1.57)	1.16 (0.59 to 1.97)	0.66 (0.18 to 1.48)	1.66 (0.83 to 2.71)	1.65 (0.88 to 2.65)	1.67 (0.48 to 3.17)	83.3 (17.3 to 155)	42.1 (− 11 to 95.7)	151 (52.7 to 283.4)
YLDs	All ages number	24 (11 to 43)	18 (8 to 32)	6 (2 to 14)	229 (104 to 407)	147 (63 to 260)	83 n	867.8 (525.5 to 1280.8)	729.3 (405.3 to 1143.9)	1274.7 (742.4 to 2144.1)
	Age-standardized rate (per 100,000)	0.07 (0.03 to 0.13)	0.11 (0.05 to 0.20)	0.03 (0.01 to 0.08)	0.26 (0.12 to 0.45)	0.33 (0.14 to 0.58)	0.18 (0.05 to 0.36)	262.6 (140.6 to 404.4)	201.4 (92.2 to 342.8)	426.9 (222.2 to 750.3)

Data in parentheses are 95% Uncertainty Intervals (95% UIs).

*DALYs* disability-adjusted life years, *YLLs* years of life lost, *YLDs* years lived with disability.

### Thyroid cancer in Iran at the province level

#### Thyroid cancer incidence

**Figure 3:**
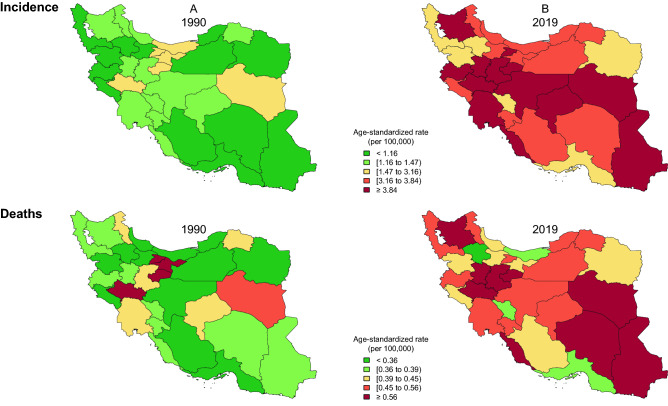
The age-standardized incidence and deaths of thyroid cancer in provinces of Iran (**A**: 1990, **B**: 2019), Republished from https://www.openstreetmap.org/ under a CC BY license, with permission from https://www.openstreetmap.org/copyright, original copyright 2022.

**Figure 4:**
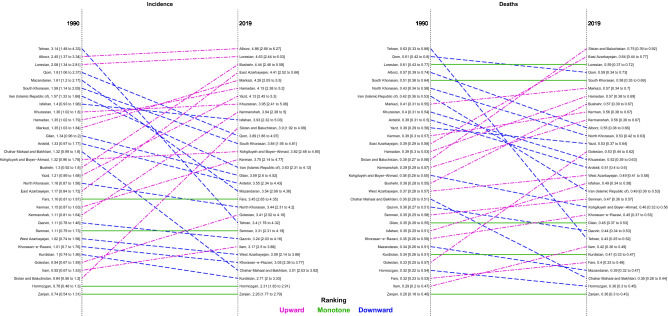
The changes in the thyroid cancer age-standardized incidence and deaths from 1990 to 2019 in both sexes in provinces of Iran: red arrow indicates upward ranking, blue arrow indicates downward ranking, and green arrow indicates monotone ranking.

The ASIR of thyroid cancer varied across Iran from 1990 to 2019 (Fig. 3). Indeed, it ranged from 2.26 (1.77–2.79) (i.e. in Zanjan) to 4.86 (2.66–6.27) (i.e. in Alborz) among provinces in 2019 (Supplementary Table 1). Even though the ASIR increased in all provinces in this interval, the difference between the province with highest ASIR and the province with lowest ASIR was lower in 1990 (i.e. 2.40 versus 2.60). However, the extent of change varied among provinces from 8% (− 24 to 55) to 362% (85–652) (Supplementary Table 1, Fig. 4, and Supplementary Fig. 1). Although the significance of differences is discussable in some provinces, in both years, females had higher ASIR than males in all provinces. However, in 2019, all provinces had lower female to male ratio except two (i.e. Bushehr and Kerman) compared to 1990. The highest and the lowest female to male ratio in 1990 was 4.24 and 1.98, whereas in 2019, it was 4.02 and 1.33 respectively (Supplementary Table 1).

The decomposition analysis of thyroid cancer at the province level showed that in all provinces except one (i.e. Tehran), the largest proportion of all-ages increase in both sex incidence cases was attributable to the increase in the age-specific incidence rate. In contrast, in Tehran, the largest proportion was attributed to population aging (Supplementary Table 3). Among provinces, Sistan and Baluchistan (1156.7%) had the highest increase attributable to the increase in age-specific incidence rate, Alborz (180.8%) had the highest increase attributable to the population aging, and Hormozgan (105%) had the highest increase attributable to the population growth.

#### Thyroid cancer prevalence

The ASPR of thyroid cancer also varied among provinces of Iran from 1990 to 2019. In 2019, it ranged from 18.42 (13.94–23.16) in Zanjan to 41.20 (21.75–53.68) in Alborz (Supplementary Table 1). The ASPR also increased in all provinces between these years, but the difference between the first and the last rank of ASPR among provinces was lower in 1990 (i.e. 19.94 versus 22.78). Although ASPR increased in all provinces, the extent of change varied from 15% (− 21 to 69) to 537% (146–1061) (Supplementary Table 1 and Supplementary Fig. 1). Females had higher ASPR than males in all provinces in both years. However, the uncertainty intervals of the ASPRs for females and males overlapped in some provinces. In 2019, all provinces had lower female to male ratio except one (i.e. Bushehr) compared to 1990, and the highest and lowest female to male ratio was 4.45 and 1.42. However, in 1990, it was 4.74 and 2.50 respectively (Supplementary Table 1).

#### Thyroid cancer deaths

Although there were minimal disparities, thyroid cancer causes relatively low death rates among the provinces of Iran (Fig. 3). In 2019, ASDR ranged from 0.36 (0.30–0.43) (i.e. in Zanjan) to 0.75 (0.39–0.92) (i.e. in Sistan and Baluchistan) (Supplementary Table 1). Despite the fact that ASDR increased in most provinces from 1990 to 2019, the difference between the first and the last rank of ASPR was lower in 1990 (i.e. 0.37 versus 0.39). Among the provinces in which ASDR increased between these years, the extent of change ranged from 12% (− 27 to 67) to 92% (− 11 to 191). However, the ASDR decreased in four provinces from − 0.2 (− 31.9 to 42.1) in Chahar Mahaal and Bakhtiari to − 32% (− 52 to − 6) in Tehran (Supplementary Table 1 and Supplementary Fig. 1). In 1990, females had higher ASDR than males in all provinces, whereas in 2019, males had higher ASDR in five provinces. However, the significance of these differences has to be further evaluated in most of the provinces. However, in 2019, all provinces had lower female to male ratio compared to 1990. Furthermore, the highest and lowest female to male ratio in 1990 was 2.75 and 1.34, and in 2019, it was 2.05 and 0.80 respectively (Supplementary Table 1). Moreover, the age-standardized Mortality to Incidence Ratio (MIR) was higher in males in all of the provinces from 1990 to 2019 (Supplementary Fig. 2).

#### Thyroid cancer DALYs

In the case of thyroid cancer DALYs among provinces of Iran, disparities were observable. Indeed, in 2019, it varied from 9.17 (7.62–10.92) in Zanjan to 21.30 (10.37–26.61) in Sistan and Baluchistan (Supplementary Table 1). Although, the age-standardized DALY rate increased in almost all provinces from 1990 to 2019, the difference between the province with the highest and the lowest rank was lower in 1990 (i.e. 9.69 versus 12.13). The age-standardized DALY rate was only decreased in Tehran [− 28% (95% UI: − 47 to − 2)]. Among the rest of provinces, the extent of change varied among provinces from 3% (− 28 to 54) to 122% (− 4 to 252) (Supplementary Table 1, and Supplementary Fig. 1). In 1990, females had higher age-standardized DALYs than males in all provinces. However, in 2019, in five provinces males had higher age-standardized DALYs. Similar to previous indices, the significance of the differences between females and males are discussable. In 2019, all provinces had lower female to male ratio compared to 1990. Moreover, the highest and the lowest female to male ratio in 1990 was 2.61 and 1.26, whereas in 2019, it was 2.10 and 0.78 respectively (Supplementary Table 1).

#### Thyroid cancer risk attributable DALYs

The high BMI is the only risk factor attributable to thyroid cancer introduced by the GBD studies. Here, the risk-attributable burden at national level will be reported at the Table 2, and the risk attributed burden at province level are presented in Supplementary Table 4.

**Figure 5:**
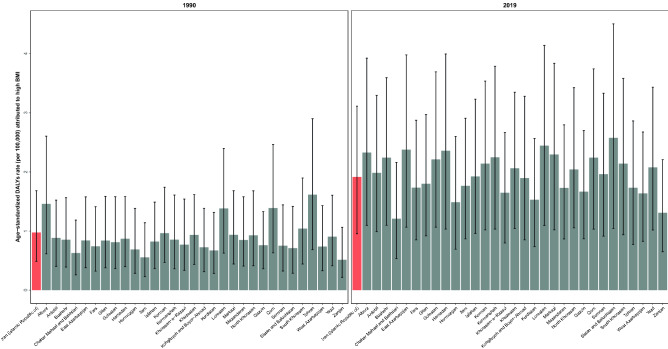
The thyroid cancer age-standardized DALYs attributed to high BMI in 1990 and 2019 in both sexes in provinces of Iran.

The risk attributable DALY in 2019 varied from 1.21 (0.53–2.16) in Chahar Mahaal and Bakhtiari to 2.58 (1.04–4.50) in Sistan and Baluchistan (Fig. 5). The risk attributable DALY increased in all of the provinces from 1990 to 2019. However, the difference between the first and the last rank was lower in 1990 (i.e. 1.11 versus 1.37). Besides, the extent of change in this index ranged from 7% (− 24 to 57) to 262% (53–536). In 1990, females had higher risk attributable DALYs than males in all provinces, whereas in 2019, males had higher risk attributable DALYs in half of the provinces. However, the uncertainty intervals overlapped in all of the provinces in both years. In 2019, all provinces had lower female to male ratio compared to 1990. In addition, the highest and lowest female to male ratio in 1990 was 2.64 and 1.29, and in 2019, it was 1.61 and 0.62 respectively.

### Thyroid cancer in SDI levels of Iran

**Figure 6:**
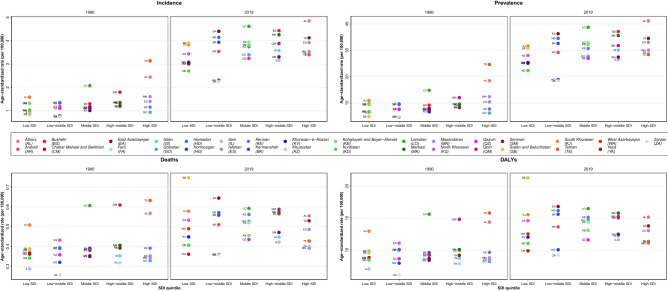
The thyroid cancer age-standardized incidence, prevalence, deaths, and DALYs in provinces of Iran in 1990 and 2019 by SDI levels.

The overall age-standardized rate of incidence, prevalence, deaths, and DALYs of thyroid cancer increased from 1990 to 2019 in almost all provinces in Iran from all five SDI levels (Fig. 6). However, in 2019, similar ASIRs, ASPRs, ASDRs, and DALYs were observed in provinces with different SDI levels. For example, in 2019, Isfahan [3.93 (2.32–5.03)] as a high SDI province and Sistan and Baluchistan [3.9 (1.92–4.99)] as a low SDI province were close together in case of ASIR of thyroid cancer. In contrast, East Azarbayejan [4.41 (2.52–5.66)] and Zanjan [2.25 (1.77–2.79)] with similar SDI levels were far from each other in the spectrum. However, in 1990, the SDI level was more associated with the thyroid cancer ASIR, and it was higher in higher SDI levels.

## Discussion

In this study, we have shown that although variability existed among provinces, the general burden of thyroid cancer increased from 1990 to 2019 in Iran. Moreover, the difference between provinces was higher, and female to male ratios were lower in 2019 comparing to 1990. The high BMI attributable DALYs also increased between these two years. As a matter of fact, based on the uncertainty intervals, the ASIR and the ASPR of thyroid cancer was higher in 2019 in Iran, whereas the ASDR, DALY, and the high BMI attributable DALY did not seem to be significantly different between these two years. This study’s results also suggest that SDI in a province is not significantly associated with the thyroid cancer burden in 2019. Indeed, some provinces with different SDIs had similar ASIRs. Hence, SDI had no effect on Thyroid cancer epidemiologic measures in Iran in 2019.

It is worth noting that the observed ranges of disparity in all of the indices among the provinces had overlapping uncertainty intervals. The implication of this finding is that there might not be significant differences in the burden of thyroid cancer among provinces, which needs to be evaluated in future studies.

One explanation in support of the disparities in the incidence rate among different provinces might be differences in the healthcare access and quality. The more aggressively a nodule is approached due to higher healthcare access, the more will the case finding be. In addition, it is believed that one of the factors affecting the increasing incidence and prevalence of thyroid cancer in Iran is the increasing number of skilled endocrinologists and radiologists^8^. Hence, the sub-clinical disease is more likely to be diagnosed^23–25^. However, since the availability of the diagnostic modalities might be affected by the SDI in a province^26–28^, an increase in the thyroid cancer ASIR in low SDI provinces, including Sistan and Baluchistan might not be solely due to the increased diagnostics. Besides, the decrease in the ASDR in four provinces, including Tehran and other two provinces nearby Tehran from 1990 to 2019, might be reflecting the inequities in the treatment of thyroid cancer across the country due to inequities in healthcare quality, possibly in favor of the capital. Nevertheless, based on the uncertainty intervals, the change from 1990 to 2019 might only be significant in Sistan and Baluchistan and only in the ASIR index. This province is a relatively deprived area in Iran and the increase in the ASIR in the recent years might be mostly due to the better diagnostics. Furthermore, the age structure of a population affects the incidence, prevalence, deaths, and DALYs of a disease. Specially, since Iran is a country in the Eastern Mediterranean Region of the WHO with a dynamic age composition^29^, the effect of the population age structure is strong in the findings of this study.

It is also worth noting that the observed trend of the rising thyroid cancer incidence was more significant between the years 1990 and 2010 compared to the years between 2010 and 2019. Although the duration between 1990 and 2010 was longer, this difference between these two time intervals might be due to the revision of the American Thyroid Association’s guidelines on thyroid cancer diagnosis and treatment and other countries developing similar guidelines in 2010^30^. However, according to the GBD studies’ data source, there are limited number of data sources for thyroid cancer burden in Iran after 2010^31^. Hence, most of the data between 2010 and 2019 is based on estimations. Therefore, more precise data needs to be acquired from future field studies.

The observed pattern and the increasing trend of thyroid cancer incidence might result from various factors. The improvement of medical techniques has occurred in diagnostics and it might have resulted in a higher rate of diagnosis of subtle malignancies^32^. Accordingly, the diagnosis of subtle malignancies leads to overdiagnosis which is estimated to account for over 80% of female thyroid cancer in countries including South Korea, Belarus, China, Italy, Croatia, Slovakia, and France between 2008 and 2012^33^. Similarly, in Spain, the increased incidence of thyroid cancer is mostly attributed to the higher diagnosis rate^34^. However, in comparison with global rates, the increase in the incidence rate of thyroid cancer in Iran was relatively lower^35^. This finding might be explained by a relatively lower rate of overdiagnosis in Iran. In fact, although the increased ASIR and DALYs in other countries, including South Korea^36^ were mostly due to the higher diagnosis rates by cancer screening programs, this scenario does not apply to Iran because currently there are no thyroid cancer screening programs in the country. Since there are various burdens of thyroid cancer in the world, it might be a matter of the cost-effectiveness of thyroid cancer screening programs in different regions^37,38^. Even in South Korea, the necessity of these screenings remains controversial^39^. Thus, the cost-effectiveness of thyroid cancer screening programs in all countries including Iran needs future studies to be evaluated. Nevertheless, the role of increased diagnosis must not overshadow the role of other potential risk factors of thyroid cancer. Besides, advanced diagnostics are expensive and therefore, the increasing burden must not be solely attributed to overdiagnosis^40^. For example, in China, the exposure to endocrine-disrupting chemicals such as polychlorinated biphenyls (PCBs), asbestos, pesticides, and polybrominated diphenyl ethers (PBDEs) have been suspected to play role in the increasing incidence^41,42^. Another study in the Czech Republic also reported the role of other factors such as chemical exposures and iodine deficiency in the increasing burden of thyroid cancer^43^. Previously in Iran, radiation exposure and underlying benign thyroid disease have been found to be associated with the thyroid cancer incidence^44,45^. Further studies are required to assess the role of these factors more accurately in the increasing burden of thyroid cancer in Iran.

It is worth noting that despite the increasing incidence rates, the lower difference in death rates between 1990 and 2019 might be due to better clinical management of thyroid cancer in recent years^42^. The evidence in support of this assertion is that although the ASIR of thyroid cancer increased from 1990 to 2019, the age-standardized MIR has decreased in all provinces (Supplementary Fig. 1). However, even though Iranian health care system have been evidently successful to some extents, it is currently facing new challenges regarding the non-communicable disease and this may affect the performance of managing these disease including thyroid cancer^46^. Hence, more detailed analysis needs to be done in Iran at subnational level using indices measuring the quality of care^47^.

Finally, the increased burden attributable to the high BMI in Iran suggests that Iranian population is failing to manage this risk factor^48^. To explain the thyroid cancer burden disparities, disparities in other risk factors of thyroid cancer in different provinces needs to be assessed as well^11–16^. Accordingly, East Azarbayejan and Sistan and Baluchistan, which were among the provinces with the highest ASDRs and DALYs, had the lowest urinary iodine concentration in 2014^15^. Moreover, Sistan and Baluchistan had the highest increase in ASIR, ASPR, DALYs, and ASDRs of thyroid cancer. On the other hand, Kurdistan was among the provinces with the highest urinary iodine concentration and the lowest ASIR, ASPR, ASDR, and DALYs. However, these are descriptive interpretations and more precise analyses are needed to evaluate the significance conclude that the aforementioned risk factors are correlated with the findings of this study.

Although the significance of the differences were debatable, the variability was also observed in different sexes. In each year from 1990 to 2019, the female gender had more ASIRs and ASDRs. Even though the reason behind the higher burden among the females in thyroid cancer remains unknown, there are some possible explanations. One explanation is that there are identical hormonal signaling pathways playing role in thyroid and breast cancer, both of which having the highest incidence in women^49,50^. Another explanation is that the female gender is associated with higher usage of diagnostic medical imaging modalities, especially after the age of 45^51^. However, in 2019, female to male ratio was lower in most of the provinces in case of thyroid cancer burden. Indeed, the high BMI attributable DALY was higher in males in half of the provinces in 2019. Thus, it might be deductible that the high BMI in males is resulting in the convergence of the thyroid cancer burden in females and males in Iran.

However, in the case of deaths, the higher rates among women was mostly due to the higher incidence. Following this theory, the age-standardized mortality to incidence ratio was calculated in all provinces of the country. Generally, the age-standardized mortality to incidence ratio was higher in males in the provinces of Iran during all years from 1990 to 2019. However, the significance of this difference needs to be evaluated as well. Indeed, a previous study (i.e., in 2009) had indicated that the survival rate was not significantly different between the two genders in Iran^52^. However, this study’s finding was consistent with the previous studies in the U.S, indicating that the survival was lower in the male population^53^. The differences in the health care seeking attitudes among men and women are probably the explanation behind these findings^54^.

There were also disparities among different age groups in the thyroid cancer incidence rate. The highest incidence rate of thyroid cancer in 2019 among men was in the 55 to 59 age group, whereas among women, it was in the 65 to 69 age group. In contrast, in 1990, the highest incidence rate was in the 75 to 79 age group among men and women. This finding might be due to the earlier diagnosis of thyroid cancer in recent years. However, this result was in contrast with the findings of the studies in the U.S community^55^. The results also indicate that thyroid cancer rarely affects children under the age of 15 in Iran^8^.

This study’s results are in agreement with the World Health Organization warning against thyroid cancer in the world^56^. The 0.049% (0.044–0.053) of total DALYs in 2019 was due to thyroid cancer^57^, however, only 0.003% of the cancer research budget is allocated to thyroid cancer in the world^58^. Therefore, resource allocation for this cancer research, subsequent management, and improving healthcare quality might need to be revised across the globe. Plus, the world is failing in lifestyle improvement^10^, and consequently leading to the increased distribution of risk factors (e.g., high BMI). Thus, risk modification as a strategy in managing the thyroid cancer burden remains challenging. However, it has to be elucidated that what makes provinces with low burden of thyroid cancer (e.g. Zanjan) different from the others and modify the affecting factors in other provinces.

Similar to the other GBD studies, the limitations of this study are mostly due to the data availability^2^. Despite the fact that GBD study relies on modeling to construct estimates, sufficient primary data is not available from every location due to weak registry systems and this lack of data might challenge the accuracy of estimations. Accordingly, representing the UI around estimates remains challenging because of the sparse data. Therefore, despite improvements in precision of the estimation models, there is always a need for better and more primary data collection. Another limitation of this study was considering high BMI as the only risk factor for thyroid cancer. Finding more causal connections between risks and outcomes would result in better explanation of the observed patterns and spotting proper intervention points. To accomplish this objective, other studies including Mendelian randomization studies could be used in the meta-regression^10,59^. Furthermore, thyroid cancer is not limited to a single histology and different histological types have different risk factors and outcomes. Hence, disaggregation of the thyroid cancer burden estimation by histology would provide more comprehensive insights towards this cancer^60^.

On the other hand, providing sub-national data, decomposition analysis, analyzing the effect of SDI on thyroid cancer burden, calculating MIR and attributed burden to a risk factor are among the strengths of this article.

In conclusion, this study illustrates that the thyroid cancer burden and high BMI attributed burden has increased from 1990 to 2019 in Iran. This study and similar studies’ results would help in the health systems appropriate resource allocation for managing the thyroid cancer burden and reduce the disparities in this regard. However, this resource allocation must have a cost-conscious view to avoid overmanaging and must be able to enhance the awareness of the medical community about overdiagnosis. In addition, the exposure to risk factors of this cancer including high BMI and the other abovementioned factors need to be reduced as much as possible, especially among the high-risk groups.

## Supplementary Information

Supplementary Legends.

Supplementary Figure 1.

Supplementary Figure 2.

Supplementary Figure 3.

Supplementary Table 1.

Supplementary Table 2.

Supplementary Table 3.

Supplementary Table 4.

## Data Availability

The authors confirm that the datasets generated and/or analyzed during the current study are publicly available in the Global Health Data Exchange repository accessible from http://ghdx.healthdata.org/gbd-results-tool and https://vizhub.healthdata.org/gbd-compare/ with the GBD code of B.1.23. The GBD 2019 study was conducted by the Institute for Health Metrics and Evaluation (IHME). GBD 2019 study included 396 disease and injuries incidence, prevalence, mortality, DALYs, YLLs, and YLDs for 23 age groups; males, females, and both sexes combined; and 204 countries and territories that were grouped into 21 regions and seven super-regions. Furthermore, sub-national data of some countries including Iran is also available. Plus, the International Statistical Classification of Diseases and Related Health Problems 10th Revision, World Health Organization version (ICD-10) codes of morbidity and mortality (i.e. C73-C73.9, D09.3, D09.8, D34-D34.9, and D44.0) were used^2^.

## Abbreviations

ASDR: Age-standardized death rate
ASIR: Age-standardized incidence rate
ASPR: Age-standardized prevalence rate
DALYs: Disability-adjusted life years
GBD: Global burden of disease
MIR: Mortality to incidence ratio
SDI: Socio-demographic index
UI: Uncertainty interval
YLDs: Years lived with disability
YLLs: Years of life lost
