# Supplementary material for: National, sub-national, and risk-attributed burden of thyroid cancer in Iran from 1990 to 2019
Source: Sci Rep. 2022 Aug 2;12:13231. doi: 10.1038/s41598-022-17115-0 (PMC9346133; doi:10.1038/s41598-022-17115-0)
Supplement: Supplementary file 1 — Supplementary Legends. [file 41598_2022_17115_MOESM1_ESM.docx]

**Legends of Supplementary figures and tables**

**Supplementary Figure 1.** The percent of change of thyroid cancer age standardized incidence, prevalence, deaths, and DALYs of both sexes in provinces of Iran between 1990 and to 2010, and between 2010 and 2019

**Supplementary Figure 2.** Time trend of the age standardized mortality to incidence ratio (MIR) in both sexes, females, and males in provinces of Iran

**Supplementary Figure 3.** Geographical distribution of Iranian provinces

Republished from <https://www.openstreetmap.org/> under a CC BY license, with permission from <https://www.openstreetmap.org/copyright>, original copyright 2022.

**Supplementary Table 1.** Age-standardized rates of incidence, prevalence, deaths, DALYs, YLLs, and YLDs (per 100,000), by sex in 1990 and 2019 and percent change (%) from 1990 to 2019 at sub-national level

**Supplementary Table 2.** Percent of changes in age-standardized incidence, prevalence, deaths, DALYs, YLLs, and YLDs in Iran at national and subnational levels between 1990 and 2000, 2000 and 2010, and 2010 and 2019 by sex

**Supplementary Table 3.** Incidence trends based on decomposition analysis between 1990 and 2019 at national and sub-national levels by sex

**Supplementary Table 4.** Attributed age-standardized rates of deaths, DALYs, YLLs, and YLDs (per 100,000) to high body-mass index by sex in 1990 and 2019, and percent change (%) from 1990 to 2019 at sub-national level
