# Supplementary figures and images for: National, sub-national, and risk-attributed burden of thyroid cancer in Iran from 1990 to 2019
Source: Sci Rep. 2022 Aug 2;12:13231. doi: 10.1038/s41598-022-17115-0 (PMC9346133; doi:10.1038/s41598-022-17115-0)

Incidence

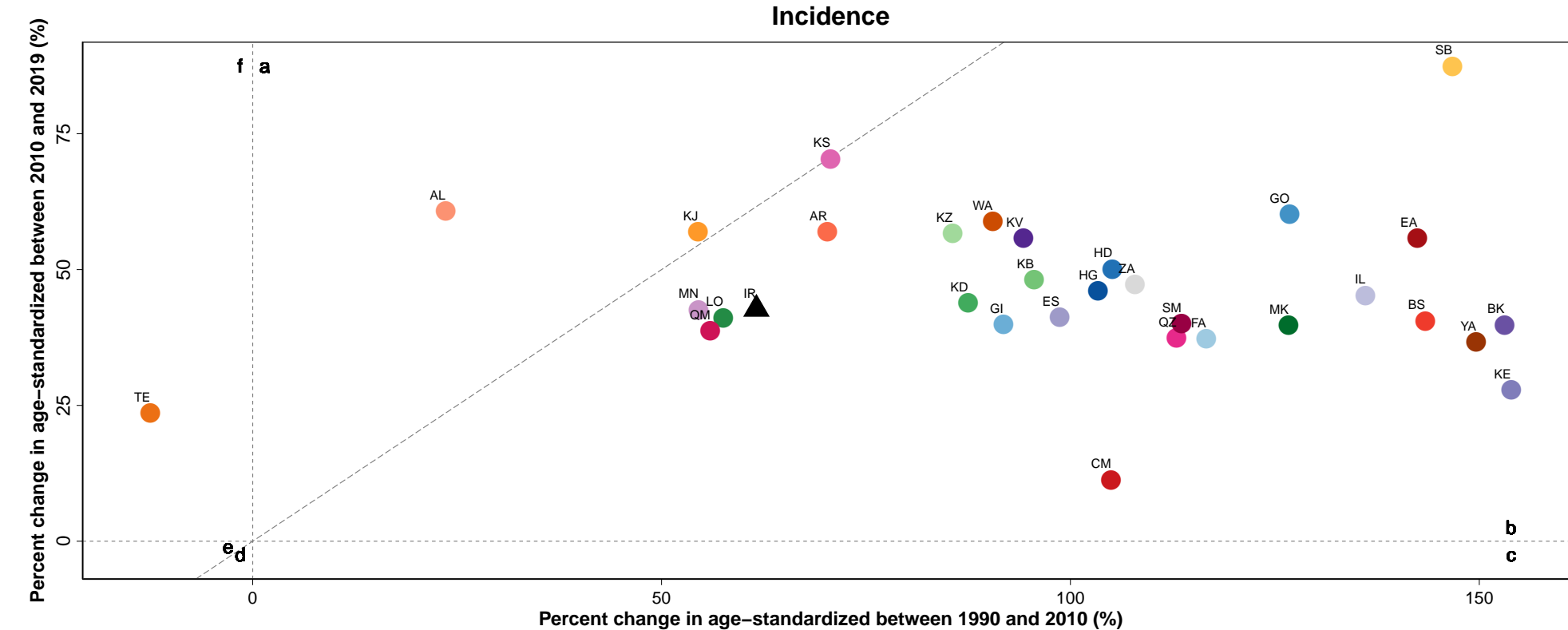

Prevalence

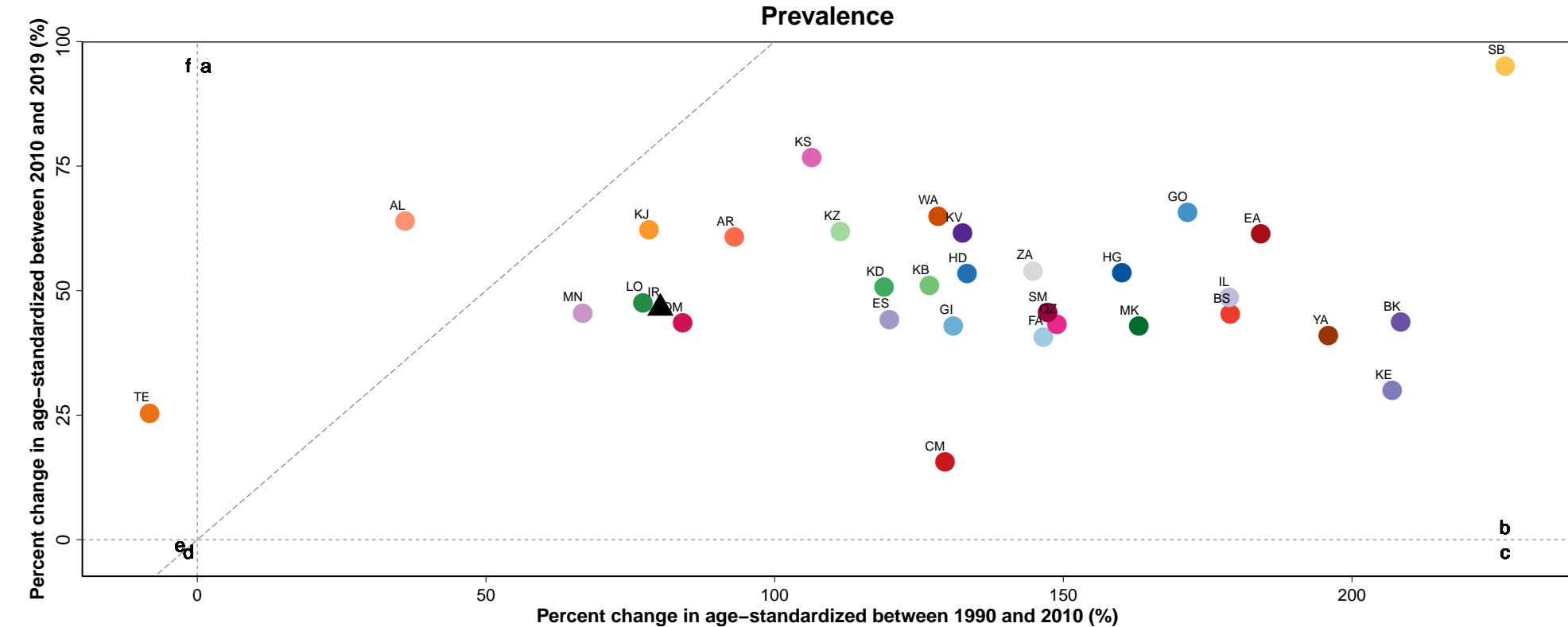

Deaths

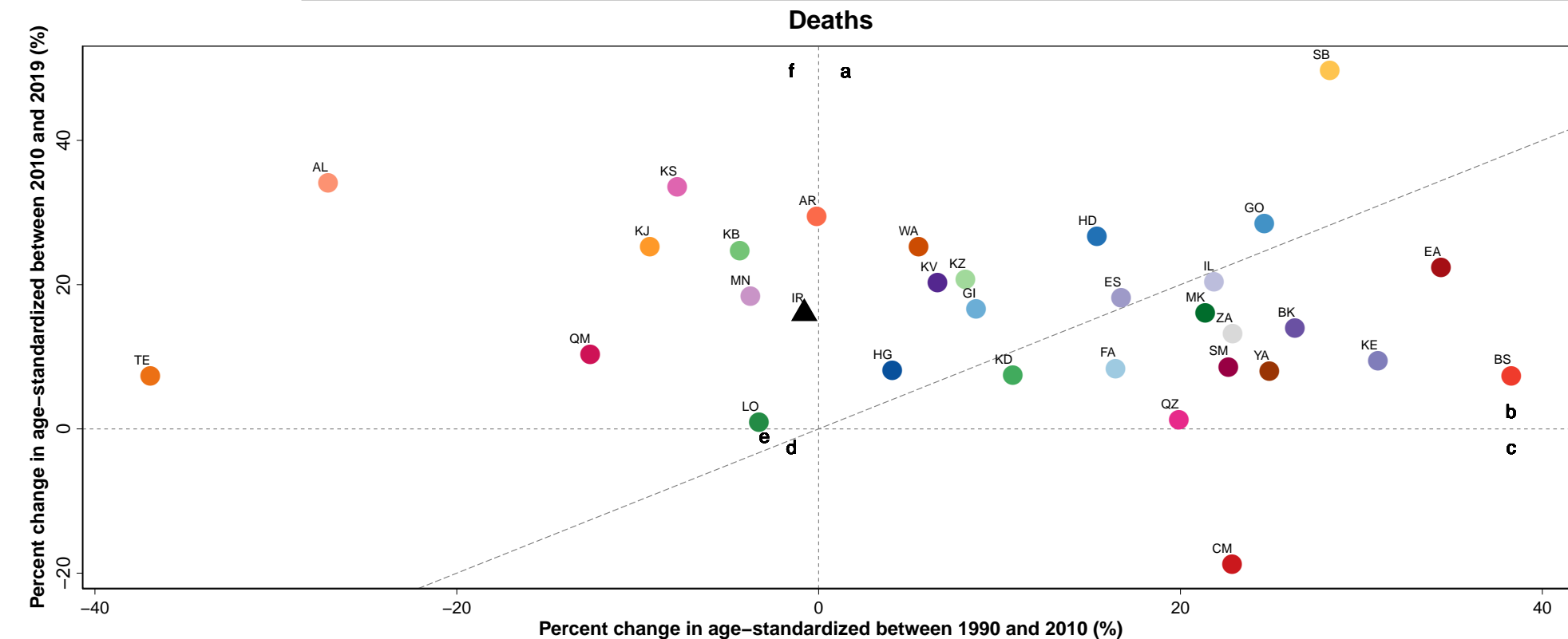

DALYs

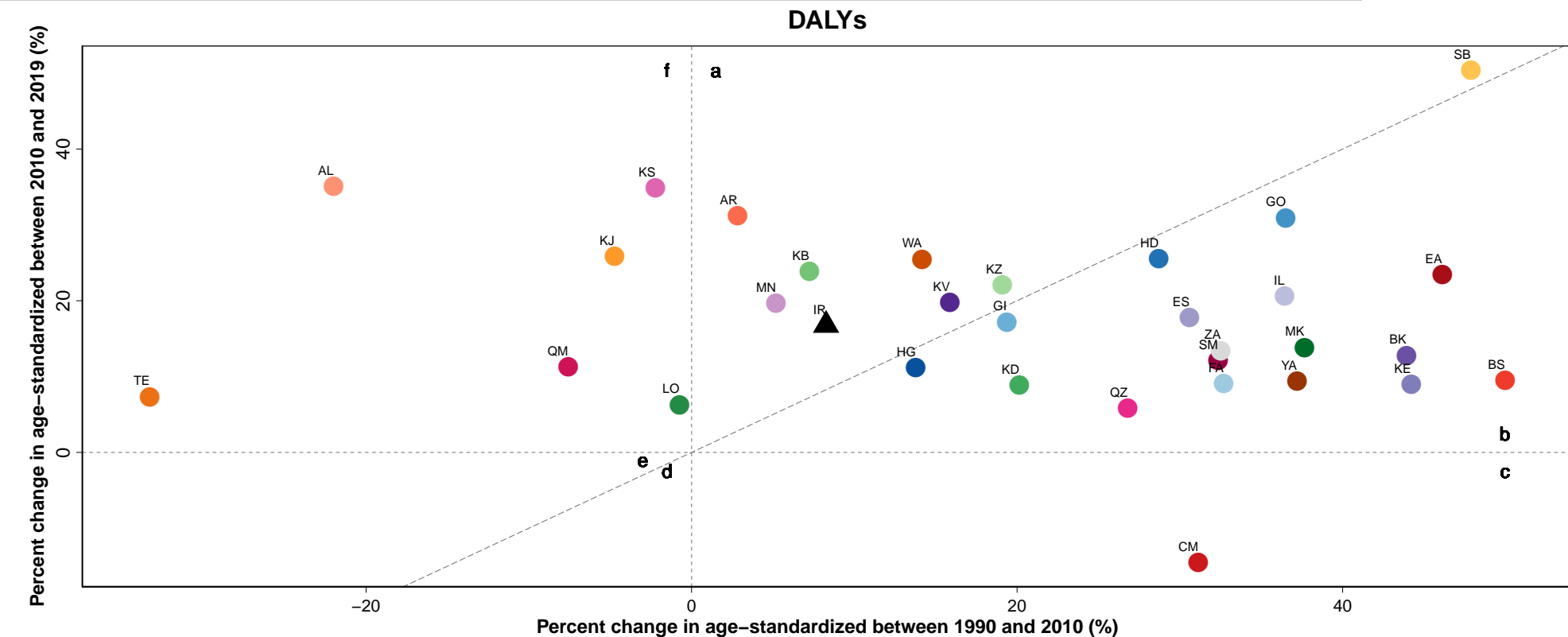

Supplement: Supplementary file 2 — Supplementary Figure 1. [file 41598_2022_17115_MOESM2_ESM.pdf]
