# Supplementary material for: National, sub-national, and risk-attributed burden of thyroid cancer in Iran from 1990 to 2019
Source: Sci Rep. 2022 Aug 2;12:13231. doi: 10.1038/s41598-022-17115-0 (PMC9346133; doi:10.1038/s41598-022-17115-0)
Supplement: Supplementary file 3 — Supplementary Figure 2. [file 41598_2022_17115_MOESM3_ESM.pdf]

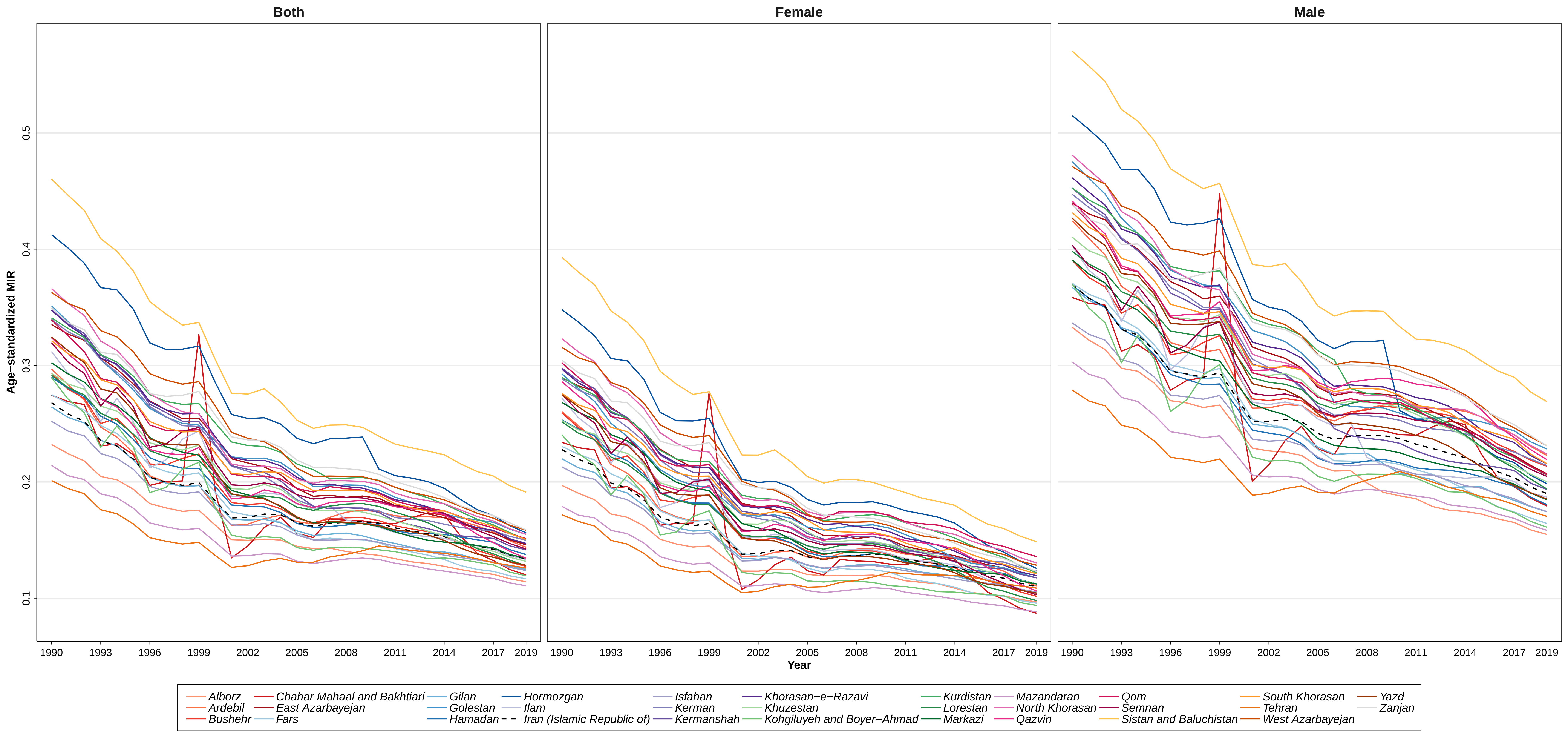
Both
Female
Male
Age−standardized MIR
Year
0.1
0.2
0.3
0.4
0.5
1990
1993
1996
1999
2002
2005
2008
2011
2014
2017
2019
Alborz
Ardebil
Bushehr
Chahar Mahaal and Bakhtiari
East Azarbayejan
Fars
Gilan
Golestan
Hamadan
Hormozgan
Ilam
Iran (Islamic Republic of)
Isfahan
Kerman
Kermanshah
Khorasan−e−Razavi
Khuzestan
Kohgiluyeh and Boyer−Ahmad
Kurdistan
Lorestan
Markazi
Mazandaran
North Khorasan
Qazvin
Qom
Semnan
Sistan and Baluchistan
South Khorasan
Tehran
West Azarbayejan
Yazd
Zanjan
