# Supplementary material for: National, sub-national, and risk-attributed burden of thyroid cancer in Iran from 1990 to 2019
Source: Sci Rep. 2022 Aug 2;12:13231. doi: 10.1038/s41598-022-17115-0 (PMC9346133; doi:10.1038/s41598-022-17115-0)
Supplement: Supplementary file 4 — Supplementary Figure 3. [file 41598_2022_17115_MOESM4_ESM.pdf]

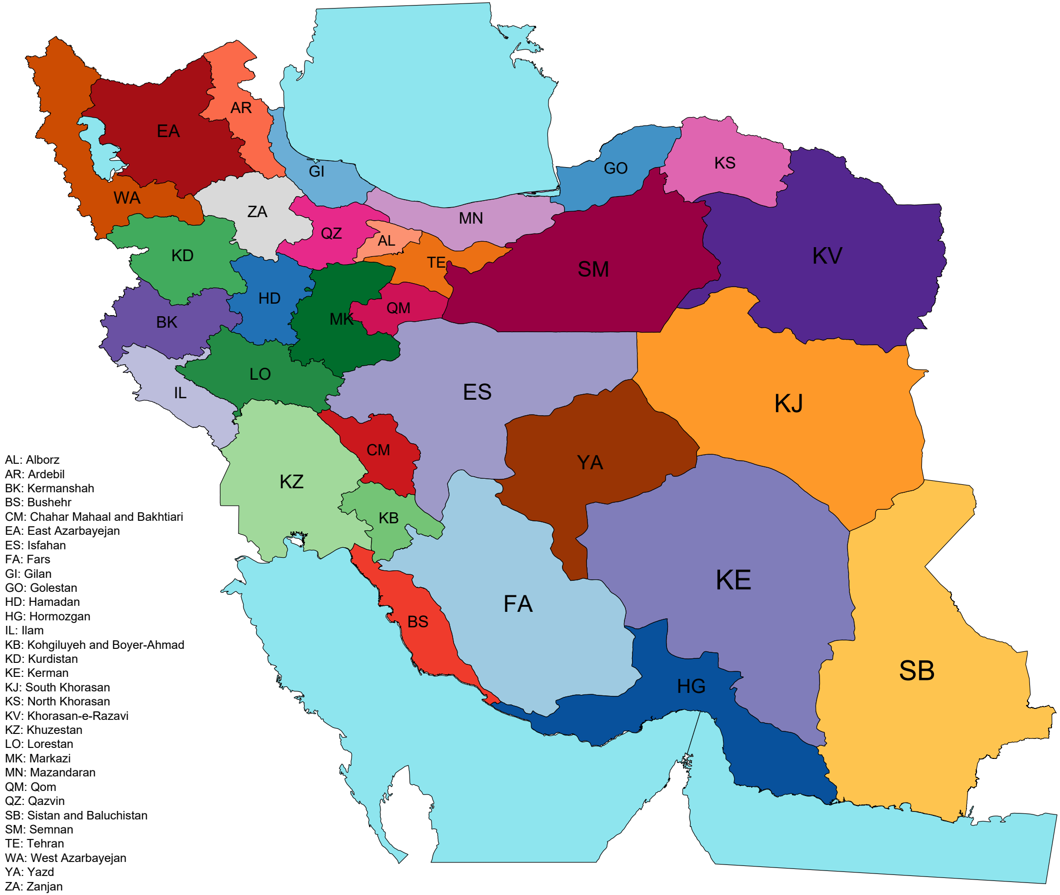
AR
EA
WA
GI
ZA
QZ
AL
MN
TE
GO
KS
KV
SM
KD
HD
MK
QM
BK
LO
IL
ES
KJ
YA
CM
KZ
KB
FA
KE
BS
HG
SB
AL: Alborz
AR: Ardebil
BK: Kermanshah
BS: Bushehr
CM: Chahar Mahaal and Bakhtiari
EA: East Azarbayejan
ES: Isfahan
FA: Fars
GI: Gilan
GO: Golestan
HD: Hamadan
HG: Hormozgan
IL: Ilam
KB: Kohgiluyeh and Boyer-Ahmad
KD: Kurdistan
KE: Kerman
KJ: South Khorasan
KS: North Khorasan
KV: Khorasan-e-Razavi
KZ: Khuzestan
LO: Lorestan
MK: Markazi
MN: Mazandaran
QM: Qom
QZ: Qazvin
SB: Sistan and Baluchistan
SM: Semnan
TE: Tehran
WA: West Azarbayejan
YA: Yazd
ZA: Zanjan
