# Supplementary material for: National, sub-national, and risk-attributed burden of thyroid cancer in Iran from 1990 to 2019
Source: Sci Rep. 2022 Aug 2;12:13231. doi: 10.1038/s41598-022-17115-0 (PMC9346133; doi:10.1038/s41598-022-17115-0)
Supplement: Supplementary file 5 — Supplementary Table 1. [file 41598_2022_17115_MOESM5_ESM.pdf]

Province	Measure	Age-standardized rate (per 100,000)						% Change (1990 to 2019)		
		1990			2019					
		Both	Female	Male	Both	Female	Male	Both	Female	Male
Alborz	Incidence	2.45 (1.37 to 3.34)	4.02 (2.04 to 5.95)	1.05 (0.64 to 1.54)	4.86 (2.66 to 6.27)	6.66 (3.38 to 9.15)	3.13 (1.87 to 4.33)	98.7 (34.3 to 251.1)	65.7 (2.5 to 230.4)	199.1 (73.6 to 449.2)
	Prevalence	18.48 (9.83 to 26.19)	31.53 (15.2 to 48.39)	7.11 (3.85 to 10.76)	41.2 (21.75 to 53.68)	57.21 (28.33 to 79.68)	25.64 (14.73 to 35.97)	123 (47.1 to 300)	81.5 (7.5 to 301.2)	260.6 (104.6 to 624.2)
	Deaths	0.57 (0.39 to 0.74)	0.79 (0.46 to 1.07)	0.35 (0.23 to 0.5)	0.55 (0.38 to 0.66)	0.65 (0.4 to 0.82)	0.48 (0.33 to 0.62)	-2.3 (-30.4 to 42.6)	-18.5 (-43.8 to 31.4)	39.2 (-13.6 to 130.2)
	DALYs	14.37 (9 to 18.38)	20.06 (10.37 to 27.31)	9.13 (5.74 to 13.02)	15.15 (9.19 to 18.31)	17.06 (9.53 to 22.06)	13.43 (8.38 to 17.1)	5.4 (-22.1 to 63.7)	-14.9 (-41.4 to 52.4)	47.2 (-7.8 to 140.2)
	YLLs	13.2 (8.22 to 16.95)	18.12 (9.35 to 24.85)	8.63 (5.42 to 12.52)	12.73 (7.93 to 15.31)	13.77 (7.88 to 17.87)	11.84 (7.44 to 15.25)	-3.6 (-30 to 45.8)	-24 (-48.3 to 30.3)	37.2 (-15.9 to 130.5)
	YLDs	1.17 (0.54 to 1.83)	1.94 (0.83 to 3.27)	0.49 (0.24 to 0.86)	2.42 (1.22 to 3.66)	3.29 (1.45 to 5.24)	1.59 (0.82 to 2.54)	107.2 (35.6 to 285.4)	70 (0.7 to 241.5)	221.7 (79.6 to 530.9)
Ardebil	Incidence	1.33 (0.97 to 1.77)	2.12 (1.43 to 2.99)	0.59 (0.4 to 0.86)	3.55 (2.34 to 4.43)	5.05 (3.02 to 6.76)	2.05 (1.5 to 2.67)	167.2 (70.1 to 302.3)	137.9 (38.8 to 289)	246.7 (104.4 to 469.8)
	Prevalence	9.42 (6.41 to 13.4)	15.72 (9.87 to 23.19)	3.59 (2.3 to 5.46)	29.22 (18.63 to 37.02)	42.86 (25.11 to 57.85)	15.74 (10.95 to 21.1)	210.3 (91.9 to 388.9)	172.6 (56.4 to 382.9)	338.1 (144.8 to 646.5)
	Deaths	0.39 (0.31 to 0.5)	0.55 (0.41 to 0.74)	0.25 (0.17 to 0.36)	0.51 (0.4 to 0.6)	0.56 (0.38 to 0.7)	0.46 (0.36 to 0.57)	29.4 (-10.5 to 77.8)	1.1 (-37.6 to 48.4)	81.9 (15.7 to 190.7)
	DALYs	10.1 (7.72 to 12.5)	14.08 (10.19 to 18.47)	6.58 (4.51 to 9.43)	13.62 (9.64 to 16.07)	15.41 (9.59 to 19.4)	11.81 (9.07 to 14.51)	34.9 (-10.3 to 90.4)	9.4 (-33.4 to 64.3)	79.5 (13.2 to 186.3)
	YLLs	9.49 (7.27 to 11.77)	13.1 (9.5 to 17.53)	6.31 (4.32 to 9.09)	11.86 (8.58 to 13.92)	12.89 (8.14 to 16.08)	10.79 (8.26 to 13.29)	25 (-16.3 to 75.2)	-1.6 (-40.6 to 48.2)	70.9 (7.7 to 173.3)
	YLDs	0.61 (0.36 to 0.93)	0.98 (0.53 to 1.57)	0.26 (0.15 to 0.44)	1.77 (0.99 to 2.67)	2.52 (1.28 to 4.01)	1.02 (0.59 to 1.58)	190.3 (80.7 to 358.8)	156 (46.4 to 356.9)	287 (111.5 to 559.7)

Province	Measure	Age-standardized rate (per 100,000)						% Change (1990 to 2019)		
		1990			2019					
		Both	Female	Male	Both	Female	Male	Both	Female	Male
Bushehr	Incidence	1.3 (0.92 to 1.8)	2.04 (1.36 to 2.92)	0.59 (0.4 to 0.88)	4.44 (2.46 to 5.58)	7.29 (3.31 to 9.62)	1.81 (1.36 to 2.32)	242.2 (53.2 to 451.1)	257.3 (37 to 538.3)	209.6 (77.5 to 403.6)
	Prevalence	9.17 (6.16 to 12.66)	15 (9.41 to 21.68)	3.68 (2.34 to 5.55)	37.16 (20.07 to 46.95)	62.42 (27.53 to 83.03)	14.01 (10.22 to 18.39)	305.4 (75.9 to 597.7)	316.1 (53.8 to 689.5)	281 (116.5 to 554.3)
	Deaths	0.38 (0.28 to 0.55)	0.53 (0.36 to 0.81)	0.23 (0.15 to 0.35)	0.57 (0.39 to 0.67)	0.74 (0.38 to 0.92)	0.39 (0.3 to 0.49)	48.5 (-18.5 to 118.1)	40.1 (-39 to 122.2)	69.3 (3.4 to 166)
	DALYs	9.16 (6.82 to 12.4)	12.6 (8.94 to 18.4)	5.83 (3.93 to 8.82)	15.05 (9.55 to 18.08)	20.55 (9.69 to 26.11)	9.76 (7.66 to 12.19)	64.2 (-15.3 to 139.9)	63.1 (-32.6 to 149.1)	67.3 (1.5 to 162.9)
	YLLs	8.56 (6.37 to 11.71)	11.64 (8.22 to 17.14)	5.56 (3.72 to 8.37)	12.84 (8.4 to 15.21)	16.94 (8.08 to 21.06)	8.85 (6.87 to 11.06)	50 (-20.9 to 122.9)	45.5 (-38.4 to 127.6)	59.2 (-3.7 to 153)
	YLDs	0.61 (0.34 to 0.94)	0.96 (0.51 to 1.54)	0.27 (0.15 to 0.44)	2.2 (1.11 to 3.34)	3.61 (1.45 to 5.7)	0.91 (0.55 to 1.37)	264.3 (61.8 to 502.1)	276.5 (39.7 to 577.7)	236.9 (88.9 to 471.6)
Chahar Mahaal and Bakhtiari	Incidence	1.32 (0.99 to 1.8)	1.94 (1.35 to 2.91)	0.75 (0.51 to 1.05)	3.01 (2.03 to 3.92)	3.9 (2.5 to 5.53)	2.14 (1.37 to 2.96)	128.2 (38.5 to 231)	101 (11.6 to 231)	184.5 (61.8 to 385.8)
	Prevalence	9.56 (7.02 to 13.21)	14.73 (9.93 to 22.02)	4.96 (3.18 to 7.19)	25.38 (16.8 to 33.22)	33.76 (21.49 to 47.95)	17.16 (10.57 to 24.06)	165.4 (56.8 to 305.6)	129.2 (23.6 to 306.5)	245.8 (88.2 to 511.4)
	Deaths	0.36 (0.28 to 0.51)	0.45 (0.32 to 0.75)	0.27 (0.19 to 0.37)	0.36 (0.28 to 0.44)	0.34 (0.24 to 0.46)	0.38 (0.27 to 0.5)	-0.2 (-31.9 to 42.1)	-25.1 (-53.7 to 15.6)	42.3 (-16.8 to 128.4)
	DALYs	8.81 (6.94 to 11.82)	10.88 (7.92 to 16.39)	6.89 (4.74 to 9.68)	9.87 (7.1 to 12.23)	9.67 (6.58 to 13.12)	10.1 (6.9 to 13.1)	12.1 (-24.8 to 58)	-11.1 (-44.3 to 34.9)	46.5 (-14.9 to 137.4)
	YLLs	8.19 (6.42 to 10.9)	9.96 (7.07 to 15.13)	6.54 (4.47 to 9.17)	8.37 (6.07 to 10.35)	7.73 (5.27 to 10.47)	9.01 (6.18 to 11.68)	2.2 (-31.9 to 45.9)	-22.4 (-52.2 to 18.7)	37.7 (-20.6 to 122.9)
	YLDs	0.62 (0.37 to 0.97)	0.92 (0.53 to 1.53)	0.35 (0.19 to 0.58)	1.51 (0.86 to 2.28)	1.94 (1.03 to 3.13)	1.09 (0.6 to 1.81)	144.1 (46.9 to 279.3)	111.3 (11.2 to 266.9)	212 (64.1 to 477.7)

Province	Measure	Age-standardized rate (per 100,000)						% Change (1990 to 2019)		
		1990			2019					
		Both	Female	Male	Both	Female	Male	Both	Female	Male
East Azarbayejan	Incidence	1.17 (0.84 to 1.72)	1.72 (1.16 to 2.69)	0.66 (0.43 to 0.93)	4.41 (2.52 to 5.66)	6.09 (3.23 to 8.26)	2.76 (1.52 to 3.71)	277.7 (60 to 508.4)	254.6 (38.7 to 525)	320.4 (88.7 to 601.3)
	Prevalence	7.92 (5.53 to 11.77)	12.3 (8.07 to 19.54)	3.89 (2.47 to 5.64)	36.34 (20.04 to 46.86)	51.38 (26.57 to 70.86)	21.5 (11.49 to 29.37)	358.9 (84.9 to 673.5)	317.8 (56.2 to 705.6)	453 (146.4 to 869.6)
	Deaths	0.39 (0.29 to 0.58)	0.5 (0.34 to 0.84)	0.29 (0.2 to 0.42)	0.64 (0.44 to 0.77)	0.72 (0.45 to 0.91)	0.57 (0.38 to 0.73)	64.5 (-15.2 to 142.5)	44.1 (-38.3 to 134)	97.7 (2.6 to 218.8)
	DALYs	9.32 (7.05 to 13.33)	11.58 (8.18 to 18.69)	7.22 (4.98 to 10.23)	16.81 (10.29 to 20.29)	18.69 (10.74 to 23.91)	14.9 (8.96 to 19.38)	80.4 (-15.2 to 162.6)	61.4 (-34.5 to 158.2)	106.4 (0.3 to 232.3)
	YLLs	8.79 (6.6 to 12.69)	10.79 (7.52 to 17.56)	6.93 (4.74 to 9.94)	14.63 (8.94 to 17.76)	15.69 (9.16 to 20.23)	13.54 (8.22 to 17.72)	66.5 (-20.9 to 144.1)	45.4 (-40.3 to 138.3)	95.4 (-4.9 to 217.4)
	YLDs	0.53 (0.32 to 0.84)	0.79 (0.44 to 1.36)	0.29 (0.16 to 0.47)	2.18 (1.12 to 3.29)	3 (1.37 to 4.8)	1.37 (0.67 to 2.11)	311.1 (68.3 to 577.8)	280.3 (43.5 to 616.3)	370 (105 to 757)
Fars	Incidence	1.16 (0.81 to 1.87)	1.7 (1.09 to 2.8)	0.64 (0.41 to 1.08)	3.45 (2.65 to 4.35)	4.82 (3.35 to 6.52)	2.1 (1.47 to 2.79)	197.4 (59.7 to 380)	183.8 (39.7 to 406.5)	226.9 (74.2 to 471.3)
	Prevalence	8.4 (5.67 to 13.66)	12.96 (8.03 to 21.87)	4.16 (2.54 to 7.24)	29.15 (22.01 to 37.03)	41.47 (28.37 to 56.72)	17.02 (11.61 to 23.07)	247 (82.4 to 490)	220.1 (53.5 to 504.5)	309.2 (108.2 to 654.9)
	Deaths	0.32 (0.23 to 0.53)	0.39 (0.26 to 0.74)	0.24 (0.16 to 0.38)	0.4 (0.33 to 0.48)	0.46 (0.35 to 0.6)	0.34 (0.26 to 0.44)	26.2 (-19.4 to 87)	17.9 (-36.6 to 88.7)	44.9 (-12.3 to 141.6)
	DALYs	7.83 (5.83 to 12.36)	9.57 (6.61 to 16.79)	6.08 (4.14 to 9.72)	11.33 (9.36 to 13.67)	12.95 (9.42 to 16.76)	9.73 (7.46 to 12.43)	44.7 (-12.5 to 108.7)	35.3 (-29.8 to 112)	60.1 (-4.4 to 159.7)
	YLLs	7.29 (5.42 to 11.56)	8.77 (6.02 to 15.76)	5.78 (3.93 to 9.25)	9.61 (7.94 to 11.46)	10.55 (7.82 to 13.7)	8.67 (6.56 to 11.07)	31.8 (-19 to 95.1)	20.3 (-36.3 to 93.7)	50 (-11.3 to 151.8)
	YLDs	0.54 (0.3 to 0.91)	0.8 (0.42 to 1.4)	0.3 (0.15 to 0.53)	1.72 (1.07 to 2.61)	2.4 (1.32 to 3.91)	1.06 (0.64 to 1.67)	218.2 (66.1 to 443.3)	199.5 (44.2 to 472.7)	257.4 (69.5 to 579.5)

Province	Measure	Age-standardized rate (per 100,000)						% Change (1990 to 2019)		
		1990			2019					
		Both	Female	Male	Both	Female	Male	Both	Female	Male
Gilan	Incidence	1.34 (0.96 to 2)	1.97 (1.31 to 3.04)	0.68 (0.46 to 1.01)	3.59 (2.6 to 4.52)	5.06 (3.37 to 6.81)	2.09 (1.46 to 2.76)	168.5 (43.8 to 322.7)	156.7 (27.6 to 358.4)	205.8 (73.9 to 417.8)
	Prevalence	9.11 (6.25 to 13.54)	14.27 (9.18 to 21.97)	4.02 (2.57 to 6.07)	30.08 (21.26 to 37.81)	43.16 (27.85 to 58.77)	16.78 (11.44 to 22.52)	230.2 (70.4 to 452.3)	202.5 (45.4 to 466.8)	317.7 (129.2 to 643.6)
	Deaths	0.35 (0.26 to 0.55)	0.43 (0.31 to 0.74)	0.25 (0.17 to 0.38)	0.45 (0.37 to 0.53)	0.53 (0.39 to 0.67)	0.36 (0.27 to 0.46)	26.8 (-20.6 to 78)	22.4 (-31.5 to 88.8)	45.2 (-11.7 to 124.5)
	DALYs	8.73 (6.61 to 12.7)	10.82 (7.75 to 17.14)	6.42 (4.48 to 9.47)	12.22 (9.64 to 14.39)	14.33 (10.08 to 18.27)	10.04 (7.66 to 12.43)	39.9 (-15.7 to 95.5)	32.5 (-29.2 to 106.4)	56.4 (-7.1 to 140.9)
	YLLs	8.04 (6.04 to 11.77)	9.8 (6.91 to 15.67)	6.07 (4.2 to 8.97)	10.43 (8.31 to 12.3)	11.82 (8.4 to 15.05)	8.98 (6.78 to 11.23)	29.6 (-22.6 to 83)	20.7 (-35.5 to 93.5)	48 (-12.2 to 131.2)
	YLDs	0.69 (0.41 to 1.08)	1.02 (0.57 to 1.7)	0.35 (0.2 to 0.56)	1.79 (1.05 to 2.69)	2.51 (1.4 to 4.03)	1.06 (0.62 to 1.62)	160.9 (37 to 329)	146.5 (18 to 346.4)	200.8 (61.1 to 437.5)
Golestan	Incidence	0.94 (0.67 to 1.59)	1.33 (0.86 to 2.48)	0.56 (0.38 to 0.82)	3.41 (2.52 to 4.16)	4.44 (3.13 to 5.79)	2.34 (1.59 to 3.06)	263.4 (80.4 to 456.6)	234.6 (54.1 to 460.2)	317.4 (100 to 586.4)
	Prevalence	6.17 (4.2 to 10.72)	9.37 (5.82 to 17.42)	3.13 (1.97 to 4.76)	27.75 (20.07 to 34.32)	37.41 (26.02 to 49.5)	17.79 (11.53 to 23.54)	349.9 (114.6 to 612.9)	299.1 (80.9 to 611.6)	467.6 (165.7 to 918.9)
	Deaths	0.33 (0.25 to 0.57)	0.39 (0.26 to 0.78)	0.27 (0.19 to 0.41)	0.53 (0.44 to 0.62)	0.52 (0.41 to 0.64)	0.54 (0.39 to 0.68)	60.2 (-10.5 to 129.3)	34.2 (-35.7 to 116.6)	100.5 (10.9 to 212.6)
	DALYs	8.22 (6.16 to 13.87)	9.71 (6.65 to 18.21)	6.77 (4.71 to 10.31)	14.7 (11.57 to 17.36)	14.9 (11.11 to 18.68)	14.5 (10.46 to 18.44)	78.8 (-5.9 to 156.7)	53.4 (-26.8 to 141.4)	114.1 (13.4 to 240.1)
	YLLs	7.8 (5.84 to 13.3)	9.1 (6.15 to 17.01)	6.52 (4.5 to 9.94)	13 (10.27 to 15.37)	12.69 (9.57 to 15.84)	13.33 (9.58 to 16.97)	66.7 (-10.5 to 141.6)	39.4 (-33.2 to 120.6)	104.3 (8.6 to 227.3)
	YLDs	0.42 (0.24 to 0.73)	0.61 (0.31 to 1.14)	0.25 (0.14 to 0.39)	1.7 (1.04 to 2.48)	2.21 (1.29 to 3.4)	1.17 (0.64 to 1.79)	301.2 (91.9 to 560.5)	262.2 (61.3 to 595.1)	376.5 (121.1 to 724.2)

Province	Measure	Age-standardized rate (per 100,000)						% Change (1990 to 2019)		
		1990			2019					
		Both	Female	Male	Both	Female	Male	Both	Female	Male
Hamadan	Incidence	1.35 (1.02 to 1.79)	1.93 (1.36 to 2.75)	0.8 (0.56 to 1.14)	4.15 (2.38 to 5.2)	5.18 (2.96 to 7.07)	3.13 (1.62 to 4.35)	208 (54.1 to 347.6)	168.5 (31 to 331.1)	290.7 (90.7 to 550)
	Prevalence	9.66 (7.07 to 13.1)	14.39 (9.79 to 20.75)	5.23 (3.51 to 7.68)	34.58 (19.29 to 43.81)	44.03 (24.6 to 60.42)	25.05 (12.53 to 35.34)	258.1 (75.4 to 439.4)	206 (49.3 to 419)	378.5 (131.2 to 727.9)
	Deaths	0.39 (0.3 to 0.53)	0.49 (0.35 to 0.75)	0.3 (0.21 to 0.41)	0.57 (0.38 to 0.69)	0.58 (0.4 to 0.73)	0.56 (0.35 to 0.72)	46.2 (-16.7 to 111.2)	19.1 (-35.9 to 87.2)	91.1 (0.4 to 202)
	DALYs	10 (7.86 to 12.94)	12.02 (8.83 to 16.94)	8.11 (5.81 to 11.25)	16.16 (9.7 to 19.73)	15.91 (9.81 to 20.17)	16.4 (9.23 to 21.03)	61.6 (-14.5 to 132)	32.3 (-30.2 to 103.6)	102.1 (4.3 to 218.8)
	YLLs	9.38 (7.29 to 12.15)	11.12 (7.97 to 15.85)	7.74 (5.51 to 10.84)	14.1 (8.55 to 17.33)	13.36 (8.6 to 17.14)	14.82 (8.4 to 19.26)	50.3 (-19.7 to 118.3)	20.1 (-35.8 to 88.3)	91.3 (-1.6 to 205.9)
	YLDs	0.62 (0.39 to 0.96)	0.9 (0.52 to 1.44)	0.37 (0.21 to 0.6)	2.07 (1.1 to 3.11)	2.55 (1.24 to 4.03)	1.58 (0.76 to 2.56)	231.1 (61.7 to 400.6)	183.8 (35.3 to 396.6)	329.9 (102.6 to 696.6)
Hormozgan	Incidence	0.78 (0.48 to 1.3)	1.05 (0.6 to 2.1)	0.53 (0.32 to 0.77)	2.31 (1.83 to 2.91)	2.65 (1.97 to 3.72)	1.98 (1.33 to 2.64)	197 (67.6 to 415.3)	151.7 (42.2 to 380.8)	273.7 (103.9 to 583.1)
	Prevalence	4.73 (2.49 to 8.29)	6.98 (3.42 to 14)	2.78 (1.5 to 4.21)	18.9 (14.81 to 24.01)	22.23 (16.34 to 30.72)	15.59 (10 to 21.35)	299.5 (114.9 to 672.2)	218.3 (70 to 575.5)	461.6 (203.1 to 1021.9)
	Deaths	0.32 (0.22 to 0.54)	0.37 (0.24 to 0.75)	0.27 (0.18 to 0.4)	0.36 (0.3 to 0.45)	0.33 (0.26 to 0.51)	0.39 (0.28 to 0.5)	12.5 (-26.8 to 67)	-9.1 (-39.9 to 41)	44.1 (-17.3 to 139.6)
	DALYs	7.94 (5.26 to 12.64)	8.88 (5.47 to 17.83)	7.03 (4.45 to 9.99)	10.04 (8.17 to 12.26)	8.86 (6.95 to 12.45)	11.24 (7.92 to 14.16)	26.5 (-22.9 to 96.8)	-0.3 (-37.6 to 65.5)	59.9 (-9.7 to 172.4)
	YLLs	7.6 (5.07 to 12.18)	8.41 (5.21 to 16.62)	6.81 (4.3 to 9.7)	8.89 (7.27 to 10.81)	7.56 (5.93 to 10.73)	10.25 (7.11 to 12.97)	17.1 (-27.8 to 80.4)	-10.1 (-44.1 to 50.5)	50.6 (-15.6 to 155.7)
	YLDs	0.34 (0.18 to 0.6)	0.47 (0.23 to 0.94)	0.22 (0.12 to 0.37)	1.15 (0.73 to 1.73)	1.3 (0.77 to 2.08)	0.99 (0.55 to 1.62)	237.1 (78.1 to 499.1)	176.7 (46.4 to 483.4)	341.6 (135.9 to 743.9)

Province	Measure	Age-standardized rate (per 100,000)						% Change (1990 to 2019)		
		1990			2019					
		Both	Female	Male	Both	Female	Male	Both	Female	Male
Ilam	Incidence	0.92 (0.67 to 1.53)	1.41 (0.93 to 2.57)	0.53 (0.34 to 0.83)	3.17 (2.5 to 3.88)	4.14 (3.07 to 5.39)	2.19 (1.57 to 2.82)	243 (89.6 to 425)	193.3 (53 to 394.3)	314.5 (116.4 to 603.2)
	Prevalence	6.39 (4.36 to 10.66)	10.24 (6.47 to 18.85)	3.27 (2.01 to 5.1)	26.47 (20.55 to 32.74)	35.4 (25.86 to 46.52)	17.45 (12.1 to 23.03)	314.2 (119.7 to 573.9)	245.6 (75.3 to 508.2)	433.5 (179.4 to 859)
	Deaths	0.29 (0.2 to 0.47)	0.38 (0.25 to 0.77)	0.21 (0.14 to 0.34)	0.42 (0.36 to 0.49)	0.43 (0.35 to 0.54)	0.41 (0.32 to 0.5)	46.7 (-6.6 to 116.9)	12.9 (-37.8 to 84)	92.4 (12.3 to 209.2)
	DALYs	7.04 (5.06 to 11.08)	9.11 (6.17 to 16.81)	5.45 (3.66 to 8.31)	11.59 (9.4 to 13.43)	12.04 (9.49 to 14.84)	11.06 (8.43 to 13.57)	64.6 (-2.2 to 141)	32.1 (-27.5 to 114.1)	103.1 (14.1 to 223.1)
	YLLs	6.62 (4.69 to 10.38)	8.46 (5.67 to 15.73)	5.21 (3.46 to 7.97)	10.02 (8.25 to 11.55)	9.99 (7.85 to 12.3)	9.97 (7.62 to 12.34)	51.4 (-9.1 to 121)	18.1 (-35.7 to 93.7)	91.4 (7.6 to 205.3)
	YLDs	0.43 (0.24 to 0.74)	0.66 (0.34 to 1.26)	0.24 (0.13 to 0.4)	1.57 (0.98 to 2.32)	2.05 (1.2 to 3.1)	1.1 (0.64 to 1.66)	269.2 (93.2 to 488.8)	212.3 (51.2 to 473.7)	354.1 (129.4 to 718.7)
Isfahan	Incidence	1.4 (0.93 to 1.98)	2.12 (1.27 to 3.15)	0.71 (0.48 to 1.04)	3.93 (2.32 to 5.03)	5.78 (3.03 to 7.83)	2.13 (1.49 to 2.9)	180.6 (43.6 to 366.1)	172.9 (23.5 to 411.6)	199.7 (64.2 to 413.6)
	Prevalence	10.42 (6.76 to 15)	16.45 (9.6 to 25.05)	4.83 (3.15 to 7.21)	33.06 (18.86 to 42.8)	49.32 (25.25 to 67.74)	17.17 (11.66 to 23.73)	217.2 (60 to 443.5)	199.8 (34.1 to 475.6)	255.6 (92.5 to 530.6)
	Deaths	0.35 (0.25 to 0.51)	0.45 (0.3 to 0.73)	0.24 (0.16 to 0.34)	0.49 (0.34 to 0.58)	0.61 (0.38 to 0.78)	0.37 (0.28 to 0.48)	37.9 (-20.8 to 108.9)	35.2 (-33.9 to 129.2)	55.3 (-4.8 to 146.9)
	DALYs	8.47 (6.13 to 11.56)	10.78 (6.99 to 16.25)	6.07 (4.07 to 8.59)	13.03 (8.43 to 15.75)	16.06 (9.14 to 20.32)	10.08 (7.51 to 12.8)	53.8 (-13.1 to 127.7)	49 (-27.1 to 145.4)	66.1 (-0.9 to 165.7)
	YLLs	7.81 (5.63 to 10.7)	9.78 (6.31 to 15.06)	5.74 (3.77 to 8.15)	11.08 (7.25 to 13.41)	13.21 (7.5 to 17.06)	9.01 (6.55 to 11.5)	41.9 (-18.9 to 114.7)	35.2 (-34.1 to 129.4)	57.1 (-5.8 to 154.2)
	YLDs	0.66 (0.36 to 1.05)	1 (0.51 to 1.71)	0.33 (0.18 to 0.55)	1.95 (1.06 to 2.96)	2.85 (1.36 to 4.58)	1.07 (0.6 to 1.68)	195.7 (50.7 to 389.6)	184.3 (25.8 to 440.3)	220.4 (61.8 to 479.4)

Province	Measure	Age-standardized rate (per 100,000)						% Change (1990 to 2019)		
		1990			2019					
		Both	Female	Male	Both	Female	Male	Both	Female	Male
**Kerman**	**Incidence**	1.15 (0.87 to 1.63)	1.67 (1.19 to 2.5)	0.66 (0.46 to 0.97)	3.75 (2.14 to 4.77)	5.44 (2.75 to 7.38)	2.12 (1.37 to 2.87)	224.7 (43.8 to 377.6)	225.1 (28.9 to 427.1)	221.2 (57.1 to 428.4)
	**Prevalence**	7.7 (5.6 to 11)	11.9 (8.1 to 18)	3.81 (2.49 to 5.77)	30.71 (16.92 to 39.61)	45.57 (22.54 to 62.6)	16.42 (9.97 to 22.82)	299 (70.8 to 526.4)	283 (47.1 to 564.9)	331.4 (101.9 to 641.3)
	**Deaths**	0.39 (0.3 to 0.57)	0.48 (0.35 to 0.78)	0.3 (0.21 to 0.44)	0.56 (0.38 to 0.67)	0.67 (0.41 to 0.84)	0.45 (0.33 to 0.57)	43.3 (-25.8 to 99.7)	39 (-35.6 to 107.4)	53.8 (-17.3 to 134.1)
	**DALYs**	9.63 (7.49 to 13.64)	11.75 (8.58 to 17.51)	7.57 (5.35 to 10.86)	15.14 (9.1 to 18.34)	17.97 (9.84 to 22.86)	12.36 (8.29 to 15.67)	57.2 (-25.7 to 118.7)	53 (-34.1 to 133)	63.3 (-18.3 to 149.4)
	**YLLs**	9.1 (7 to 12.94)	10.97 (7.91 to 16.37)	7.28 (5.12 to 10.51)	13.29 (8.21 to 16.12)	15.29 (8.55 to 19.93)	11.31 (7.49 to 14.26)	45.9 (-30 to 106.6)	39.4 (-39.2 to 114.2)	55.4 (-21.5 to 139.5)
	**YLDs**	0.53 (0.31 to 0.86)	0.78 (0.43 to 1.31)	0.3 (0.17 to 0.48)	1.85 (0.95 to 2.76)	2.68 (1.17 to 4.14)	1.06 (0.58 to 1.65)	250.2 (53.3 to 453.1)	244.2 (28.9 to 514.7)	257.9 (69.1 to 521.1)
**Kermanshah**	**Incidence**	1.11 (0.81 to 1.64)	1.6 (1.09 to 2.6)	0.68 (0.46 to 1.01)	3.94 (2.38 to 5)	5.3 (3.02 to 7.23)	2.57 (1.51 to 3.51)	253.9 (56 to 444.8)	230.5 (35.9 to 467)	276 (76.3 to 516.9)
	**Prevalence**	7.36 (5.05 to 11.15)	11.29 (7.31 to 18.58)	3.91 (2.53 to 6.05)	32.64 (19.31 to 41.73)	44.72 (25.1 to 61.74)	20.29 (11.4 to 28.18)	343.3 (85.4 to 617.9)	296.2 (55.5 to 619.9)	418.3 (133.6 to 796.9)
	**Deaths**	0.39 (0.29 to 0.57)	0.48 (0.34 to 0.78)	0.31 (0.21 to 0.44)	0.56 (0.39 to 0.67)	0.62 (0.41 to 0.8)	0.5 (0.33 to 0.65)	44 (-24.2 to 113.3)	30.4 (-39.2 to 107.6)	60.7 (-16.5 to 162.8)
	**DALYs**	9.6 (7.19 to 13.88)	11.55 (8.28 to 18.48)	7.94 (5.49 to 11.42)	15.59 (9.9 to 18.89)	17.03 (10.44 to 21.91)	14.11 (8.81 to 18.28)	62.4 (-23.5 to 141.1)	47.4 (-35.7 to 131.8)	77.6 (-15.2 to 191.9)
	**YLLs**	9.1 (6.82 to 13.2)	10.81 (7.71 to 17.52)	7.64 (5.27 to 11.02)	13.63 (8.78 to 16.58)	14.41 (8.82 to 18.62)	12.83 (7.98 to 16.84)	49.8 (-28.3 to 124.4)	33.3 (-42 to 112.5)	67.9 (-19.1 to 181.3)
	**YLDs**	0.5 (0.3 to 0.83)	0.74 (0.41 to 1.29)	0.3 (0.17 to 0.48)	1.96 (1.03 to 2.98)	2.62 (1.28 to 4.22)	1.28 (0.66 to 2.06)	288.5 (65.3 to 536.3)	255.9 (40.1 to 556.2)	326.1 (95.2 to 674.3)

Province	Measure	Age-standardized rate (per 100,000)						% Change (1990 to 2019)		
		1990			2019					
		Both	Female	Male	Both	Female	Male	Both	Female	Male
Khorasan-e-Razavi	Incidence	1.01 (0.7 to 1.76)	1.46 (0.95 to 2.66)	0.58 (0.39 to 0.91)	3.05 (2.36 to 3.77)	4.35 (3.12 to 5.62)	1.75 (1.27 to 2.36)	202.7 (51 to 368.2)	197.2 (33.9 to 395.8)	201.3 (58.5 to 398.1)
	Prevalence	6.68 (4.49 to 11.72)	10.31 (6.3 to 18.93)	3.31 (2.11 to 5.25)	25.11 (18.87 to 31.3)	36.62 (25.75 to 47.8)	13.51 (9.58 to 18.69)	275.8 (79 to 514.4)	255.3 (56.9 to 539.8)	308.5 (106 to 624.1)
	Deaths	0.35 (0.26 to 0.59)	0.43 (0.3 to 0.81)	0.27 (0.19 to 0.43)	0.45 (0.37 to 0.53)	0.52 (0.41 to 0.65)	0.38 (0.29 to 0.47)	28.2 (-25.8 to 84.3)	19.6 (-38.2 to 86.4)	40.7 (-17 to 122.3)
	DALYs	8.65 (6.41 to 14.53)	10.59 (7.3 to 19.28)	6.83 (4.72 to 10.67)	12 (9.6 to 14.31)	13.96 (10.59 to 17.46)	9.99 (7.79 to 12.58)	38.8 (-24.7 to 101)	31.8 (-35.2 to 105.3)	46.3 (-19.6 to 129)
	YLLs	8.19 (6.06 to 13.84)	9.92 (6.8 to 18.15)	6.57 (4.55 to 10.28)	10.5 (8.5 to 12.43)	11.81 (8.98 to 14.93)	9.13 (7 to 11.54)	28.2 (-30.1 to 87.2)	19.1 (-40.8 to 87.8)	38.8 (-22.2 to 117.4)
	YLDs	0.46 (0.27 to 0.81)	0.67 (0.36 to 1.3)	0.25 (0.14 to 0.43)	1.51 (0.9 to 2.2)	2.14 (1.23 to 3.29)	0.86 (0.5 to 1.38)	229.8 (56.7 to 440)	217.7 (35.6 to 473.1)	240.8 (70.8 to 520.1)
Khuzestan	Incidence	1.36 (1.02 to 1.8)	2.19 (1.52 to 3.02)	0.56 (0.37 to 0.81)	3.95 (2.41 to 5.08)	6.23 (3.29 to 8.39)	1.7 (1.26 to 2.26)	190.7 (57.1 to 345.2)	184.1 (43.4 to 359.3)	204.4 (79.8 to 391.1)
	Prevalence	9.62 (6.74 to 13.25)	16.28 (10.78 to 23.08)	3.43 (2.25 to 5.12)	32.93 (19.53 to 42.72)	52.79 (27.22 to 71.7)	13.31 (9.63 to 18.03)	242.2 (82.7 to 438.3)	224.2 (60.1 to 449.7)	287.5 (121.7 to 542)
	Deaths	0.4 (0.31 to 0.54)	0.55 (0.41 to 0.78)	0.23 (0.16 to 0.36)	0.52 (0.39 to 0.63)	0.7 (0.43 to 0.88)	0.34 (0.26 to 0.43)	30.5 (-19.3 to 83)	26.2 (-33.4 to 87.5)	48.5 (-8.5 to 127.5)
	DALYs	9.88 (7.85 to 12.76)	13.85 (10.29 to 18.52)	5.93 (4.13 to 8.98)	14.36 (9.82 to 17.24)	19.37 (10.66 to 24.49)	9.32 (7.21 to 11.85)	45.4 (-13.6 to 101.9)	39.8 (-29.9 to 109.2)	57.1 (-4.5 to 139.1)
	YLLs	9.25 (7.28 to 12.07)	12.83 (9.36 to 17.36)	5.68 (3.92 to 8.7)	12.4 (8.73 to 15)	16.29 (9.14 to 20.77)	8.46 (6.53 to 10.75)	34 (-20.3 to 88)	26.9 (-35.6 to 93.2)	49.1 (-9.3 to 128.4)
	YLDs	0.63 (0.38 to 0.97)	1.02 (0.58 to 1.62)	0.25 (0.15 to 0.41)	1.96 (0.99 to 2.97)	3.08 (1.37 to 4.85)	0.85 (0.49 to 1.36)	213.1 (69.2 to 384.5)	201.5 (49.1 to 415.6)	237 (89.1 to 494.1)

Province	Measure	Age-standardized rate (per 100,000)						% Change (1990 to 2019)		
		1990			2019					
		Both	Female	Male	Both	Female	Male	Both	Female	Male
Kohgiluyeh and Boyer-Ahmad	Incidence	1.32 (0.96 to 1.78)	1.84 (1.22 to 2.72)	0.85 (0.53 to 1.26)	3.82 (2.48 to 4.85)	4.79 (3.28 to 6.55)	2.9 (1.46 to 4.16)	189.6 (77 to 346)	160.7 (49.7 to 351.4)	242 (91.2 to 513.3)
	Prevalence	9.42 (6.69 to 12.82)	13.85 (8.85 to 20.49)	5.54 (3.36 to 8.49)	32.29 (20.66 to 41.24)	41.26 (27.74 to 56.88)	23.72 (11.51 to 34.59)	242.7 (110.2 to 451.9)	197.9 (69 to 438.3)	328 (132.9 to 686.7)
	Deaths	0.38 (0.28 to 0.55)	0.44 (0.31 to 0.73)	0.31 (0.2 to 0.45)	0.46 (0.32 to 0.56)	0.45 (0.34 to 0.58)	0.47 (0.27 to 0.63)	19.3 (-21.3 to 74.9)	1.7 (-36.9 to 57.9)	48.9 (-13.5 to 153.9)
	DALYs	9.84 (7.46 to 12.94)	11.11 (7.93 to 16.27)	8.56 (5.3 to 12.56)	13.07 (8.49 to 16.08)	12.8 (9.44 to 16.5)	13.4 (7.18 to 17.88)	32.8 (-11.9 to 90.9)	15.2 (-28.4 to 79.5)	56.6 (-9.4 to 157.3)
	YLLs	9.23 (6.94 to 12.24)	10.25 (7.25 to 15.23)	8.17 (5.09 to 12.13)	11.17 (7.23 to 13.84)	10.42 (7.57 to 13.55)	11.95 (6.44 to 16.23)	21 (-20.4 to 73.8)	1.6 (-36.1 to 61.8)	46.2 (-17.2 to 145.1)
	YLDs	0.61 (0.35 to 0.95)	0.86 (0.45 to 1.39)	0.39 (0.21 to 0.67)	1.91 (1.11 to 2.86)	2.38 (1.36 to 3.8)	1.46 (0.67 to 2.35)	212.1 (92.9 to 401.5)	177.8 (53.9 to 415.1)	273.2 (99.4 to 604.2)
Kurdistan	Incidence	1 (0.74 to 1.56)	1.44 (0.99 to 2.47)	0.62 (0.41 to 0.88)	2.71 (2 to 3.33)	3.56 (2.55 to 4.63)	1.86 (1.28 to 2.5)	169.8 (48.4 to 303.5)	146.9 (28.3 to 306.3)	202.6 (67.9 to 413)
	Prevalence	6.76 (4.82 to 10.52)	10.35 (6.77 to 17.74)	3.58 (2.31 to 5.34)	22.32 (16.17 to 27.69)	30.09 (21.26 to 39.54)	14.6 (9.69 to 20.02)	230.1 (73.2 to 423.1)	190.9 (42.9 to 408.8)	307.8 (118.9 to 622.5)
	Deaths	0.34 (0.26 to 0.51)	0.42 (0.3 to 0.72)	0.28 (0.19 to 0.39)	0.41 (0.33 to 0.47)	0.43 (0.34 to 0.54)	0.38 (0.29 to 0.49)	19 (-23.4 to 65.7)	3.8 (-36.4 to 55.3)	37.7 (-15.1 to 114.5)
	DALYs	8.45 (6.55 to 12.15)	10.14 (7.33 to 16.91)	7.05 (4.92 to 10.03)	11.05 (8.46 to 12.91)	11.59 (8.88 to 14.44)	10.55 (7.6 to 13.15)	30.8 (-22.8 to 80.3)	14.2 (-36.7 to 71.2)	49.6 (-12 to 129.1)
	YLLs	8 (6.13 to 11.54)	9.49 (6.78 to 15.84)	6.78 (4.7 to 9.71)	9.72 (7.48 to 11.38)	9.84 (7.63 to 12.27)	9.63 (6.9 to 12.14)	21.5 (-28.5 to 68.2)	3.7 (-42.5 to 56.7)	42 (-17.3 to 118.4)
	YLDs	0.45 (0.27 to 0.76)	0.66 (0.36 to 1.16)	0.27 (0.15 to 0.44)	1.33 (0.81 to 1.99)	1.75 (1.01 to 2.75)	0.92 (0.52 to 1.43)	196.1 (55.5 to 374.8)	166.7 (31.8 to 391)	242.3 (79.4 to 508.7)

Province	Measure	Age-standardized rate (per 100,000)						% Change (1990 to 2019)		
		1990			2019					
		Both	Female	Male	Both	Female	Male	Both	Female	Male
Lorestan	Incidence	2.08 (1.34 to 2.81)	3.23 (1.89 to 4.64)	1.05 (0.67 to 1.5)	4.63 (2.44 to 6.03)	6.31 (2.91 to 8.86)	2.92 (1.77 to 4.02)	122.4 (49.8 to 230.9)	95.4 (18.3 to 224.2)	179.1 (61.8 to 363.7)
	Prevalence	14.82 (9.02 to 20.89)	24.13 (12.88 to 36.37)	6.55 (3.82 to 10.02)	38.77 (19.74 to 51.06)	54.24 (24.56 to 76.97)	23.06 (13.52 to 32.61)	161.6 (72.1 to 313.9)	124.8 (31.1 to 300.3)	251.9 (92.5 to 502.6)
	Deaths	0.61 (0.43 to 0.77)	0.81 (0.48 to 1.11)	0.42 (0.28 to 0.57)	0.59 (0.37 to 0.72)	0.62 (0.33 to 0.81)	0.57 (0.39 to 0.72)	-2.4 (-29.2 to 34.4)	-23.9 (-50.1 to 15.5)	35.9 (-12.6 to 114.9)
	DALYs	15.61 (10.56 to 19.77)	20.72 (11.89 to 27.74)	11.1 (7.38 to 15.41)	16.47 (9.43 to 20.33)	17.67 (8.51 to 23.63)	15.26 (9.73 to 19.8)	5.5 (-22.7 to 45.7)	-14.7 (-43.3 to 32.4)	37.4 (-14.7 to 119)
	YLLs	14.65 (9.98 to 18.66)	19.21 (10.98 to 26.18)	10.63 (6.97 to 14.77)	14.16 (8.29 to 17.43)	14.54 (7.17 to 19.48)	13.78 (8.75 to 18.06)	-3.3 (-30.4 to 36.1)	-24.3 (-51.4 to 18)	29.6 (-20.9 to 107.6)
	YLDs	0.96 (0.51 to 1.5)	1.51 (0.73 to 2.49)	0.47 (0.25 to 0.79)	2.31 (1.12 to 3.62)	3.13 (1.29 to 5.31)	1.47 (0.8 to 2.31)	141 (52.1 to 275.1)	107.8 (20.6 to 255.9)	214.9 (73.5 to 481.1)
Markazi	Incidence	1.35 (1.03 to 1.84)	1.98 (1.36 to 2.85)	0.72 (0.5 to 1.06)	4.26 (2.05 to 5.5)	5.96 (2.65 to 8.26)	2.57 (1.34 to 3.51)	216.7 (31.4 to 386.3)	201.9 (17.7 to 403.3)	257 (54.3 to 482.9)
	Prevalence	9.48 (6.83 to 13.26)	14.47 (9.36 to 21.64)	4.56 (3 to 6.96)	35.62 (16.72 to 46.19)	50.76 (22.17 to 70.79)	20.54 (10.27 to 28.48)	275.8 (49 to 504.8)	250.7 (31.4 to 529.6)	350.2 (94.1 to 669.1)
	Deaths	0.41 (0.31 to 0.55)	0.53 (0.38 to 0.77)	0.28 (0.2 to 0.4)	0.57 (0.34 to 0.7)	0.67 (0.35 to 0.88)	0.47 (0.3 to 0.61)	40.8 (-28.5 to 102.8)	26.3 (-41.2 to 91.4)	68.8 (-17.9 to 173.4)
	DALYs	10.07 (7.64 to 13.32)	12.71 (9.24 to 17.71)	7.47 (5.25 to 10.73)	15.78 (8.37 to 19.62)	18.15 (8.76 to 23.74)	13.33 (7.58 to 17.29)	56.7 (-27 to 128.6)	42.8 (-38 to 122.2)	78.5 (-18.1 to 192.9)
	YLLs	9.45 (7.12 to 12.59)	11.79 (8.55 to 16.6)	7.14 (4.98 to 10.32)	13.67 (7.44 to 16.93)	15.21 (7.45 to 20.03)	12.04 (6.89 to 15.81)	44.7 (-32 to 111.4)	29 (-44 to 105.1)	68.7 (-21.9 to 182.5)
	YLDs	0.62 (0.36 to 0.96)	0.92 (0.5 to 1.51)	0.33 (0.18 to 0.54)	2.11 (0.95 to 3.24)	2.94 (1.22 to 4.81)	1.29 (0.62 to 2.07)	239.8 (39 to 459.8)	219.9 (25.9 to 501.2)	292.1 (60.6 to 599.6)

Province	Measure	Age-standardized rate (per 100,000)						% Change (1990 to 2019)		
		1990			2019					
		Both	Female	Male	Both	Female	Male	Both	Female	Male
Mazandaran	Incidence	1.61 (1.2 to 2.17)	2.41 (1.65 to 3.44)	0.81 (0.54 to 1.17)	3.54 (2.68 to 4.36)	4.81 (3.43 to 6.32)	2.25 (1.58 to 3.04)	120.4 (50.2 to 221.9)	99.9 (24.1 to 221)	177.9 (60.4 to 365.1)
	Prevalence	12.38 (9.1 to 16.78)	19.22 (12.92 to 27.83)	5.73 (3.72 to 8.52)	30.06 (22.45 to 37.28)	41.57 (29.35 to 55.1)	18.42 (12.59 to 25.05)	142.7 (63.6 to 261.7)	116.3 (33.4 to 258)	221.4 (85.6 to 454.7)
	Deaths	0.34 (0.26 to 0.51)	0.43 (0.31 to 0.72)	0.25 (0.16 to 0.36)	0.39 (0.32 to 0.47)	0.43 (0.33 to 0.55)	0.36 (0.27 to 0.46)	14 (-20.5 to 61)	-1.3 (-35.7 to 49.9)	45.2 (-10.2 to 135.3)
	DALYs	8.78 (6.68 to 11.84)	11.04 (8.07 to 16.22)	6.46 (4.4 to 9.18)	11.05 (9.02 to 13.12)	12.16 (9.24 to 15.25)	9.9 (7.57 to 12.57)	25.9 (-11.4 to 72.4)	10.2 (-26.2 to 66.3)	53.2 (-7.4 to 146.8)
	YLLs	8.01 (6.11 to 10.9)	9.87 (7.08 to 14.71)	6.07 (4.1 to 8.72)	9.28 (7.56 to 11.07)	9.78 (7.43 to 12.53)	8.76 (6.63 to 11.13)	16 (-19.1 to 63)	-1 (-34.7 to 51.8)	44.1 (-12.7 to 134.5)
	YLDs	0.77 (0.47 to 1.21)	1.16 (0.65 to 1.91)	0.39 (0.21 to 0.67)	1.77 (1.03 to 2.59)	2.38 (1.37 to 3.66)	1.14 (0.64 to 1.76)	128.8 (50.3 to 247.1)	104.5 (22.5 to 253.3)	196.2 (61.8 to 428.3)
North Khorasan	Incidence	1.18 (0.87 to 1.58)	1.78 (1.23 to 2.55)	0.61 (0.41 to 0.89)	3.44 (2.31 to 4.2)	4.94 (3.17 to 6.34)	1.91 (1.31 to 2.5)	190.8 (70.3 to 338.1)	176.8 (53.9 to 345.8)	212.5 (75.5 to 403.7)
	Prevalence	7.69 (5.36 to 10.77)	12.26 (7.69 to 18.27)	3.38 (2.18 to 5.13)	28.04 (18.24 to 34.69)	41.11 (25.74 to 53.78)	14.63 (9.73 to 19.5)	264.8 (110.3 to 477)	235.5 (83.3 to 492.1)	332.6 (134.8 to 661.7)
	Deaths	0.43 (0.34 to 0.58)	0.58 (0.43 to 0.82)	0.29 (0.21 to 0.43)	0.53 (0.42 to 0.63)	0.64 (0.47 to 0.79)	0.43 (0.33 to 0.53)	23.1 (-18.4 to 75.7)	11.7 (-31.9 to 68.1)	45.6 (-13.2 to 124.4)
	DALYs	11.06 (8.58 to 14.1)	14.46 (10.3 to 20.07)	7.91 (5.56 to 11.35)	14.59 (10.59 to 17.3)	17.42 (11.59 to 21.63)	11.73 (8.47 to 14.49)	31.8 (-15.2 to 87.8)	20.5 (-28.2 to 85.4)	48.2 (-14.1 to 129.9)
	YLLs	10.54 (8.13 to 13.43)	13.65 (9.8 to 18.83)	7.65 (5.35 to 11.07)	12.88 (9.41 to 15.31)	14.97 (10.01 to 18.51)	10.79 (7.88 to 13.39)	22.3 (-20.4 to 72.6)	9.7 (-34 to 67.2)	40.9 (-19 to 122.3)
	YLDs	0.53 (0.32 to 0.83)	0.81 (0.45 to 1.35)	0.26 (0.15 to 0.42)	1.7 (0.94 to 2.51)	2.45 (1.27 to 3.79)	0.94 (0.54 to 1.46)	223.7 (84.9 to 412.4)	202.9 (57.6 to 445.1)	260.7 (91.6 to 511.6)

Province	Measure	Age-standardized rate (per 100,000)						% Change (1990 to 2019)		
		1990			2019					
		Both	Female	Male	Both	Female	Male	Both	Female	Male
Qazvin	Incidence	1.11 (0.78 to 1.46)	1.81 (1.19 to 2.47)	0.43 (0.28 to 0.71)	3.24 (2.03 to 4.16)	5.02 (2.54 to 6.78)	1.5 (1.14 to 1.98)	192.8 (62.4 to 366.3)	176.8 (36.3 to 380.3)	251.2 (106.6 to 486.2)
	Prevalence	7.57 (4.94 to 10.25)	12.96 (8.12 to 18.33)	2.52 (1.57 to 4.09)	26.97 (16.09 to 35.02)	42.83 (21.26 to 58.39)	11.55 (8.42 to 15.65)	256.5 (96.3 to 488.6)	230.6 (59.2 to 504.4)	359 (159.8 to 695.9)
	Deaths	0.36 (0.27 to 0.51)	0.52 (0.37 to 0.78)	0.19 (0.12 to 0.33)	0.44 (0.34 to 0.53)	0.53 (0.32 to 0.69)	0.33 (0.26 to 0.43)	21.4 (-21.8 to 76.9)	2.8 (-41.9 to 61)	74.3 (12.4 to 182.5)
	DALYs	8.64 (6.6 to 11.43)	12.58 (9.09 to 17.16)	4.82 (3.18 to 7.89)	11.59 (8.43 to 14.17)	14.74 (7.94 to 19.17)	8.39 (6.74 to 10.59)	34.2 (-16.1 to 93.6)	17.2 (-37.4 to 78.4)	74.3 (9 to 179.8)
	YLLs	8.13 (6.18 to 10.86)	11.74 (8.27 to 16.17)	4.63 (3.03 to 7.67)	9.98 (7.49 to 12.27)	12.25 (6.83 to 16.09)	7.65 (6.02 to 9.73)	22.8 (-22.3 to 80.3)	4.4 (-43.9 to 60.7)	65.4 (4.7 to 170.7)
	YLDs	0.51 (0.3 to 0.77)	0.84 (0.48 to 1.31)	0.19 (0.11 to 0.31)	1.61 (0.86 to 2.49)	2.49 (1.1 to 4.1)	0.74 (0.44 to 1.16)	218 (76.1 to 421.1)	197.3 (48.1 to 454.1)	289.6 (106.9 to 596.4)
Qom	Incidence	1.8 (1.06 to 2.37)	2.89 (1.5 to 3.98)	0.77 (0.48 to 1.1)	3.89 (1.86 to 4.97)	5.97 (2.49 to 8.07)	1.93 (1.11 to 2.6)	116.3 (43 to 241.3)	106.6 (27.7 to 247.1)	151.5 (41.3 to 348.4)
	Prevalence	12.05 (6.43 to 16.49)	20.27 (9.58 to 29.2)	4.54 (2.56 to 6.83)	31.83 (14.8 to 41.09)	49.46 (20.01 to 67.8)	15.03 (8.48 to 20.76)	164.2 (71.9 to 351.3)	144 (45.3 to 334.5)	231.4 (83.9 to 547.7)
	Deaths	0.61 (0.42 to 0.8)	0.87 (0.53 to 1.21)	0.34 (0.22 to 0.46)	0.59 (0.34 to 0.73)	0.81 (0.39 to 1.04)	0.4 (0.27 to 0.52)	-3.6 (-31.7 to 37)	-7.2 (-40.7 to 43.3)	18.3 (-25.6 to 90.2)
	DALYs	14.81 (9.25 to 18.77)	20.89 (11.23 to 27.92)	8.87 (5.62 to 12.15)	15.23 (7.93 to 18.73)	20.19 (9.16 to 26.47)	10.65 (6.7 to 13.84)	2.8 (-28.3 to 53.6)	-3.4 (-39.3 to 53.2)	20.1 (-25.3 to 97.7)
	YLLs	13.99 (8.79 to 17.84)	19.57 (10.45 to 26.25)	8.53 (5.35 to 11.71)	13.3 (7.14 to 16.55)	17.24 (7.81 to 22.61)	9.69 (6.18 to 12.76)	-5 (-33.4 to 45.1)	-11.9 (-44.8 to 41.9)	13.6 (-29.9 to 88.8)
	YLDs	0.81 (0.4 to 1.24)	1.32 (0.6 to 2.08)	0.34 (0.17 to 0.55)	1.93 (0.84 to 3.02)	2.95 (1.13 to 4.73)	0.96 (0.5 to 1.53)	137.7 (50.8 to 288.6)	123.2 (30.3 to 299.4)	185.3 (51 to 457.8)

Province	Measure	Age-standardized rate (per 100,000)						% Change (1990 to 2019)		
		1990			2019					
		Both	Female	Male	Both	Female	Male	Both	Female	Male
Semnan	Incidence	1.11 (0.79 to 1.73)	1.57 (1.02 to 2.67)	0.64 (0.43 to 0.95)	3.31 (2.31 to 4.19)	4.09 (2.88 to 5.52)	2.55 (1.57 to 3.41)	199.2 (57.8 to 370.6)	160.8 (32 to 350.4)	295.8 (91.5 to 579.5)
	Prevalence	7.61 (5.18 to 11.97)	11.37 (7.25 to 19.16)	3.99 (2.5 to 6.04)	27.41 (18.61 to 35.03)	34.95 (24.26 to 47.36)	19.99 (11.95 to 27.45)	260 (81.2 to 497.5)	207.4 (49.8 to 457.6)	401.2 (134.7 to 804.2)
	Deaths	0.35 (0.25 to 0.58)	0.43 (0.28 to 0.81)	0.26 (0.17 to 0.38)	0.47 (0.36 to 0.57)	0.42 (0.32 to 0.54)	0.52 (0.36 to 0.66)	33.2 (-19.2 to 99.3)	-2.4 (-46.1 to 57.1)	100.8 (6.5 to 224.6)
	DALYs	8.4 (6.08 to 12.9)	10.1 (6.82 to 18.05)	6.59 (4.4 to 9.75)	12.46 (8.9 to 14.91)	11.5 (8.35 to 14.85)	13.43 (8.8 to 16.8)	48.3 (-17.2 to 119.9)	13.9 (-37.6 to 82.3)	103.8 (3.9 to 229)
	YLLs	7.89 (5.66 to 12.16)	9.37 (6.3 to 16.77)	6.29 (4.19 to 9.39)	10.82 (7.71 to 13.05)	9.49 (7.09 to 12.09)	12.15 (7.98 to 15.2)	37.1 (-22.3 to 104.9)	1.3 (-44.9 to 63.1)	93.1 (-1.3 to 214.7)
	YLDs	0.51 (0.29 to 0.85)	0.73 (0.38 to 1.36)	0.29 (0.16 to 0.47)	1.64 (0.98 to 2.48)	2.01 (1.17 to 3.22)	1.27 (0.67 to 1.99)	221.7 (61.4 to 447.3)	175.9 (37.5 to 407.2)	333.8 (110.2 to 702.6)
Sistan and Baluchistan	Incidence	0.84 (0.56 to 1.2)	1.16 (0.71 to 1.9)	0.58 (0.33 to 0.84)	3.9 (1.92 to 4.99)	5.06 (2.57 to 6.89)	2.75 (1 to 3.67)	362.5 (85.2 to 651.8)	335.3 (59.5 to 709.7)	376.9 (85.7 to 786.1)
	Prevalence	4.84 (2.73 to 7.07)	7.35 (3.95 to 11.8)	2.75 (1.37 to 4.23)	30.83 (14.78 to 40.09)	41.59 (20.53 to 57.32)	20.14 (6.96 to 27.53)	537.2 (145.7 to 1060.9)	465.7 (104.9 to 1062.4)	632.4 (175.9 to 1384.5)
	Deaths	0.39 (0.27 to 0.58)	0.46 (0.31 to 0.84)	0.33 (0.21 to 0.47)	0.75 (0.39 to 0.92)	0.75 (0.46 to 0.98)	0.74 (0.31 to 0.99)	92 (-11.2 to 191.4)	64.7 (-33.1 to 165.8)	125 (-3.2 to 288.7)
	DALYs	9.58 (6.41 to 13.41)	11.05 (7.2 to 19.08)	8.39 (4.81 to 12.29)	21.3 (10.37 to 26.61)	21.73 (12.12 to 28.38)	20.9 (8.14 to 27.43)	122.4 (-3.8 to 251.6)	96.7 (-26.2 to 224.4)	149.2 (2.6 to 343.9)
	YLLs	9.22 (6.19 to 12.96)	10.54 (6.84 to 18.27)	8.15 (4.6 to 11.98)	19.38 (9.57 to 24.21)	19.22 (10.78 to 25.51)	19.57 (7.68 to 26.14)	110.2 (-8.8 to 234.5)	82.3 (-31.1 to 206.6)	140 (-1.6 to 331.2)
	YLDs	0.36 (0.18 to 0.57)	0.51 (0.26 to 0.9)	0.23 (0.12 to 0.38)	1.92 (0.82 to 2.9)	2.51 (1.06 to 4)	1.33 (0.46 to 2.13)	436.6 (113.9 to 873.4)	395.7 (74.2 to 897.1)	469.4 (114.6 to 1022.8)

Province	Measure	Age-standardized rate (per 100,000)						% Change (1990 to 2019)		
		1990			2019					
		**Both**	**Female**	**Male**	**Both**	**Female**	**Male**	**Both**	**Female**	**Male**
**South Khorasan**	**Incidence**	1.58 (1.14 to 2.05)	2.34 (1.53 to 3.22)	0.88 (0.55 to 1.25)	3.84 (1.95 to 4.81)	5.2 (2.63 to 6.93)	2.43 (1.13 to 3.31)	142.4 (57 to 244.9)	122.4 (33.5 to 247.5)	177.4 (60.6 to 362.6)
	**Prevalence**	10.94 (7.58 to 14.88)	17.05 (10.72 to 24.62)	5.27 (3.04 to 7.79)	31.64 (15.68 to 40.11)	43.93 (21.51 to 59.28)	18.93 (8.4 to 26)	189.1 (81.3 to 325.5)	157.6 (50.2 to 330.4)	259.2 (106.1 to 512.6)
	**Deaths**	0.51 (0.38 to 0.64)	0.65 (0.47 to 0.88)	0.38 (0.24 to 0.54)	0.58 (0.35 to 0.69)	0.63 (0.38 to 0.79)	0.52 (0.29 to 0.68)	13.6 (-19.8 to 53.8)	-2.5 (-37.6 to 40.6)	38.7 (-16.1 to 119.9)
	**DALYs**	12.95 (9.5 to 16.05)	16.17 (11.46 to 21)	9.97 (6.29 to 14.4)	15.52 (8.43 to 18.68)	17.05 (9.19 to 21.5)	13.91 (7.04 to 17.84)	19.9 (-18.3 to 63.8)	5.5 (-32.3 to 54.5)	39.6 (-16.6 to 120.6)
	**YLLs**	12.23 (9.04 to 15.25)	15.09 (10.58 to 19.9)	9.59 (5.98 to 13.98)	13.64 (7.49 to 16.37)	14.5 (8.07 to 18.19)	12.72 (6.44 to 16.49)	11.5 (-23.9 to 53.6)	-3.9 (-38.5 to 40.1)	32.7 (-21.4 to 110.5)
	**YLDs**	0.72 (0.41 to 1.1)	1.08 (0.58 to 1.74)	0.38 (0.21 to 0.65)	1.88 (0.86 to 2.87)	2.55 (1.12 to 4.01)	1.19 (0.51 to 1.87)	162.6 (65.1 to 301.8)	137.3 (34.9 to 293.8)	210.6 (69.2 to 449)
**Tehran**	**Incidence**	3.14 (1.48 to 4.33)	5.04 (2.31 to 7.32)	1.35 (0.66 to 2.14)	3.4 (1.78 to 4.32)	4.85 (2.5 to 6.56)	1.96 (1.01 to 2.66)	8.1 (-23.7 to 55.2)	-3.7 (-36.7 to 49.9)	45.3 (-15.6 to 138.7)
	**Prevalence**	24.67 (11.39 to 34.49)	40.75 (18.39 to 60.57)	9.77 (4.52 to 15.72)	28.4 (14.67 to 36.31)	41.13 (20.84 to 56.41)	15.72 (7.9 to 21.56)	15.1 (-21.1 to 68.7)	0.9 (-35 to 61.6)	60.8 (-9.7 to 174.6)
	**Deaths**	0.63 (0.33 to 0.88)	0.87 (0.42 to 1.24)	0.38 (0.21 to 0.58)	0.43 (0.25 to 0.52)	0.53 (0.29 to 0.67)	0.33 (0.19 to 0.44)	-32.3 (-52 to -6.4)	-39 (-58.1 to -10.8)	-11.2 (-47 to 45.8)
	**DALYs**	15.79 (7.69 to 21.3)	21.7 (10.01 to 30.27)	9.81 (5.21 to 15.17)	11.31 (6.14 to 13.7)	13.65 (7.19 to 17.27)	9.01 (4.84 to 11.9)	-28.4 (-47 to -2.1)	-37.1 (-55.5 to -10.1)	-8.2 (-45 to 47.5)
	**YLLs**	14.28 (7.06 to 19.66)	19.28 (8.94 to 27.71)	9.17 (4.8 to 14.33)	9.61 (5.33 to 11.74)	11.23 (5.77 to 14.37)	8.02 (4.3 to 10.58)	-32.7 (-51.5 to -7.4)	-41.7 (-60.1 to -14.4)	-12.5 (-48.7 to 42.6)
	**YLDs**	1.5 (0.66 to 2.41)	2.42 (1 to 4.09)	0.64 (0.27 to 1.17)	1.7 (0.84 to 2.56)	2.41 (1.1 to 3.77)	0.99 (0.47 to 1.56)	13 (-22.5 to 68.2)	-0.3 (-37.3 to 63.2)	54.2 (-14.7 to 176.4)

Province	Measure	Age-standardized rate (per 100,000)						% Change (1990 to 2019)		
		1990			2019					
		Both	Female	Male	Both	Female	Male	Both	Female	Male
West Azarbayejan	Incidence	1.02 (0.74 to 1.58)	1.51 (1.03 to 2.54)	0.56 (0.4 to 0.82)	3.09 (2.14 to 3.86)	4.43 (2.89 to 5.75)	1.75 (1.2 to 2.28)	202.6 (52.8 to 345.9)	193.1 (35.4 to 379.3)	211.1 (68.4 to 402.4)
	Prevalence	6.69 (4.66 to 10.57)	10.5 (6.74 to 17.42)	3.17 (2.09 to 4.76)	25.2 (16.92 to 31.81)	37.11 (23.43 to 48.93)	13.28 (8.67 to 17.75)	276.8 (80 to 486.9)	253.6 (55.5 to 508.6)	318.4 (118.6 to 610.6)
	Deaths	0.37 (0.28 to 0.57)	0.48 (0.34 to 0.84)	0.26 (0.19 to 0.39)	0.49 (0.41 to 0.58)	0.57 (0.44 to 0.7)	0.4 (0.31 to 0.5)	32.2 (-19.2 to 88.2)	19.2 (-35.9 to 84.8)	52.7 (-6.7 to 130.9)
	DALYs	8.74 (6.76 to 13.12)	11.06 (8.02 to 18.84)	6.58 (4.68 to 9.5)	12.52 (9.66 to 14.79)	14.63 (10.51 to 18.15)	10.31 (7.91 to 12.95)	43.3 (-18.6 to 101.1)	32.2 (-32.4 to 101.4)	56.8 (-5.8 to 135.1)
	YLLs	8.28 (6.4 to 12.39)	10.38 (7.42 to 17.52)	6.33 (4.46 to 9.19)	11.01 (8.49 to 12.98)	12.46 (9.15 to 15.45)	9.45 (7.22 to 12.02)	32.9 (-23.4 to 88.9)	20 (-38.1 to 85.2)	49.3 (-10.6 to 124.6)
	YLDs	0.46 (0.27 to 0.75)	0.68 (0.37 to 1.23)	0.24 (0.14 to 0.4)	1.52 (0.9 to 2.27)	2.17 (1.18 to 3.38)	0.86 (0.51 to 1.32)	232.1 (57.1 to 418.1)	216.9 (39 to 449)	250.7 (82.3 to 510.8)
Yazd	Incidence	1.21 (0.89 to 1.68)	1.83 (1.26 to 2.69)	0.58 (0.39 to 0.88)	4.13 (2.45 to 5.3)	6.15 (3.2 to 8.45)	2.22 (1.45 to 3.04)	241.2 (66.8 to 433.9)	236.3 (45 to 490.8)	279.8 (94.8 to 561)
	Prevalence	8.29 (5.81 to 11.77)	13.3 (8.73 to 19.67)	3.51 (2.27 to 5.4)	34.57 (20.07 to 44.65)	52.57 (26.9 to 73.03)	17.71 (11.22 to 24.58)	317.2 (94.7 to 578.2)	295.1 (66.9 to 613.2)	404.2 (157.2 to 823.5)
	Deaths	0.39 (0.29 to 0.58)	0.5 (0.33 to 0.8)	0.25 (0.17 to 0.37)	0.53 (0.37 to 0.64)	0.64 (0.39 to 0.84)	0.41 (0.3 to 0.54)	34.9 (-21.3 to 102.2)	27.7 (-36.3 to 113.5)	65 (-2 to 165.5)
	DALYs	9.17 (6.78 to 12.96)	11.84 (8.22 to 18.05)	6.2 (4.29 to 9.11)	13.76 (8.86 to 16.81)	16.89 (9.19 to 22.26)	10.69 (7.7 to 13.85)	50.1 (-18.4 to 121.3)	42.6 (-32.7 to 126.7)	72.5 (-1.5 to 178.3)
	YLLs	8.62 (6.38 to 12.17)	11 (7.59 to 16.97)	5.94 (4.08 to 8.81)	11.72 (7.76 to 14.29)	13.87 (7.69 to 18.34)	9.58 (6.86 to 12.36)	36.1 (-25.1 to 102.7)	26.1 (-40 to 109)	61.4 (-7.5 to 166.8)
	YLDs	0.55 (0.32 to 0.86)	0.84 (0.47 to 1.39)	0.26 (0.14 to 0.44)	2.04 (1.07 to 3.1)	3.03 (1.38 to 4.75)	1.11 (0.62 to 1.78)	270.3 (77.2 to 502.1)	258.3 (52 to 569.1)	323.5 (107.3 to 701.6)

Province	Measure	Age-standardized rate (per 100,000)						% Change (1990 to 2019)		
		1990			2019					
		Both	Female	Male	Both	Female	Male	Both	Female	Male
Zanjan	Incidence	0.74 (0.54 to 1.31)	1.07 (0.73 to 2.07)	0.42 (0.28 to 0.7)	2.26 (1.77 to 2.79)	3 (2.16 to 4.01)	1.49 (1.1 to 1.94)	206.2 (53.6 to 357.9)	180.4 (35.2 to 347.4)	258.7 (88.2 to 486.9)
	Prevalence	4.89 (3.44 to 8.83)	7.51 (4.97 to 14.58)	2.46 (1.57 to 4.09)	18.42 (13.94 to 23.16)	25.31 (17.63 to 34.05)	11.39 (8.03 to 15.14)	276.9 (81.3 to 489.5)	237.2 (54.1 to 466)	362.4 (142.3 to 710.9)
	Deaths	0.26 (0.18 to 0.46)	0.33 (0.21 to 0.68)	0.18 (0.12 to 0.34)	0.36 (0.3 to 0.43)	0.37 (0.29 to 0.47)	0.35 (0.27 to 0.43)	39.1 (-17.3 to 103.7)	13.3 (-37 to 78.7)	89.1 (6.2 to 207.6)
	DALYs	6.1 (4.49 to 10.48)	7.62 (5.14 to 14.92)	4.64 (3.17 to 8.07)	9.17 (7.62 to 10.92)	9.62 (7.41 to 11.98)	8.7 (6.71 to 10.8)	50.3 (-16.4 to 113.3)	26.2 (-32.6 to 91.2)	87.4 (0.4 to 199.9)
	YLLs	5.77 (4.23 to 9.84)	7.13 (4.77 to 13.94)	4.46 (3.01 to 7.72)	8.06 (6.74 to 9.57)	8.14 (6.32 to 10.3)	7.96 (6.14 to 9.85)	39.8 (-21.6 to 100.4)	14.3 (-39 to 75.7)	78.6 (-3.5 to 189.6)
	YLDs	0.33 (0.2 to 0.6)	0.49 (0.27 to 0.94)	0.19 (0.11 to 0.32)	1.11 (0.68 to 1.65)	1.47 (0.83 to 2.28)	0.74 (0.44 to 1.15)	232.7 (59.6 to 430.2)	200.4 (37.8 to 424.4)	296.3 (101.1 to 595.6)

*Data in parentheses are 95% Uncertainty Intervals (95% UIs).
