# Supplementary material for: National, sub-national, and risk-attributed burden of thyroid cancer in Iran from 1990 to 2019
Source: Sci Rep. 2022 Aug 2;12:13231. doi: 10.1038/s41598-022-17115-0 (PMC9346133; doi:10.1038/s41598-022-17115-0)
Supplement: Supplementary file 6 — Supplementary Table 2. [file 41598_2022_17115_MOESM6_ESM.pdf]

Location	Measure	% Change (1990 to 2000)			% Change (2000 to 2010)			% Change (2010 to 2019)		
		Both	Female	Male	Both	Female	Male	Both	Female	Male
Iran	Incidence	19.1	16.4	27.2	35.7	30.9	43.2	42.7	39.3	51.4
	Prevalence	30.1	25.4	43.2	38.5	32.6	48.9	46.5	42.3	58.5
	Deaths	-18.4	-23.2	-5.9	21.5	19.2	24.4	16.0	11.9	21.2
	DALYs	-13.2	-17.7	-4.1	24.7	21.0	28.3	16.8	13.1	21.5
	YLLs	-16.0	-21.1	-6.0	23.3	19.5	27.1	13.3	8.8	18.8
	YLDs	23.4	19.8	33.5	36.7	31.5	44.9	45.0	40.9	55.3
Alborz	Incidence	16.9	15.4	26.4	5.7	-4.2	28.3	60.8	49.8	84.5
	Prevalence	25.1	22.1	39.3	8.8	-2.5	36.2	63.9	52.4	90.1
	Deaths	-17.9	-21.5	-6.0	-11.2	-15.2	-2.6	34.1	22.4	52.0
	DALYs	-13.6	-16.8	-4.3	-9.6	-17.3	1.9	35.1	23.6	51.0
	YLLs	-16.7	-20.6	-6.4	-11.7	-19.3	-0.4	30.9	18.5	47.2
	YLDs	20.3	18.2	31.6	6.2	-4.6	31.1	62.2	50.8	86.5
Ardebil	Incidence	36.4	33.1	38.2	24.9	16.7	48.6	56.9	53.1	68.8
	Prevalence	54.5	48.4	64.2	25.0	17.4	51.1	60.8	56.5	76.6
	Deaths	-18.1	-23.4	-6.4	22.0	10.0	37.8	29.5	20.0	41.0
	DALYs	-12.9	-17.6	-5.6	18.1	7.0	34.9	31.2	24.1	41.0
	YLLs	-16.6	-22.0	-7.9	17.3	5.7	33.8	27.7	19.3	38.6
	YLDs	44.2	40.0	47.9	25.3	16.8	51.3	60.6	56.6	73.0
Bushehr	Incidence	22.3	18.7	35.5	99.1	119.8	59.4	40.6	37.0	43.4
	Prevalence	35.0	30.1	52.4	106.6	126.6	65.7	45.3	41.1	50.9
	Deaths	-16.7	-23.5	2.3	66.1	81.4	42.6	7.4	1.0	16.0
	DALYs	-12.4	-18.6	2.0	71.2	90.2	43.2	9.5	5.3	14.5
	YLLs	-15.2	-22.1	0.1	68.2	86.3	42.1	5.3	0.2	11.9
	YLDs	27.7	23.4	42.6	99.7	119.9	60.4	42.8	38.8	47.3
Chahar Mahaal and Bakhtiari	Incidence	23.1	21.1	23.8	66.5	56.6	85.4	11.3	6.0	23.9
	Prevalence	34.1	30.4	35.5	71.2	60.6	93.7	15.6	9.5	31.7
	Deaths	-13.7	-18.8	-2.5	42.3	29.3	58.0	-18.7	-28.6	-7.7
	DALYs	-9.9	-13.8	-3.6	45.5	33.1	60.1	-14.5	-22.5	-5.1
	YLLs	-12.7	-17.4	-5.3	42.9	29.6	58.0	-18.1	-27.5	-7.9
	YLDs	27.4	24.7	28.9	69.0	58.5	89.1	13.3	6.9	28.1

Location	Measure	% Change (1990 to 2000)			% Change (2000 to 2010)			% Change (2010 to 2019)		
		Both	Female	Male	Both	Female	Male	Both	Female	Male
East Azarbayejan	Incidence	16.3	14.1	20.1	108.4	105.8	108.6	55.8	50.9	67.7
	Prevalence	29.4	25.2	36.9	119.6	114.9	125.7	61.5	55.3	79.0
	Deaths	-17.0	-21.8	-7.0	62.0	56.0	67.6	22.4	18.1	26.7
	DALYs	-13.4	-17.9	-6.3	68.7	65.2	70.9	23.5	18.9	28.9
	YLLs	-15.5	-20.6	-7.7	64.9	60.4	68.4	19.5	14.1	25.7
	YLDs	22.0	19.0	26.9	112.6	109.6	113.6	58.5	52.5	73.4
Fars	Incidence	24.9	21.5	34.6	73.5	75.9	61.9	37.3	32.9	50.0
	Prevalence	36.3	30.8	50.6	80.9	80.5	73.3	40.7	35.6	56.8
	Deaths	-13.2	-19.6	0.5	34.2	41.3	25.8	8.4	3.8	14.6
	DALYs	-8.0	-13.9	2.6	44.2	49.8	35.8	9.1	4.9	14.9
	YLLs	-10.7	-17.4	0.6	40.7	45.8	33.7	4.9	-0.1	11.5
	YLDs	29.2	24.7	41.0	76.4	78.7	64.5	39.6	34.5	54.0
Gilan	Incidence	22.9	19.8	34.5	56.1	54.4	57.0	40.0	38.8	44.8
	Prevalence	43.6	36.5	67.6	60.8	56.7	67.0	43.0	41.4	49.2
	Deaths	-15.5	-20.7	-1.1	28.7	35.3	20.6	16.6	14.0	21.8
	DALYs	-11.5	-16.7	0.0	34.8	37.5	30.4	17.2	15.7	19.9
	YLLs	-13.9	-19.9	-1.6	32.2	34.9	28.2	13.9	11.6	17.3
	YLDs	17.2	14.0	27.7	57.5	54.8	60.4	41.3	39.7	46.8
Golestan	Incidence	34.6	30.6	41.3	68.5	59.9	84.5	60.2	60.1	60.1
	Prevalence	54.0	46.8	67.9	76.3	63.6	105.2	65.7	66.2	64.8
	Deaths	-9.8	-18.2	4.0	38.2	39.8	36.6	28.5	17.4	41.1
	DALYs	-5.6	-12.9	4.7	44.7	38.8	51.4	30.9	26.9	35.2
	YLLs	-8.3	-16.3	2.9	42.4	36.4	49.1	27.6	22.1	33.1
	YLDs	42.8	37.6	51.3	71.4	60.6	92.6	63.9	63.9	63.6
Hamadan	Incidence	13.7	10.7	19.1	80.4	67.8	104.0	50.1	44.5	60.9
	Prevalence	25.6	20.3	34.7	85.8	72.4	113.4	53.5	47.5	66.5
	Deaths	-23.1	-26.7	-14.6	50.0	37.0	66.8	26.7	18.6	34.1
	DALYs	-18.6	-22.5	-12.4	58.1	43.5	75.3	25.6	19.0	31.6
	YLLs	-21.1	-25.5	-14.2	55.6	40.3	73.1	22.4	14.9	28.9
	YLDs	18.9	14.9	25.4	82.6	69.3	107.7	52.5	45.9	65.1

Location	Measure	% Change (1990 to 2000)			% Change (2000 to 2010)			% Change (2010 to 2019)		
		Both	Female	Male	Both	Female	Male	Both	Female	Male
Hormozgan	Incidence	27.5	27.8	27.3	59.5	44.8	81.3	46.1	36.0	62.0
	Prevalence	49.8	46.9	53.3	73.7	52.0	112.5	53.5	42.5	72.4
	Deaths	-11.7	-16.9	-3.4	17.9	15.0	21.3	8.1	-4.9	23.0
	DALYs	-8.6	-12.5	-3.2	24.5	14.8	34.4	11.2	-0.8	22.9
	YLLs	-10.6	-15.2	-4.5	21.6	11.9	31.6	7.7	-5.3	19.9
	YLDs	36.6	36.0	37.3	65.6	48.0	92.6	49.0	37.5	67.0
Ilam	Incidence	28.6	22.6	35.3	83.5	66.9	109.4	45.3	43.3	46.3
	Prevalence	44.3	35.9	54.6	93.2	72.7	131.1	48.6	47.3	49.3
	Deaths	-13.9	-22.6	-0.1	41.5	34.6	47.8	20.4	8.4	30.3
	DALYs	-9.5	-17.1	0.1	50.7	38.8	62.5	20.6	14.7	24.9
	YLLs	-12.4	-20.6	-1.9	47.2	35.1	59.0	17.3	10.1	22.7
	YLDs	35.0	28.5	41.9	86.2	68.3	115.1	46.9	44.5	48.8
Isfahan	Incidence	21.8	19.1	32.5	63.1	65.6	52.5	41.2	38.3	48.3
	Prevalence	31.1	26.8	45.0	67.7	68.1	59.9	44.3	40.7	53.4
	Deaths	-14.4	-19.4	0.8	36.4	45.3	25.3	18.1	15.5	23.0
	DALYs	-8.5	-13.4	2.9	42.7	49.4	32.5	17.8	15.2	21.7
	YLLs	-11.4	-17.1	0.9	40.2	47.0	30.9	14.2	11.0	18.9
	YLDs	26.1	22.7	37.9	63.4	65.7	52.7	43.5	39.9	52.1
Kerman	Incidence	29.6	26.7	37.1	96.0	100.1	79.5	27.9	28.2	30.5
	Prevalence	46.8	41.1	60.9	109.1	109.3	98.1	30.0	29.7	35.3
	Deaths	-11.8	-18.2	0.9	48.5	56.5	38.0	9.5	8.5	10.4
	DALYs	-7.5	-13.5	2.3	55.9	62.8	45.6	9.0	8.6	9.6
	YLLs	-10.0	-16.7	0.6	52.2	58.5	43.4	6.5	5.5	7.7
	YLDs	35.9	31.6	46.1	97.9	100.8	83.1	30.3	30.2	33.8
Kermanshah	Incidence	26.2	23.1	29.4	100.6	95.8	100.2	39.8	37.1	45.2
	Prevalence	44.1	37.7	53.8	114.1	104.4	123.2	43.7	40.8	51.0
	Deaths	-15.8	-21.3	-7.0	50.0	53.8	43.5	13.9	7.7	20.4
	DALYs	-11.7	-17.0	-5.0	63.1	63.3	59.7	12.8	8.8	17.1
	YLLs	-14.2	-20.1	-6.7	59.5	59.4	56.9	9.6	4.6	14.7
	YLDs	33.5	28.9	39.2	105.0	98.3	107.4	42.0	39.2	47.6

Location	Measure	% Change (1990 to 2000)			% Change (2000 to 2010)			% Change (2010 to 2019)		
		Both	Female	Male	Both	Female	Male	Both	Female	Male
Khorasan-e-Razavi	Incidence	22.2	19.7	25.9	59.0	59.6	52.0	55.8	55.6	57.4
	Prevalence	38.4	33.4	47.5	68.0	66.2	65.6	61.6	60.3	67.2
	Deaths	-16.0	-21.5	-6.0	26.9	28.8	22.4	20.2	18.3	22.3
	DALYs	-12.5	-17.4	-5.3	32.5	34.4	27.7	19.8	18.7	21.1
	YLLs	-14.8	-20.3	-6.9	30.0	31.5	26.2	15.7	13.6	18.2
	YLDs	28.6	24.7	35.2	61.4	61.8	54.3	58.8	57.5	63.4
Khuzestan	Incidence	17.4	15.8	28.8	58.1	56.2	52.6	56.6	57.0	54.9
	Prevalence	28.0	24.4	44.3	65.1	61.5	62.9	61.9	61.4	64.9
	Deaths	-14.5	-17.3	0.7	26.4	26.4	25.5	20.7	20.7	17.4
	DALYs	-10.0	-13.6	2.2	32.4	31.7	29.4	22.1	22.9	18.9
	YLLs	-12.2	-16.2	0.8	29.7	28.7	27.8	17.7	17.8	15.8
	YLDs	22.0	19.6	34.5	60.7	58.3	55.7	59.7	59.3	61.0
Kohgiluyeh and Boyer-Ahmad	Incidence	38.5	38.7	35.6	41.2	36.6	47.2	48.1	37.5	71.3
	Prevalence	55.3	52.2	55.6	46.0	40.3	54.0	51.1	39.5	78.6
	Deaths	-14.9	-18.2	-7.4	12.4	6.9	20.4	24.8	16.4	33.5
	DALYs	-7.0	-8.9	-3.5	15.3	10.7	21.0	23.9	14.3	34.2
	YLLs	-10.5	-13.5	-5.7	12.3	6.8	18.8	20.3	9.9	30.5
	YLDs	45.5	45.4	42.2	43.1	38.0	50.2	49.8	38.4	74.7
Kurdistan	Incidence	11.5	10.4	10.4	68.2	60.1	78.9	43.9	39.7	53.2
	Prevalence	23.5	20.8	25.1	77.3	65.5	98.9	50.7	45.5	63.9
	Deaths	-18.0	-22.8	-11.8	35.1	34.2	34.1	7.5	0.3	16.4
	DALYs	-15.9	-19.4	-12.3	42.9	37.5	47.3	8.9	3.1	15.8
	YLLs	-17.7	-21.8	-13.4	40.5	34.9	45.2	5.1	-1.7	12.9
	YLDs	16.7	15.4	15.5	72.7	62.7	88.1	47.0	42.2	57.6
Lorestan	Incidence	16.5	12.9	22.2	35.3	26.0	52.1	41.2	37.4	50.1
	Prevalence	27.7	22.5	36.5	38.8	28.7	59.2	47.6	42.6	61.9
	Deaths	-18.5	-25.0	-5.1	18.7	8.9	31.2	1.0	-6.7	9.2
	DALYs	-15.9	-21.6	-6.6	18.0	7.8	30.7	6.3	0.9	12.6
	YLLs	-18.4	-24.7	-8.2	16.2	5.5	29.1	1.9	-4.8	9.3
	YLDs	21.9	17.4	29.6	37.0	26.7	56.4	44.3	39.7	55.4

Location	Measure	% Change (1990 to 2000)			% Change (2000 to 2010)			% Change (2010 to 2019)		
		Both	Female	Male	Both	Female	Male	Both	Female	Male
Markazi	Incidence	16.9	14.0	25.5	93.9	91.9	96.8	39.7	38.0	44.6
	Prevalence	29.3	24.7	42.5	103.4	100.1	110.3	42.8	40.6	50.2
	Deaths	-19.7	-24.3	-8.5	51.1	47.3	55.4	16.0	13.2	18.7
	DALYs	-15.1	-19.8	-6.5	62.2	58.4	65.6	13.9	12.4	15.2
	YLLs	-17.6	-22.8	-8.3	58.9	54.3	63.3	10.5	8.4	12.6
	YLDs	22.2	18.3	33.0	96.0	93.6	99.9	41.8	39.7	47.5
Mazandaran	Incidence	23.7	20.4	33.3	24.9	18.0	41.7	42.6	40.7	47.1
	Prevalence	31.7	26.7	45.0	26.7	19.3	46.0	45.5	43.1	51.7
	Deaths	-14.2	-19.1	-1.7	12.2	6.3	20.6	18.4	14.8	22.5
	DALYs	-8.2	-12.8	1.1	14.5	7.4	24.4	19.7	17.6	21.9
	YLLs	-11.5	-16.9	-1.3	12.9	5.4	22.6	16.0	13.1	19.1
	YLDs	26.6	22.2	38.5	26.2	18.7	44.0	43.3	40.9	48.5
North Khorasan	Incidence	28.9	26.1	33.5	32.4	24.8	47.7	70.4	75.9	58.5
	Prevalence	50.0	43.4	63.5	37.6	28.7	59.0	76.7	81.8	66.4
	Deaths	-16.7	-19.8	-6.3	10.7	4.2	18.8	33.5	33.7	30.7
	DALYs	-12.5	-16.6	-4.5	11.7	3.9	21.1	34.8	38.9	28.1
	YLLs	-15.0	-19.5	-6.2	9.8	1.7	19.5	30.9	33.9	25.8
	YLDs	37.6	33.0	45.8	35.2	26.7	52.8	74.0	79.7	62.0
Qazvin	Incidence	32.0	31.7	36.4	61.3	56.3	70.1	37.5	34.5	51.4
	Prevalence	50.1	47.6	58.6	65.8	60.3	77.8	43.2	39.7	62.7
	Deaths	-15.7	-18.8	0.7	42.2	35.8	50.2	1.2	-6.8	15.2
	DALYs	-10.4	-13.5	-0.3	41.5	35.5	48.5	5.8	0.0	17.6
	YLLs	-13.5	-17.2	-2.1	39.4	33.0	47.1	1.8	-5.2	14.8
	YLDs	39.4	38.0	45.4	62.5	57.4	71.1	40.4	36.9	56.6
Qom	Incidence	23.0	22.1	31.4	26.8	26.2	23.0	38.7	34.1	55.5
	Prevalence	40.0	36.5	54.3	31.5	29.4	29.8	43.5	38.2	65.4
	Deaths	-18.5	-20.5	-5.0	7.2	9.8	4.2	10.3	6.4	19.6
	DALYs	-13.7	-16.7	-3.3	7.1	8.5	3.1	11.3	6.9	20.5
	YLLs	-16.3	-19.7	-5.1	5.2	6.5	1.8	7.9	3.0	17.5
	YLDs	30.5	28.2	42.3	27.9	27.1	23.5	42.4	37.0	62.4

Location	Measure	% Change (1990 to 2000)			% Change (2000 to 2010)			% Change (2010 to 2019)		
		Both	Female	Male	Both	Female	Male	Both	Female	Male
Semnan	Incidence	24.9	22.0	34.6	71.1	58.4	95.5	40.1	34.9	50.4
	Prevalence	39.5	34.7	52.8	77.3	63.7	106.2	45.6	39.4	59.1
	Deaths	-15.3	-22.2	0.4	44.8	28.7	66.5	8.6	-2.5	20.1
	DALYs	-11.3	-17.6	0.1	49.2	33.6	68.4	12.1	3.5	20.9
	YLLs	-14.0	-21.1	-1.8	46.8	30.3	66.4	8.6	-1.5	18.2
	YLDs	30.6	26.8	41.7	73.2	60.1	98.2	42.2	35.9	54.5
Sistan and Baluchistan	Incidence	21.0	23.9	13.5	103.8	94.3	108.8	87.5	80.9	101.3
	Prevalence	48.5	46.9	44.8	119.9	105.7	134.9	95.1	87.3	115.3
	Deaths	-19.7	-21.6	-16.1	59.8	52.3	65.2	49.7	37.9	62.4
	DALYs	-14.7	-15.5	-13.7	73.3	64.8	79.6	50.4	41.2	60.8
	YLLs	-16.4	-17.8	-14.8	71.0	62.1	78.0	47.2	36.8	58.3
	YLDs	31.7	33.1	25.2	110.4	99.3	117.9	93.6	86.8	108.7
South Khorasan	Incidence	9.6	7.1	11.2	40.9	35.5	50.3	56.9	53.1	65.9
	Prevalence	22.2	17.3	28.6	45.9	39.8	58.6	62.2	57.2	76.1
	Deaths	-23.4	-27.0	-16.7	18.4	10.6	27.7	25.2	20.8	30.4
	DALYs	-21.4	-24.7	-16.7	21.2	14.4	29.3	25.8	22.4	29.6
	YLLs	-23.5	-27.2	-18.2	19.3	11.9	27.9	22.2	18.0	26.7
	YLDs	14.6	10.8	18.8	43.1	37.8	52.3	60.1	55.4	71.6
Tehran	Incidence	8.3	6.2	20.3	-19.2	-23.7	-10.0	23.6	18.9	34.2
	Prevalence	14.7	11.4	29.8	-20.0	-24.4	-11.0	25.4	19.9	39.2
	Deaths	-26.3	-29.9	-12.0	-14.4	-17.9	-7.9	7.4	5.9	9.5
	DALYs	-19.8	-23.3	-8.6	-16.8	-21.2	-10.3	7.4	4.0	12.0
	YLLs	-23.2	-27.3	-11.0	-16.4	-20.7	-10.3	4.7	1.1	9.5
	YLDs	11.7	8.9	25.3	-19.2	-23.7	-10.2	25.3	20.1	37.1
West Azarbayejan	Incidence	17.6	15.1	22.8	61.9	61.2	56.5	58.8	57.9	61.9
	Prevalence	31.8	27.4	40.7	73.3	70.7	71.9	65.0	62.6	73.0
	Deaths	-14.0	-19.5	-2.4	22.6	18.7	25.1	25.3	24.8	25.1
	DALYs	-10.7	-15.9	-2.3	27.9	26.0	27.6	25.5	24.8	25.8
	YLLs	-12.6	-18.3	-3.6	24.9	22.2	25.9	21.7	20.2	23.0
	YLDs	23.9	20.6	30.2	65.3	64.2	60.4	62.1	60.0	67.9

Location	Measure	% Change (1990 to 2000)			% Change (2000 to 2010)			% Change (2010 to 2019)		
		Both	Female	Male	Both	Female	Male	Both	Female	Male
Yazd	Incidence	31.8	33.3	36.4	89.4	91.7	81.4	36.7	31.6	53.5
	Prevalence	49.3	47.9	58.4	98.3	97.7	96.0	41.0	35.1	62.4
	Deaths	-14.9	-18.1	-1.0	46.9	51.7	43.4	8.0	2.8	16.2
	DALYs	-9.8	-12.6	-0.4	52.2	57.5	44.2	9.4	3.6	20.1
	YLLs	-13.0	-16.6	-2.4	48.0	52.8	41.5	5.6	-1.1	16.8
	YLDs	39.2	39.6	44.6	92.5	94.1	85.5	38.2	32.2	57.8
Zanjan	Incidence	12.1	9.4	17.8	85.4	75.6	102.0	47.3	46.0	50.8
	Prevalence	24.6	20.4	30.3	96.5	85.0	119.3	54.0	51.4	61.8
	Deaths	-17.3	-22.8	-3.0	48.7	35.1	65.3	13.2	8.6	17.9
	DALYs	-13.2	-18.4	-3.8	52.6	40.0	67.4	13.4	10.6	16.4
	YLLs	-14.9	-20.6	-5.0	49.7	36.1	65.3	9.7	5.7	13.7
	YLDs	17.0	13.0	24.4	88.9	79.2	104.4	50.5	48.4	55.9
