# Supplementary material for: National, sub-national, and risk-attributed burden of thyroid cancer in Iran from 1990 to 2019
Source: Sci Rep. 2022 Aug 2;12:13231. doi: 10.1038/s41598-022-17115-0 (PMC9346133; doi:10.1038/s41598-022-17115-0)
Supplement: Supplementary file 7 — Supplementary Table 3. [file 41598_2022_17115_MOESM7_ESM.pdf]

Location		Sex	New cases		Expected new cases in 2019		% 1990 - 2019 new cases change cause			% 1990 - 2019 new cases overall change
			1990	2019	Population growth	Population growth + Aging	Population growth	Age structure change	Incidence rate change	
**Iran (Islamic Republic of)**		Both	539	3198	776	1353	44.0%	106.9%	342.3%	493.1%
		Female	400	2242	580	1035	44.9%	113.6%	301.6%	460.1%
		Male	139	956	199	334	43.1%	97.6%	447.7%	588.5%
**Sub-national**	Alborz	Both	21	157	40	78	95.4%	180.8%	383.1%	659.2%
		Female	16	105	32	63	99.0%	197.2%	259.8%	556.0%
		Male	5	52	9	17	92.0%	164.2%	756.8%	1013%
	Ardebil	Both	9	49	10	18	10.9%	96.0%	352.3%	459.2%
		Female	7	35	7	14	10.9%	102.6%	306.1%	419.6%
		Male	2	14	2	4	10.8%	85.4%	495.2%	591.5%
	Bushehr	Both	5	51	8	14	71.4%	128.3%	773.0%	972.7%
		Female	4	40	6	11	64.7%	130.4%	818.2%	1013.3%
		Male	1	10	2	3	78.0%	120.7%	643.0%	841.6%
	Chahar Mahaal and Bakhtiari	Both	5	30	7	13	35.3%	116.4%	348.1%	499.8%
		Female	4	20	5	9	36.0%	126.4%	294.0%	456.3%
		Male	2	11	2	4	34.8%	105.5%	460.9%	601.2%
	East Azarbayejan	Both	24	199	29	51	18.2%	90.4%	606.7%	715.3%
		Female	17	137	20	37	18.1%	97.2%	573%	688.4%
		Male	7	63	8	14	18.2%	82.3%	680.4%	780.9%
	Fars	Both	24	190	33	61	35.9%	114.8%	535.0%	685.8%
		Female	17	132	24	44	36.5%	120.3%	504.4%	661.3%
		Male	7	59	9	17	35.4%	107.0%	604.6%	747.0%
	Gilan	Both	21	118	23	44	11.6%	100.7%	362.1%	474.4%
		Female	15	84	17	32	11.7%	101.5%	337.0%	450.2%
		Male	5	34	6	11	11.4%	98.2%	434.5%	544.2%
	Golestan	Both	7	66	10	17	43.9%	111.6%	712.1%	867.7%
		Female	5	44	7	13	44.3%	118.6%	653.4%	816.4%
		Male	2	22	3	5	43.5%	104.1%	842.0%	989.6%
	Hamadan	Both	14	82	14	26	3.4%	83.8%	400.7%	488.0%
		Female	10	51	10	19	5.1%	88.6%	335.8%	429.6%
		Male	4	31	4	8	1.8%	76.8%	538.2%	616.9%

Location		Sex	New cases		Expected new cases in 2019		% 1990 - 2019 new cases change cause			% 1990 - 2019 new cases overall change
			1990	2019	Population growth	Population growth + Aging	Population growth	Age structure change	Incidence rate change	
	Hormozgan	Both	4	39	8	12	105.0%	107.7%	702.1%	914.7%
		Female	2	21	5	8	105.1%	122.4%	552.6%	780.1%
		Male	1	17	3	4	104.9%	90.4%	961.2%	1156.5%
	Ilam	Both	2	20	3	5	32.0%	134.7%	701.3%	868.0%
		Female	1	13	2	4	33.7%	158.4%	623.8%	815.9%
		Male	1	7	1	2	30.4%	112.7%	839.2%	982.3%
	Isfahan	Both	33	238	45	82	37.1%	113.8%	472.9%	623.7%
		Female	24	172	34	62	39.7%	116.3%	458.7%	614.7%
		Male	9	65	12	21	34.6%	107.5%	506.5%	648.6%
	Kerman	Both	12	121	21	35	78.3%	114.1%	717.7%	910.1%
		Female	8	86	15	25	76.0%	120.4%	726.1%	922.6%
		Male	4	35	6	10	80.5%	102.9%	696.8%	880.2%
	Kermanshah	Both	11	87	12	24	16.3%	106.2%	597.7%	720.3%
		Female	7	59	9	17	18.7%	118.9%	577.2%	714.9%
		Male	3	28	4	7	14.1%	94.6%	622.9%	731.6%
	Khorasan-e-Razavi	Both	28	203	39	64	41.4%	90.4%	499.6%	631.4%
		Female	20	145	28	47	41.7%	98.1%	501.9%	641.7%
		Male	8	58	12	18	41.1%	80.7%	485.1%	606.9%
	Khuzestan	Both	23	185	35	62	52.2%	116.8%	538.2%	707.2%
		Female	18	145	28	50	53.2%	122.4%	526.2%	701.8%
		Male	5	40	7	12	51.3%	105.3%	571.0%	727.5%
	Kohgiluyeh and Boyer-Ahmad	Both	3	28	5	9	51.9%	111.5%	549.8%	713.2%
		Female	2	17	3	6	51.9%	122.8%	485.9%	660.6%
		Male	1	11	2	3	52.0%	96.7%	664.8%	813.6%
	Kurdistan	Both	7	47	9	17	33.8%	106.1%	432.3%	572.2%
		Female	5	31	6	12	34.2%	116.9%	393.7%	544.8%
		Male	2	16	3	5	33.5%	95.3%	502.4%	631.2%
	Lorestan	Both	17	86	20	38	14.3%	102.9%	277.0%	394.2%
		Female	13	60	15	30	15.5%	114.8%	233.8%	364.1%
		Male	5	26	5	9	13.2%	89.3%	375.9%	478.3%

Location	Sex	New cases		Expected new cases in 2019		% 1990 - 2019 new cases change cause			% 1990 - 2019 new cases overall change
		1990	2019	Population growth	Population growth + Aging	Population growth	Age structure change	Incidence rate change	
Markazi	Both	10	70	12	22	19.2%	96.6%	479.7%	595.5%
	Female	7	49	9	16	18.5%	98.1%	449.4%	566.1%
	Male	3	21	3	6	19.9%	91.4%	562.0%	673.3%
Mazandaran	Both	26	147	35	66	31.5%	117.5%	305.7%	454.7%
	Female	20	100	26	50	31.3%	120.9%	256.1%	408.3%
	Male	7	47	9	16	31.6%	111.6%	446.8%	590.0%
North Khorasan	Both	4	29	6	10	37.5%	89.1%	449.8%	576.4%
	Female	3	21	4	7	37.6%	97.0%	432.2%	566.8%
	Male	1	8	2	2	37.3%	77.5%	488.0%	602.8%
Qazvin	Both	6	46	8	15	39.9%	116.5%	528.0%	684.4%
	Female	5	36	7	12	40.4%	120.3%	497.1%	657.9%
	Male	1	11	2	3	39.3%	105.4%	645.0%	789.7%
Qom	Both	7	51	13	23	84.1%	135.5%	386.4%	605.9%
	Female	6	38	10	18	85.8%	138.3%	349.1%	573.2%
	Male	2	13	3	5	82.4%	128.0%	507.4%	717.8%
Semnan	Both	3	26	5	8	54.9%	89.2%	517.2%	661.3%
	Female	2	16	4	6	57.1%	88.1%	424.2%	569.3%
	Male	1	10	2	2	52.9%	88.3%	737.4%	878.7%
Sistan and Baluchistan	Both	6	84	12	15	101.9%	57.8%	1156.7%	1316.4%
	Female	4	55	8	11	102.0%	81.3%	1173.1%	1356.4%
	Male	2	29	4	5	101.9%	32.5%	1112.3%	1246.7%
South Khorasan	Both	7	31	8	13	24.2%	62.4%	272.6%	359.2%
	Female	5	21	6	9	24.1%	72.5%	246.2%	342.8%
	Male	2	10	2	3	24.4%	49.2%	325.9%	399.5%
Tehran	Both	178	539	292	496	63.8%	114.9%	24.0%	202.8%
	Female	138	383	230	399	67.3%	122.8%	-12.1%	178.0%
	Male	40	156	65	106	60.5%	102.6%	123.9%	287.0%
West Azarbayejan	Both	13	106	20	33	46.6%	100.6%	538.7%	686.0%
	Female	10	76	14	25	46.8%	109.4%	530.6%	686.8%
	Male	4	30	6	9	46.5%	90.0%	547.3%	683.8%

Location		Sex	New cases		Expected new cases in 2019		% 1990 - 2019 new cases change cause			% 1990 - 2019 new cases overall change
			1990	2019	Population growth	Population growth + Aging	Population growth	Age structure change	Incidence rate change	
	Yazd	Both	5	48	8	13	69.2%	99.9%	701.4%	870.4%
		Female	4	35	6	10	70.3%	94.5%	669.7%	834.5%
		Male	1	13	2	3	68.1%	100.6%	810.8%	979.5%
	Zanjan	Both	4	26	5	8	21.6%	99.0%	470.6%	591.1%
		Female	3	17	3	6	22.1%	106.8%	433.4%	562.3%
		Male	1	8	1	2	21.0%	87.2%	550.9%	659.1%
