# Supplementary material for: National, sub-national, and risk-attributed burden of thyroid cancer in Iran from 1990 to 2019
Source: Sci Rep. 2022 Aug 2;12:13231. doi: 10.1038/s41598-022-17115-0 (PMC9346133; doi:10.1038/s41598-022-17115-0)
Supplement: Supplementary file 8 — Supplementary Table 4. [file 41598_2022_17115_MOESM8_ESM.pdf]

Province	Measure	Attributed age-standardized rate (per 100,000)						% Change (1990 to 2019)		
		1990			2019					
		Both	Female	Male	Both	Female	Male	Both	Female	Male
Alborz	Deaths	0.06 (0.03 to 0.1)	0.08 (0.03 to 0.14)	0.04 (0.01 to 0.08)	0.08 (0.04 to 0.14)	0.09 (0.05 to 0.14)	0.08 (0.02 to 0.16)	51.3 (2.6 to 149.3)	13.3 (-23.7 to 97.9)	135.7 (38.3 to 348.1)
	DALYs	1.46 (0.62 to 2.61)	2.03 (0.87 to 3.58)	0.94 (0.25 to 2.21)	2.33 (1.1 to 3.92)	2.32 (1.09 to 3.91)	2.35 (0.66 to 4.48)	59.5 (7.4 to 179.6)	14.1 (-23.6 to 108.2)	150.2 (47.3 to 373.7)
	YLLs	1.34 (0.57 to 2.37)	1.83 (0.79 to 3.29)	0.89 (0.24 to 2.11)	1.96 (0.92 to 3.33)	1.87 (0.9 to 3.18)	2.08 (0.58 to 3.93)	46.6 (-2.3 to 155.7)	1.9 (-32.1 to 86.5)	133.2 (35.9 to 348.1)
	YLDs	0.12 (0.04 to 0.23)	0.2 (0.07 to 0.4)	0.05 (0.01 to 0.12)	0.36 (0.15 to 0.67)	0.45 (0.18 to 0.88)	0.28 (0.08 to 0.59)	204.4 (89.3 to 497.9)	127.5 (29.2 to 376.8)	451.8 (186.4 to 1169.6)
Ardebil	Deaths	0.03 (0.02 to 0.06)	0.05 (0.02 to 0.09)	0.02 (0.01 to 0.05)	0.07 (0.04 to 0.12)	0.07 (0.04 to 0.12)	0.07 (0.02 to 0.14)	117.5 (43 to 244.4)	51.3 (-7.2 to 151.4)	248.8 (115.4 to 558.7)
	DALYs	0.88 (0.4 to 1.52)	1.27 (0.59 to 2.27)	0.55 (0.15 to 1.31)	1.98 (0.99 to 3.29)	2.03 (1.05 to 3.37)	1.94 (0.59 to 3.67)	124.2 (43.7 to 257)	60.2 (-3.9 to 165.6)	249.7 (110.6 to 555.7)
	YLLs	0.83 (0.38 to 1.44)	1.18 (0.54 to 2.13)	0.53 (0.15 to 1.25)	1.73 (0.86 to 2.93)	1.7 (0.89 to 2.85)	1.77 (0.54 to 3.37)	108.6 (32.6 to 234)	44.1 (-12.5 to 145.6)	232.4 (96 to 522.6)
	YLDs	0.05 (0.02 to 0.1)	0.09 (0.03 to 0.17)	0.02 (0.01 to 0.05)	0.25 (0.11 to 0.45)	0.33 (0.14 to 0.62)	0.17 (0.04 to 0.36)	367.3 (182.2 to 677.5)	276.5 (108.3 to 572.1)	662.7 (310.1 to 1427.5)
Bushehr	Deaths	0.03 (0.02 to 0.06)	0.05 (0.02 to 0.09)	0.02 (0.01 to 0.05)	0.08 (0.04 to 0.13)	0.1 (0.05 to 0.16)	0.07 (0.02 to 0.13)	144.9 (34.3 to 304.9)	112.7 (-5.4 to 273.4)	212.7 (77 to 508.7)
	DALYs	0.85 (0.39 to 1.56)	1.17 (0.54 to 2.17)	0.55 (0.15 to 1.31)	2.24 (1.1 to 3.59)	2.78 (1.2 to 4.71)	1.73 (0.49 to 3.26)	162.8 (34.4 to 334.4)	137.9 (-0.5 to 303)	212.2 (77.4 to 510.4)
	YLLs	0.8 (0.36 to 1.45)	1.08 (0.5 to 2)	0.53 (0.14 to 1.27)	1.92 (0.94 to 3.09)	2.29 (0.99 to 3.85)	1.56 (0.46 to 2.94)	141.5 (25.2 to 303.1)	112.4 (-10.1 to 258.9)	196.5 (68.4 to 483.3)
	YLDs	0.06 (0.02 to 0.11)	0.09 (0.04 to 0.18)	0.03 (0.01 to 0.06)	0.32 (0.14 to 0.59)	0.49 (0.18 to 0.97)	0.16 (0.04 to 0.33)	460.6 (154.4 to 897.2)	441.3 (100.3 to 931.6)	537.8 (234.3 to 1237.7)

Province	Measure	Attributed age-standardized rate (per 100,000)						% Change (1990 to 2019)		
		1990			2019					
		Both	Female	Male	Both	Female	Male	Both	Female	Male
Chahar Mahaal and Bakhtiari	Deaths	0.02 (0.01 to 0.05)	0.03 (0.01 to 0.06)	0.02 (0 to 0.04)	0.04 (0.02 to 0.08)	0.04 (0.02 to 0.06)	0.05 (0.01 to 0.1)	74.7 (8.3 to 189.8)	14.9 (-32.6 to 93.7)	173.2 (54.5 to 431)
	DALYs	0.63 (0.26 to 1.19)	0.8 (0.35 to 1.53)	0.48 (0.12 to 1.13)	1.21 (0.53 to 2.16)	1.05 (0.53 to 1.89)	1.37 (0.37 to 2.86)	92.6 (22.3 to 211.3)	32.6 (-20.3 to 120.9)	184.2 (58.9 to 452)
	YLLs	0.58 (0.24 to 1.11)	0.73 (0.31 to 1.41)	0.46 (0.11 to 1.06)	1.03 (0.46 to 1.87)	0.84 (0.42 to 1.5)	1.22 (0.33 to 2.54)	76.4 (8.5 to 189.5)	15.7 (-31.9 to 91.8)	166.8 (48.4 to 418.5)
	YLDs	0.04 (0.02 to 0.09)	0.07 (0.03 to 0.14)	0.02 (0.01 to 0.06)	0.18 (0.08 to 0.35)	0.21 (0.09 to 0.42)	0.15 (0.04 to 0.33)	303.1 (125.1 to 597.6)	213.8 (57.1 to 482.9)	510.1 (200.9 to 1190)
East Azarbayejan	Deaths	0.03 (0.01 to 0.06)	0.04 (0.02 to 0.08)	0.03 (0.01 to 0.06)	0.09 (0.04 to 0.15)	0.09 (0.05 to 0.15)	0.09 (0.02 to 0.18)	165.1 (25.3 to 332.8)	112.1 (-10 to 270.5)	244.3 (72.3 to 530)
	DALYs	0.84 (0.38 to 1.58)	1.04 (0.48 to 1.94)	0.66 (0.18 to 1.6)	2.38 (1.07 to 3.98)	2.35 (1.14 to 4.01)	2.4 (0.63 to 4.79)	182.9 (26.5 to 361)	126.5 (-7.3 to 288.2)	261.7 (74.2 to 571)
	YLLs	0.79 (0.35 to 1.49)	0.97 (0.44 to 1.8)	0.64 (0.17 to 1.52)	2.08 (0.94 to 3.52)	1.97 (0.97 to 3.36)	2.18 (0.56 to 4.34)	162.4 (17 to 325.3)	104.4 (-15.5 to 255.6)	241.9 (66.3 to 542.6)
	YLDs	0.05 (0.02 to 0.09)	0.07 (0.03 to 0.15)	0.03 (0.01 to 0.06)	0.3 (0.12 to 0.57)	0.38 (0.15 to 0.75)	0.22 (0.06 to 0.49)	521.6 (144.6 to 977.6)	425.6 (99.3 to 938.1)	732.1 (256.9 to 1682.5)
Fars	Deaths	0.03 (0.01 to 0.06)	0.04 (0.02 to 0.07)	0.02 (0.01 to 0.05)	0.06 (0.03 to 0.1)	0.06 (0.03 to 0.11)	0.06 (0.02 to 0.12)	110.5 (28.2 to 252.7)	76.9 (-5.1 to 216.2)	166 (54.1 to 400.4)
	DALYs	0.74 (0.32 to 1.41)	0.9 (0.41 to 1.74)	0.59 (0.15 to 1.4)	1.73 (0.85 to 2.87)	1.75 (0.92 to 3)	1.72 (0.53 to 3.35)	133.7 (37.7 to 281.6)	93.9 (2.7 to 234.7)	192.5 (63.4 to 472.1)
	YLLs	0.69 (0.3 to 1.32)	0.83 (0.36 to 1.6)	0.56 (0.14 to 1.34)	1.48 (0.73 to 2.47)	1.43 (0.76 to 2.43)	1.53 (0.47 to 3.02)	114.3 (27.1 to 252)	72.7 (-10.6 to 201.6)	174.1 (53.1 to 442.3)
	YLDs	0.05 (0.02 to 0.1)	0.08 (0.03 to 0.16)	0.03 (0.01 to 0.07)	0.25 (0.12 to 0.46)	0.32 (0.15 to 0.61)	0.19 (0.06 to 0.4)	393.3 (153.5 to 804.4)	324.1 (99.8 to 760.2)	552.7 (193 to 1357)

Province	Measure	Attributed age-standardized rate (per 100,000)						% Change (1990 to 2019)		
		1990			2019					
		Both	Female	Male	Both	Female	Male	Both	Female	Male
**Gilan**	**Deaths**	0.03 (0.01 to 0.06)	0.04 (0.02 to 0.08)	0.02 (0.01 to 0.06)	0.07 (0.03 to 0.11)	0.07 (0.04 to 0.11)	0.06 (0.02 to 0.12)	99.8 (20.4 to 216.3)	75.6 (-4.4 to 196.8)	151.8 (44.2 to 357.7)
	**DALYs**	0.84 (0.38 to 1.59)	1.04 (0.48 to 1.94)	0.62 (0.16 to 1.37)	1.8 (0.92 to 2.97)	1.92 (1 to 3.11)	1.68 (0.48 to 3.19)	114.3 (24.5 to 241.9)	83.6 (-2.5 to 206.8)	169.5 (53.3 to 388.1)
	**YLLs**	0.77 (0.35 to 1.46)	0.95 (0.43 to 1.76)	0.59 (0.15 to 1.3)	1.54 (0.8 to 2.53)	1.58 (0.84 to 2.56)	1.5 (0.45 to 2.89)	99.5 (15.3 to 219.2)	67.3 (-10.1 to 182.5)	155 (42.7 to 368.2)
	**YLDs**	0.07 (0.03 to 0.13)	0.1 (0.04 to 0.2)	0.03 (0.01 to 0.08)	0.26 (0.12 to 0.46)	0.34 (0.15 to 0.63)	0.18 (0.05 to 0.38)	286.8 (103.9 to 577.9)	238.7 (63.2 to 559.4)	420.6 (159.8 to 985.9)
**Golestan**	**Deaths**	0.03 (0.01 to 0.06)	0.04 (0.02 to 0.08)	0.03 (0.01 to 0.06)	0.08 (0.04 to 0.13)	0.07 (0.04 to 0.11)	0.09 (0.03 to 0.17)	150.7 (32.4 to 292)	88.3 (-9.5 to 220.1)	241.3 (82.2 to 510.5)
	**DALYs**	0.81 (0.37 to 1.58)	0.96 (0.42 to 1.96)	0.67 (0.18 to 1.53)	2.21 (1.07 to 3.69)	2 (1.1 to 3.37)	2.43 (0.7 to 4.75)	173 (38.3 to 324.2)	109.4 (-0.5 to 248.3)	261.6 (90.8 to 554)
	**YLLs**	0.77 (0.34 to 1.51)	0.9 (0.4 to 1.85)	0.65 (0.17 to 1.47)	1.96 (0.93 to 3.29)	1.71 (0.93 to 2.88)	2.24 (0.65 to 4.42)	155.8 (29.6 to 303)	90.5 (-8 to 220.4)	245 (81.4 to 519.2)
	**YLDs**	0.04 (0.02 to 0.09)	0.06 (0.02 to 0.14)	0.02 (0.01 to 0.06)	0.25 (0.11 to 0.45)	0.3 (0.14 to 0.57)	0.2 (0.05 to 0.39)	488.2 (169.8 to 939.5)	388.8 (121.2 to 873.1)	699.3 (286.8 to 1486.6)
**Hamadan**	**Deaths**	0.03 (0.01 to 0.06)	0.04 (0.02 to 0.08)	0.03 (0.01 to 0.06)	0.08 (0.04 to 0.14)	0.07 (0.04 to 0.12)	0.09 (0.03 to 0.18)	150.1 (42.1 to 307.3)	79.4 (-3.7 to 202.8)	261.9 (81.4 to 592.1)
	**DALYs**	0.87 (0.4 to 1.58)	1.06 (0.48 to 1.92)	0.7 (0.19 to 1.61)	2.36 (1.03 to 3.99)	2.02 (1.03 to 3.43)	2.69 (0.75 to 5.27)	171 (44.4 to 336)	90.9 (1.2 to 222.2)	285.6 (93.5 to 634.2)
	**YLLs**	0.82 (0.37 to 1.49)	0.98 (0.44 to 1.79)	0.67 (0.18 to 1.54)	2.07 (0.9 to 3.52)	1.7 (0.88 to 2.86)	2.43 (0.68 to 4.83)	153.4 (36.4 to 309.3)	73.5 (-7.2 to 196.6)	264.8 (81.9 to 614.3)
	**YLDs**	0.05 (0.02 to 0.11)	0.08 (0.03 to 0.16)	0.03 (0.01 to 0.08)	0.29 (0.12 to 0.54)	0.32 (0.13 to 0.61)	0.26 (0.06 to 0.56)	434.4 (156.7 to 808.7)	305.4 (88.7 to 645.2)	728.8 (267.8 to 1667.2)

Province	Measure	Attributed age-standardized rate (per 100,000)						% Change (1990 to 2019)		
		1990			2019					
		Both	Female	Male	Both	Female	Male	Both	Female	Male
Hormozgan	Deaths	0.03 (0.01 to 0.05)	0.03 (0.01 to 0.07)	0.02 (0.01 to 0.06)	0.05 (0.03 to 0.09)	0.04 (0.02 to 0.08)	0.06 (0.02 to 0.13)	96.7 (16.9 to 235.6)	37.6 (-11.4 to 128.2)	177.4 (53.9 to 454.7)
	DALYs	0.69 (0.29 to 1.38)	0.78 (0.34 to 1.64)	0.6 (0.15 to 1.47)	1.49 (0.69 to 2.6)	1.14 (0.6 to 2)	1.84 (0.53 to 3.64)	116.3 (22.8 to 276.9)	46.3 (-11.7 to 156.4)	204 (65.9 to 539.1)
	YLLs	0.66 (0.27 to 1.34)	0.74 (0.32 to 1.57)	0.58 (0.14 to 1.42)	1.32 (0.61 to 2.31)	0.98 (0.52 to 1.71)	1.67 (0.49 to 3.35)	101 (15.9 to 251.8)	31.9 (-19 to 126.9)	186.4 (56.8 to 507.2)
	YLDs	0.03 (0.01 to 0.06)	0.04 (0.02 to 0.09)	0.02 (0 to 0.05)	0.16 (0.07 to 0.31)	0.17 (0.08 to 0.32)	0.16 (0.04 to 0.35)	456 (178.9 to 948.1)	303.8 (102.3 to 754.7)	734.9 (321.1 to 1837.9)
Ilam	Deaths	0.02 (0.01 to 0.05)	0.03 (0.01 to 0.07)	0.02 (0 to 0.04)	0.06 (0.03 to 0.11)	0.06 (0.03 to 0.1)	0.07 (0.02 to 0.13)	190.8 (67.6 to 404.3)	93.1 (5.9 to 253.5)	335.6 (131.2 to 804.6)
	DALYs	0.55 (0.23 to 1.14)	0.75 (0.31 to 1.52)	0.41 (0.1 to 1)	1.76 (0.87 to 2.91)	1.63 (0.88 to 2.75)	1.89 (0.53 to 3.57)	217.9 (80.8 to 463.8)	117.6 (17.8 to 296.6)	359.4 (137.7 to 870.7)
	YLLs	0.52 (0.21 to 1.07)	0.69 (0.28 to 1.42)	0.39 (0.09 to 0.97)	1.53 (0.75 to 2.56)	1.35 (0.73 to 2.27)	1.7 (0.49 to 3.25)	194 (66.7 to 423.7)	94.6 (6.3 to 257.6)	333.1 (125 to 813.4)
	YLDs	0.03 (0.01 to 0.07)	0.05 (0.02 to 0.12)	0.02 (0 to 0.05)	0.23 (0.11 to 0.41)	0.28 (0.13 to 0.52)	0.19 (0.05 to 0.38)	581.6 (259.1 to 1205.1)	411.5 (150.1 to 960.1)	924.2 (388.7 to 2184.2)
Isfahan	Deaths	0.03 (0.01 to 0.06)	0.04 (0.02 to 0.08)	0.02 (0.01 to 0.05)	0.07 (0.04 to 0.12)	0.08 (0.04 to 0.13)	0.06 (0.02 to 0.13)	116.8 (20.6 to 254.8)	95.4 (-3 to 260.8)	163.2 (58.3 to 367.5)
	DALYs	0.82 (0.37 to 1.49)	1.03 (0.44 to 1.91)	0.62 (0.16 to 1.38)	1.92 (0.96 to 3.23)	2.11 (1.04 to 3.59)	1.74 (0.52 to 3.45)	134.2 (30.9 to 276.9)	105.8 (0.7 to 269.7)	182.3 (65.6 to 421.9)
	YLLs	0.76 (0.33 to 1.38)	0.93 (0.4 to 1.76)	0.58 (0.15 to 1.28)	1.64 (0.81 to 2.74)	1.74 (0.85 to 2.93)	1.55 (0.47 to 3.08)	117.2 (19.4 to 249.6)	86.8 (-8.7 to 244.9)	166.9 (56.6 to 394.5)
	YLDs	0.06 (0.03 to 0.13)	0.1 (0.04 to 0.21)	0.03 (0.01 to 0.08)	0.28 (0.12 to 0.52)	0.38 (0.14 to 0.75)	0.19 (0.05 to 0.39)	334.1 (123.4 to 657.3)	288 (69.6 to 683.8)	446.6 (171.5 to 1004.5)

Province	Measure	Attributed age-standardized rate (per 100,000)						% Change (1990 to 2019)		
		1990			2019					
		Both	Female	Male	Both	Female	Male	Both	Female	Male
Kerman	Deaths	0.04 (0.02 to 0.07)	0.05 (0.02 to 0.08)	0.03 (0.01 to 0.07)	0.08 (0.04 to 0.13)	0.08 (0.04 to 0.14)	0.07 (0.02 to 0.14)	108.2 (4.2 to 214.1)	84.4 (-16.7 to 198.3)	145.1 (25 to 311.1)
	DALYs	0.96 (0.47 to 1.74)	1.16 (0.56 to 2.05)	0.78 (0.22 to 1.73)	2.14 (1.02 to 3.54)	2.26 (1.06 to 3.8)	2.02 (0.55 to 3.88)	122.1 (1.7 to 227.2)	94.6 (-14.5 to 202.9)	160.1 (26.1 to 347.2)
	YLLs	0.91 (0.44 to 1.66)	1.08 (0.51 to 1.93)	0.75 (0.21 to 1.67)	1.89 (0.89 to 3.12)	1.93 (0.91 to 3.29)	1.85 (0.5 to 3.56)	107.4 (-4 to 210.4)	77.7 (-19.8 to 182.7)	147.6 (21 to 325.2)
	YLDs	0.05 (0.02 to 0.1)	0.08 (0.03 to 0.15)	0.03 (0.01 to 0.07)	0.25 (0.11 to 0.47)	0.33 (0.13 to 0.64)	0.17 (0.05 to 0.37)	375 (109.7 to 682.7)	331.3 (65 to 676.6)	466.2 (160.1 to 989.4)
Kermanshah	Deaths	0.03 (0.01 to 0.06)	0.04 (0.02 to 0.08)	0.03 (0.01 to 0.06)	0.08 (0.04 to 0.13)	0.08 (0.04 to 0.13)	0.08 (0.02 to 0.16)	139.9 (20.7 to 304.9)	92.8 (-9.9 to 244.1)	203 (49.1 to 487.4)
	DALYs	0.85 (0.36 to 1.61)	1.05 (0.47 to 1.96)	0.7 (0.18 to 1.59)	2.25 (1.03 to 3.79)	2.18 (1.08 to 3.63)	2.31 (0.65 to 4.73)	163.1 (21 to 341.3)	108.5 (-8.6 to 270.7)	232.6 (56.6 to 556.1)
	YLLs	0.81 (0.34 to 1.53)	0.98 (0.44 to 1.86)	0.67 (0.18 to 1.53)	1.97 (0.9 to 3.37)	1.85 (0.93 to 3.09)	2.1 (0.58 to 4.25)	144 (13 to 309.2)	88.7 (-17.5 to 238.6)	214.2 (49.5 to 530.3)
	YLDs	0.05 (0.02 to 0.09)	0.07 (0.03 to 0.14)	0.03 (0.01 to 0.06)	0.27 (0.12 to 0.51)	0.33 (0.13 to 0.64)	0.21 (0.05 to 0.46)	503.7 (157.2 to 960.3)	396.6 (95.9 to 885.8)	700.8 (255.4 to 1547)
Khorasan-e-Razavi	Deaths	0.03 (0.01 to 0.06)	0.04 (0.02 to 0.08)	0.02 (0.01 to 0.06)	0.06 (0.03 to 0.1)	0.06 (0.03 to 0.11)	0.06 (0.02 to 0.11)	102.4 (14.4 to 225.6)	70.4 (-12.2 to 188.7)	149.8 (45.7 to 346.4)
	DALYs	0.77 (0.33 to 1.54)	0.96 (0.42 to 1.93)	0.6 (0.16 to 1.42)	1.65 (0.8 to 2.67)	1.73 (0.9 to 2.85)	1.56 (0.43 to 3.02)	114 (15.2 to 241.9)	80.6 (-12.3 to 202.7)	160.4 (45.6 to 367.2)
	YLLs	0.73 (0.31 to 1.45)	0.9 (0.39 to 1.84)	0.58 (0.15 to 1.37)	1.45 (0.7 to 2.38)	1.47 (0.77 to 2.4)	1.42 (0.4 to 2.76)	98.5 (6.5 to 215.7)	63.5 (-19.7 to 177.5)	147 (39.7 to 345.7)
	YLDs	0.04 (0.02 to 0.09)	0.06 (0.02 to 0.13)	0.02 (0.01 to 0.06)	0.2 (0.09 to 0.37)	0.26 (0.12 to 0.51)	0.14 (0.04 to 0.29)	388.5 (132.1 to 760.1)	330.3 (83.2 to 755.1)	510.3 (200.1 to 1172.6)

Province	Measure	Attributed age-standardized rate (per 100,000)						% Change (1990 to 2019)		
		1990			2019					
		Both	Female	Male	Both	Female	Male	Both	Female	Male
Khuzestan	Deaths	0.04 (0.02 to 0.06)	0.05 (0.02 to 0.09)	0.02 (0.01 to 0.05)	0.07 (0.04 to 0.12)	0.09 (0.05 to 0.15)	0.06 (0.02 to 0.11)	105 (23.1 to 218)	82.5 (-1.2 to 202.2)	160.9 (57.9 to 368.6)
	DALYs	0.94 (0.44 to 1.62)	1.31 (0.65 to 2.34)	0.57 (0.15 to 1.28)	2.06 (1.04 to 3.35)	2.54 (1.23 to 4.25)	1.58 (0.46 to 3.03)	120.4 (31 to 239.6)	93.4 (0.8 to 212.9)	176 (58.5 to 392.5)
	YLLs	0.88 (0.41 to 1.51)	1.22 (0.6 to 2.19)	0.55 (0.15 to 1.23)	1.79 (0.89 to 2.91)	2.14 (1.05 to 3.55)	1.43 (0.42 to 2.78)	104.2 (21.6 to 215.2)	75.9 (-6.9 to 194.6)	161.9 (52.9 to 371.6)
	YLDs	0.06 (0.03 to 0.11)	0.1 (0.04 to 0.19)	0.02 (0.01 to 0.06)	0.27 (0.12 to 0.51)	0.4 (0.16 to 0.78)	0.14 (0.04 to 0.31)	357.3 (150.2 to 663)	312.9 (107.2 to 633.2)	490.3 (201.3 to 1055.5)
Kohgiluyeh and Boyer-Ahmad	Deaths	0.03 (0.01 to 0.05)	0.03 (0.01 to 0.06)	0.02 (0.01 to 0.06)	0.07 (0.03 to 0.12)	0.06 (0.03 to 0.1)	0.08 (0.02 to 0.15)	140.5 (46.5 to 313.4)	74.4 (3 to 205.1)	238.7 (86.9 to 600)
	DALYs	0.72 (0.31 to 1.39)	0.86 (0.38 to 1.6)	0.6 (0.15 to 1.53)	1.89 (0.85 to 3.28)	1.61 (0.86 to 2.73)	2.17 (0.66 to 4.36)	161.3 (61.3 to 342)	88.1 (15.9 to 231.4)	260.6 (95.6 to 635.6)
	YLLs	0.68 (0.29 to 1.3)	0.79 (0.35 to 1.49)	0.58 (0.14 to 1.43)	1.63 (0.72 to 2.84)	1.31 (0.7 to 2.27)	1.94 (0.58 to 3.9)	139.6 (49 to 310.4)	66 (1.6 to 195.5)	236.6 (80.3 to 592.2)
	YLDs	0.05 (0.02 to 0.09)	0.07 (0.03 to 0.14)	0.03 (0.01 to 0.07)	0.27 (0.12 to 0.49)	0.3 (0.13 to 0.56)	0.24 (0.06 to 0.54)	488.4 (234.5 to 950.3)	351.8 (142.8 to 792.9)	770.3 (321.1 to 1877.7)
Kurdistan	Deaths	0.03 (0.01 to 0.05)	0.03 (0.01 to 0.07)	0.02 (0.01 to 0.05)	0.06 (0.03 to 0.09)	0.05 (0.03 to 0.09)	0.06 (0.02 to 0.12)	112.9 (31 to 245)	63.5 (-2.3 to 175.9)	179.8 (60.8 to 438.7)
	DALYs	0.67 (0.28 to 1.32)	0.83 (0.37 to 1.64)	0.54 (0.13 to 1.28)	1.53 (0.74 to 2.57)	1.45 (0.76 to 2.43)	1.62 (0.45 to 3.26)	127.9 (30.3 to 267.1)	73.5 (-1.3 to 188.7)	200.2 (70.2 to 472)
	YLLs	0.63 (0.27 to 1.25)	0.78 (0.35 to 1.53)	0.52 (0.13 to 1.24)	1.35 (0.64 to 2.29)	1.23 (0.65 to 2.04)	1.47 (0.41 to 2.99)	112.6 (21 to 244.5)	57.6 (-10.8 to 164.1)	184.8 (61.6 to 445.5)
	YLDs	0.04 (0.01 to 0.07)	0.05 (0.02 to 0.12)	0.02 (0 to 0.05)	0.18 (0.08 to 0.33)	0.22 (0.1 to 0.41)	0.14 (0.04 to 0.31)	393.7 (151.5 to 819)	300.6 (95.6 to 710.2)	584.6 (249.2 to 1360.6)

Province	Measure	Attributed age-standardized rate (per 100,000)						% Change (1990 to 2019)		
		1990			2019					
		Both	Female	Male	Both	Female	Male	Both	Female	Male
Lorestan	Deaths	0.05 (0.02 to 0.09)	0.07 (0.03 to 0.12)	0.04 (0.01 to 0.08)	0.09 (0.04 to 0.15)	0.08 (0.04 to 0.14)	0.09 (0.03 to 0.19)	66.3 (12 to 155.5)	14.4 (-26.4 to 91.7)	159 (53.9 to 386.9)
	DALYs	1.38 (0.63 to 2.39)	1.86 (0.83 to 3.25)	0.97 (0.25 to 2.22)	2.44 (1.09 to 4.14)	2.32 (0.99 to 4.07)	2.57 (0.72 to 5.14)	76.9 (20.2 to 170.1)	24.6 (-21 to 106.9)	165.7 (52 to 395.8)
	YLLs	1.3 (0.58 to 2.26)	1.73 (0.76 to 3.04)	0.93 (0.23 to 2.15)	2.11 (0.94 to 3.58)	1.91 (0.83 to 3.36)	2.32 (0.65 to 4.62)	62.9 (9.2 to 149.1)	10.6 (-30.5 to 89)	150.3 (42.4 to 370.6)
	YLDs	0.09 (0.04 to 0.16)	0.14 (0.05 to 0.27)	0.04 (0.01 to 0.1)	0.33 (0.14 to 0.62)	0.41 (0.16 to 0.8)	0.25 (0.06 to 0.54)	289.7 (136.9 to 551.2)	202.6 (65 to 470.9)	519.5 (221.8 to 1221.1)
Markazi	Deaths	0.04 (0.02 to 0.07)	0.05 (0.02 to 0.09)	0.03 (0.01 to 0.06)	0.08 (0.04 to 0.14)	0.09 (0.04 to 0.15)	0.08 (0.02 to 0.16)	126.2 (9.1 to 264.6)	84.2 (-14.5 to 211.5)	198.2 (49.7 to 480.3)
	DALYs	0.94 (0.44 to 1.68)	1.17 (0.56 to 2.06)	0.71 (0.19 to 1.64)	2.29 (1.02 to 3.84)	2.33 (1.03 to 4.03)	2.25 (0.64 to 4.49)	145.1 (12.1 to 291.5)	98.8 (-12 to 231.6)	217.4 (51.1 to 518.2)
	YLLs	0.88 (0.4 to 1.57)	1.09 (0.51 to 1.93)	0.68 (0.18 to 1.57)	2 (0.89 to 3.37)	1.96 (0.89 to 3.34)	2.03 (0.59 to 4.03)	127.4 (2.9 to 268.5)	79.7 (-22.5 to 205.6)	199.8 (44.5 to 490.1)
	YLDs	0.06 (0.02 to 0.11)	0.09 (0.04 to 0.17)	0.03 (0.01 to 0.08)	0.3 (0.11 to 0.56)	0.38 (0.14 to 0.75)	0.22 (0.05 to 0.47)	413.5 (110.5 to 805.3)	341.8 (67.5 to 735.1)	601.7 (181.3 to 1381.8)
Mazandaran	Deaths	0.03 (0.01 to 0.06)	0.04 (0.02 to 0.08)	0.02 (0.01 to 0.05)	0.06 (0.03 to 0.1)	0.06 (0.03 to 0.1)	0.06 (0.02 to 0.12)	89 (22.5 to 194.3)	46.7 (-8.5 to 137.1)	163.8 (52 to 394.5)
	DALYs	0.85 (0.41 to 1.58)	1.07 (0.5 to 1.99)	0.63 (0.16 to 1.44)	1.73 (0.87 to 2.8)	1.69 (0.89 to 2.75)	1.77 (0.54 to 3.41)	103.5 (33.1 to 205.6)	57.8 (3.1 to 146.1)	180 (62.5 to 420.3)
	YLLs	0.77 (0.37 to 1.44)	0.96 (0.45 to 1.79)	0.59 (0.15 to 1.33)	1.46 (0.72 to 2.41)	1.36 (0.73 to 2.27)	1.57 (0.48 to 3.02)	88.6 (21.9 to 191.8)	41.8 (-8.2 to 128.3)	163.3 (52.2 to 391.9)
	YLDs	0.07 (0.03 to 0.15)	0.11 (0.05 to 0.22)	0.04 (0.01 to 0.1)	0.27 (0.13 to 0.48)	0.33 (0.16 to 0.61)	0.21 (0.06 to 0.42)	258.7 (123.8 to 501.3)	192.8 (68.6 to 440.7)	442.7 (171.9 to 1038.5)

Province	Measure	Attributed age-standardized rate (per 100,000)						% Change (1990 to 2019)		
		1990			2019					
		Both	Female	Male	Both	Female	Male	Both	Female	Male
**North Khorasan**	**Deaths**	0.04 (0.02 to 0.06)	0.05 (0.02 to 0.09)	0.02 (0.01 to 0.06)	0.07 (0.04 to 0.12)	0.08 (0.04 to 0.14)	0.07 (0.02 to 0.13)	110.8 (33.3 to 238.9)	70.7 (3.8 to 181.7)	187.1 (62 to 442.5)
	**DALYs**	0.93 (0.41 to 1.68)	1.25 (0.55 to 2.28)	0.64 (0.16 to 1.56)	2.04 (1.05 to 3.42)	2.22 (1.17 to 3.73)	1.86 (0.53 to 3.65)	120.2 (38.2 to 252)	77.4 (8.7 to 198.2)	193.2 (62.4 to 453.4)
	**YLLs**	0.88 (0.39 to 1.61)	1.18 (0.52 to 2.17)	0.61 (0.16 to 1.51)	1.81 (0.92 to 3.03)	1.91 (1.02 to 3.2)	1.71 (0.49 to 3.39)	105.2 (28.2 to 229.9)	61.8 (0 to 174.5)	178.8 (56.4 to 425.7)
	**YLDs**	0.04 (0.02 to 0.09)	0.07 (0.03 to 0.15)	0.02 (0.01 to 0.05)	0.23 (0.1 to 0.42)	0.31 (0.14 to 0.58)	0.15 (0.04 to 0.33)	416.9 (188.8 to 803)	341 (132.9 to 753.4)	612.9 (263.6 to 1394.6)
**Qazvin**	**Deaths**	0.03 (0.01 to 0.06)	0.04 (0.02 to 0.08)	0.02 (0 to 0.04)	0.06 (0.03 to 0.1)	0.07 (0.04 to 0.11)	0.05 (0.02 to 0.1)	103.9 (24.5 to 227.9)	55.6 (-15.9 to 162.7)	229 (98.8 to 504.8)
	**DALYs**	0.76 (0.36 to 1.33)	1.12 (0.53 to 2.06)	0.42 (0.12 to 1)	1.67 (0.87 to 2.7)	1.92 (0.92 to 3.23)	1.41 (0.45 to 2.72)	119.2 (30.1 to 245.5)	71.5 (-9.3 to 180.4)	233.3 (95.4 to 525.3)
	**YLLs**	0.71 (0.34 to 1.26)	1.04 (0.49 to 1.92)	0.41 (0.11 to 0.97)	1.44 (0.75 to 2.36)	1.59 (0.78 to 2.66)	1.28 (0.4 to 2.45)	101.6 (20.7 to 225.6)	52.7 (-19.1 to 153.9)	216 (83.7 to 483.2)
	**YLDs**	0.05 (0.02 to 0.09)	0.08 (0.03 to 0.15)	0.02 (0 to 0.04)	0.22 (0.1 to 0.41)	0.33 (0.13 to 0.63)	0.13 (0.04 to 0.28)	398.6 (180.9 to 797.4)	332.5 (111.7 to 776.8)	648.6 (265.1 to 1521.3)
**Qom**	**Deaths**	0.05 (0.02 to 0.1)	0.08 (0.03 to 0.14)	0.03 (0.01 to 0.07)	0.09 (0.04 to 0.14)	0.11 (0.05 to 0.18)	0.07 (0.02 to 0.14)	55.3 (2.9 to 147.5)	38.2 (-15.9 to 135.7)	105.8 (21.8 to 278.9)
	**DALYs**	1.39 (0.63 to 2.46)	1.93 (0.83 to 3.47)	0.88 (0.23 to 1.99)	2.24 (1.03 to 3.74)	2.67 (1.15 to 4.61)	1.85 (0.49 to 3.82)	61.3 (6.4 to 160.2)	38.8 (-15.6 to 134.8)	110.6 (25.9 to 297)
	**YLLs**	1.31 (0.59 to 2.34)	1.8 (0.78 to 3.27)	0.84 (0.22 to 1.91)	1.97 (0.9 to 3.3)	2.28 (0.99 to 3.93)	1.68 (0.45 to 3.41)	49.6 (-2.1 to 146.4)	26.5 (-24 to 116.1)	99.1 (19.5 to 279.7)
	**YLDs**	0.08 (0.03 to 0.14)	0.12 (0.05 to 0.23)	0.03 (0.01 to 0.08)	0.28 (0.1 to 0.52)	0.39 (0.14 to 0.77)	0.17 (0.05 to 0.36)	262.7 (114.4 to 517.3)	219 (78.1 to 486.3)	401.9 (145.4 to 998.9)

Province	Measure	Attributed age-standardized rate (per 100,000)						% Change (1990 to 2019)		
		1990			2019					
		Both	Female	Male	Both	Female	Male	Both	Female	Male
Semnan	Deaths	0.03 (0.01 to 0.06)	0.04 (0.02 to 0.08)	0.02 (0.01 to 0.05)	0.07 (0.03 to 0.12)	0.06 (0.03 to 0.09)	0.09 (0.03 to 0.18)	141.2 (32.9 to 309.2)	53.1 (-17.8 to 176.1)	290.1 (96.7 to 659.9)
	DALYs	0.75 (0.32 to 1.44)	0.9 (0.4 to 1.77)	0.6 (0.15 to 1.36)	1.96 (0.91 to 3.33)	1.55 (0.84 to 2.64)	2.37 (0.66 to 4.67)	160.8 (38 to 331.7)	71.3 (-5.8 to 206.9)	297.6 (94.2 to 659.2)
	YLLs	0.71 (0.29 to 1.36)	0.84 (0.37 to 1.64)	0.57 (0.14 to 1.32)	1.71 (0.77 to 2.94)	1.28 (0.69 to 2.14)	2.15 (0.6 to 4.26)	142.6 (28.6 to 304.6)	52.4 (-16.1 to 173.7)	276.5 (82.9 to 628.9)
	YLDs	0.05 (0.02 to 0.09)	0.07 (0.03 to 0.14)	0.03 (0.01 to 0.07)	0.25 (0.11 to 0.46)	0.27 (0.12 to 0.52)	0.23 (0.06 to 0.47)	438.6 (161.6 to 838.2)	310.2 (96.9 to 691.5)	747.7 (293.9 to 1739.3)
Sistan and Baluchistan	Deaths	0.03 (0.01 to 0.06)	0.03 (0.01 to 0.07)	0.02 (0.01 to 0.06)	0.09 (0.04 to 0.16)	0.08 (0.04 to 0.14)	0.1 (0.02 to 0.21)	225.1 (42.5 to 465.7)	148.6 (-1.6 to 342.6)	313.1 (76.4 to 742.6)
	DALYs	0.71 (0.29 to 1.42)	0.83 (0.35 to 1.69)	0.62 (0.15 to 1.52)	2.58 (1.04 to 4.5)	2.33 (1.11 to 4.14)	2.82 (0.68 to 5.81)	262.1 (53.4 to 535.9)	181.5 (5 to 405.5)	353 (90.8 to 838.3)
	YLLs	0.68 (0.28 to 1.37)	0.79 (0.34 to 1.6)	0.61 (0.14 to 1.49)	2.35 (0.95 to 4.1)	2.07 (0.97 to 3.67)	2.64 (0.65 to 5.49)	243.7 (44.6 to 503.1)	161.4 (-1.7 to 376.3)	336.3 (83.8 to 807.5)
	YLDs	0.03 (0.01 to 0.05)	0.04 (0.01 to 0.08)	0.02 (0 to 0.04)	0.22 (0.08 to 0.42)	0.27 (0.1 to 0.54)	0.18 (0.04 to 0.39)	736.2 (231 to 1457.4)	596.8 (151 to 1388.4)	937.2 (301.1 to 2197.1)
South Khorasan	Deaths	0.04 (0.02 to 0.07)	0.05 (0.02 to 0.1)	0.03 (0.01 to 0.07)	0.08 (0.04 to 0.13)	0.08 (0.04 to 0.13)	0.08 (0.02 to 0.16)	98.1 (30.5 to 208.2)	50.9 (-5.1 to 143.9)	176.2 (54.7 to 447.4)
	DALYs	1.04 (0.44 to 1.9)	1.34 (0.62 to 2.42)	0.77 (0.19 to 1.84)	2.14 (0.94 to 3.58)	2.12 (1.02 to 3.62)	2.16 (0.6 to 4.24)	105.4 (32.3 to 213.6)	57.7 (-1.8 to 147.8)	180.5 (56.9 to 452.9)
	YLLs	0.98 (0.42 to 1.81)	1.25 (0.57 to 2.28)	0.74 (0.18 to 1.78)	1.89 (0.82 to 3.14)	1.8 (0.87 to 3.1)	1.98 (0.54 to 3.88)	91.9 (23.4 to 192.1)	43.8 (-10.1 to 127.8)	166.6 (48.6 to 427.8)
	YLDs	0.06 (0.02 to 0.12)	0.09 (0.04 to 0.18)	0.03 (0.01 to 0.08)	0.25 (0.1 to 0.48)	0.32 (0.12 to 0.6)	0.19 (0.05 to 0.41)	333.1 (158.1 to 611.7)	252.6 (90.5 to 523.7)	528.9 (216.5 to 1250.6)

Province	Measure	Attributed age-standardized rate (per 100,000)						% Change (1990 to 2019)		
		1990			2019					
		Both	Female	Male	Both	Female	Male	Both	Female	Male
Tehran	Deaths	0.06 (0.03 to 0.12)	0.08 (0.03 to 0.15)	0.04 (0.01 to 0.1)	0.07 (0.03 to 0.11)	0.07 (0.04 to 0.12)	0.06 (0.02 to 0.12)	4.2 (-30.2 to 56.8)	-14.1 (-43.3 to 34.7)	48.3 (-13.5 to 173.2)
	DALYs	1.62 (0.69 to 2.9)	2.2 (0.9 to 3.97)	1.03 (0.25 to 2.52)	1.73 (0.77 to 2.86)	1.88 (0.84 to 3.17)	1.59 (0.46 to 3.13)	7.2 (-23.8 to 56.8)	-14.7 (-42.1 to 27.5)	53.5 (-11.8 to 170.1)
	YLLs	1.46 (0.62 to 2.65)	1.96 (0.8 to 3.51)	0.97 (0.24 to 2.36)	1.48 (0.66 to 2.49)	1.55 (0.71 to 2.67)	1.41 (0.41 to 2.85)	1.2 (-30.1 to 52.9)	-20.9 (-47.5 to 21.1)	46.2 (-17.4 to 162.1)
	YLDs	0.15 (0.05 to 0.29)	0.25 (0.09 to 0.48)	0.07 (0.01 to 0.17)	0.25 (0.1 to 0.45)	0.33 (0.13 to 0.61)	0.17 (0.05 to 0.37)	64.2 (6.9 to 160.8)	34.4 (-19.4 to 131.9)	159.5 (32.5 to 430.4)
West Azarbayejan	Deaths	0.03 (0.01 to 0.06)	0.04 (0.02 to 0.08)	0.02 (0.01 to 0.05)	0.06 (0.03 to 0.1)	0.07 (0.04 to 0.11)	0.06 (0.02 to 0.11)	110.1 (25.6 to 229.7)	72.5 (-6.5 to 191.9)	169.9 (61.1 to 377.1)
	DALYs	0.74 (0.33 to 1.43)	0.95 (0.41 to 1.8)	0.55 (0.15 to 1.28)	1.64 (0.83 to 2.68)	1.74 (0.92 to 2.87)	1.52 (0.4 to 2.97)	121.9 (25.8 to 243.5)	82.9 (-6.4 to 204)	179.2 (65.4 to 384.5)
	YLLs	0.7 (0.31 to 1.35)	0.89 (0.39 to 1.69)	0.52 (0.15 to 1.23)	1.44 (0.73 to 2.35)	1.48 (0.77 to 2.43)	1.4 (0.37 to 2.77)	106.8 (17.3 to 222.9)	66.4 (-14.9 to 178.6)	165.8 (56 to 365.4)
	YLDs	0.04 (0.02 to 0.08)	0.06 (0.02 to 0.12)	0.02 (0 to 0.05)	0.19 (0.09 to 0.35)	0.26 (0.11 to 0.48)	0.13 (0.03 to 0.27)	391.5 (130.9 to 761.8)	330.5 (93.6 to 692.9)	523.7 (217.3 to 1170.9)
Yazd	Deaths	0.04 (0.02 to 0.07)	0.05 (0.02 to 0.09)	0.02 (0.01 to 0.06)	0.08 (0.04 to 0.13)	0.09 (0.04 to 0.14)	0.07 (0.02 to 0.14)	113.3 (18.7 to 247.8)	83.3 (-10.9 to 233.9)	183.8 (57.9 to 412.3)
	DALYs	0.9 (0.41 to 1.61)	1.16 (0.51 to 2.16)	0.63 (0.17 to 1.42)	2.08 (1.02 to 3.43)	2.27 (1.08 to 3.9)	1.88 (0.55 to 3.63)	129.6 (20.8 to 261.7)	95.5 (-10 to 241.3)	197.3 (61.5 to 434.2)
	YLLs	0.85 (0.39 to 1.52)	1.08 (0.48 to 2.02)	0.61 (0.17 to 1.35)	1.78 (0.87 to 2.96)	1.86 (0.88 to 3.2)	1.69 (0.49 to 3.27)	109.3 (13.7 to 229)	72.9 (-17.9 to 211.6)	178 (53 to 414.1)
	YLDs	0.06 (0.02 to 0.11)	0.08 (0.03 to 0.17)	0.03 (0.01 to 0.07)	0.3 (0.14 to 0.57)	0.41 (0.17 to 0.8)	0.2 (0.05 to 0.42)	442.9 (145.6 to 864.7)	385.1 (103.6 to 868)	629.9 (250.5 to 1472.3)

Province	Measure	Attributed age-standardized rate (per 100,000)						% Change (1990 to 2019)		
		1990			2019					
		Both	Female	Male	Both	Female	Male	Both	Female	Male
Zanjan	Deaths	0.02 (0.01 to 0.04)	0.03 (0.01 to 0.06)	0.02 (0 to 0.04)	0.05 (0.03 to 0.08)	0.05 (0.02 to 0.08)	0.05 (0.02 to 0.11)	141.6 (31.8 to 297.5)	74.1 (-6.9 to 208.8)	260.1 (95.6 to 595.7)
	DALYs	0.51 (0.22 to 1.06)	0.65 (0.27 to 1.44)	0.39 (0.1 to 0.93)	1.31 (0.65 to 2.21)	1.22 (0.64 to 2.01)	1.4 (0.42 to 2.72)	154.6 (35.8 to 308.2)	86.7 (-3.2 to 210)	261 (86.3 to 583.8)
	YLLs	0.49 (0.2 to 0.99)	0.61 (0.25 to 1.33)	0.37 (0.1 to 0.9)	1.16 (0.57 to 1.95)	1.03 (0.54 to 1.72)	1.28 (0.38 to 2.48)	137.9 (27.1 to 285.3)	69.1 (-11.2 to 185.6)	243.9 (79 to 557.9)
	YLDs	0.03 (0.01 to 0.06)	0.04 (0.02 to 0.1)	0.02 (0 to 0.04)	0.15 (0.07 to 0.28)	0.19 (0.08 to 0.35)	0.12 (0.03 to 0.26)	439 (163.2 to 871.7)	338.6 (105.3 to 743.4)	666.8 (275.9 to 1475)

*Data in parentheses are 95% Uncertainty Intervals (95% UIs).
